# Hatchlings of *Tyrannosaurus rex* and the Evolution of Dinosaur Reproductive Strategies

**DOI:** 10.3390/biology15131090

**Published:** 2026-07-07

**Authors:** Nicholas R. Longrich, Peter J. Makovicky, Tim Tokaryk, David M. L. Cooper, Evan T. Saitta, Gregory M. Erickson, Tamas Szekely, Eric Snively

**Affiliations:** 1 Department of Biology and Biochemistry, University of Bath, Bath BA2 7AY, UK; bssts@bath.ac.uk; 2 Department of Earth and Environmental Sciences, University of Minnesota, Minneapolis, MN 55455, USA; pmakovic@umn.edu; 3 Department of Earth Sciences, University of Regina, Regina, SK S4S 0A2, Canada; tim.tokaryk@gmail.com; 4 Department of Anatomy, Physiology and Pharmacology, University of Saskatchewan, Saskatoon, SK S7N 5SA2, Canada; dml.cooper@usask.ca; 5 Field Museum of Natural History, Integrative Research Center, Life Sciences Section, Chicago, IL 60605, USA; evansaitta@gmail.com; 6 Department of Biological Science, Florida State University, Tallahassee, FL 32306, USA; gerickson@bio.fsu.edu; 7 Oklahoma State University College of Osteopathic Medicine at the Cherokee Nation, Tahlequah, OK 74464, USA

**Keywords:** Dinosauria, Tyrannosauridae, *Tyrannosaurus rex*, *Gorgosaurus libratus*, parental care, evolution

## Abstract

Tyrannosaurs were giant predatory dinosaurs that occupied the apex of Late Cretaceous food chains. Little is known about the early life and reproductive ecology of tyrannosaurs due to the extreme rarity of hatchling and juvenile fossils. We report bones of hatchlings (<1 yr) for *Tyrannosaurus rex* and *Gorgosaurus libratus*, weighing ~2.5 kg and ~2.4 kg, respectively, i.e., <0.1% of adult mass. Clutches were likely large. We conservatively estimate ~20 eggs in a small adult *T*. *rex* versus ~30 eggs in the largest *T*. *rex*, and clutches of ~15 eggs in *G*. *libratus*; larger clutches of 50 or even 100 eggs are not impossible. This suggests an *r*-selected reproductive strategy. Synchrotron scans reveal Haversian bone remodeling, suggesting that tyrannosaurs moved soon after hatching. Hatchling tyrannosaurs’ small size and precociality suggest limited parental care; teeth of hatchlings show wear suggesting that they fed on relatively large vertebrates. Tyrannosaurs had proportionately larger offspring than most reptiles, but relatively smaller hatchlings than Eumaniraptora and birds, suggesting retention of a primitive reproductive strategy intermediate between that of basal diapsids and birds. Multiple dinosaur lineages evolved large eggs independently, suggesting an evolutionary trend towards increased parental investment.

## Simple Summary

Dinosaurs were intermediate between more primitive reptiles and modern birds in their anatomy, and in some aspects of their biology. Their reproductive strategies remain poorly understood because fossils of young dinosaurs and eggs are rare. We report fossils of hatchling *T*. *rex* and other tyrannosaurs that help us understand the reproductive strategies of these animals. Tyrannosaurs had relatively small hatchlings compared to modern birds, suggesting they laid large numbers of eggs and provided relatively little care for hatchlings. Dinosaur reproductive strategies were likely intermediate between those of more primitive reptiles, with limited parental investment, and the advanced parental care strategies and intensive investment seen in modern birds.

## 1. Introduction

Tyrannosaurids were carnivorous theropods characterized by large and massively built skulls, short forelimbs, and elongate hindlimbs [1,2]. They diversified in the Late Cretaceous [2,3,4], culminating in the evolution of the giant *Tyrannosaurus rex*, which was among the largest terrestrial predators of all time. Tyrannosaurids are among the best-studied dinosaurs; previous research has examined their evolution, feeding [5,6], biomechanics [6], and growth [7,8,9,10]. Yet tyrannosaur reproduction and early development are poorly understood due to pervasive biases against preservation, collection, and study of small dinosaurs [11].

Early ontogenetic stages are exceptionally rare for tyrannosaurs [12,13]. No bones of neonates and only one embryonic jaw [13] can be unambiguously assigned to Tyrannosauridae (see Supplementary Materials), along with a few teeth [13,14]. The small, often isolated bones of hatchlings and embryos are often overlooked in the field. Even when they are collected, these remains often remain unidentified, and unstudied. As a result, much remains unknown about young tyrannosaurs. Were neonates nestbound, or mobile? What did they eat? How large were eggs and clutches, and how fast did hatchlings grow?

While small vertebrate skeletons are rare, the Late Cretaceous of North America has a remarkably rich record of isolated vertebrate microfossils that can potentially provide information on these problems [11]. We surveyed museum collections for skeletal remains and shed teeth to reveal the youngest tyrannosaurs yet described. These fossils constrain the size of hatchling tyrannosaurs and provide unique insights into their reproductive biology.

## 2. Materials and Methods

### 2.1. Scanning

We scanned specimens using phase-contrast synchrotron microCT to enhance edge detail resolution over attenuation-based scanning. All scans were carried out on the BMIT ID beamline at the Canadian Light Source synchrotron (Saskatoon, SK, Canada). In addition to small juvenile tyrannosaurs, we scanned portions of third metatarsals of a larger juvenile *T. rex* (RSKM 2641.1: Frenchman Formation, Saskatchewan), and a drill core of an adult *G. libratus* (UALVP 49310: Dinosaur Park Formation, Alberta). Specimens were mounted in X-ray-opaque plexiglass cylinders. Scanning was at 4.4 micron resolution at an energy of 50 keV, with 80–500 ms exposure times (longer for RSKM 2641.1).

### 2.2. Reconstruction

Raw radiographic output was processed into tomographic (slice) data on SkyScan high-performance computing clusters, with custom macros in NRecon and ImageJ 1.52k [15]. Minimal processing was necessary for ring artifact reduction. Slice data was reduced as needed from 16-bit to 8-bit for visualization. We visualized volumes and separated bone from canals in Amira and Avizo (Thermo Fisher Scientific, Waltham, MA, USA) using a custom workflow. Darker gray values for canals were thresholded within light-gray thresholded-and-filled bone mineral. Canals were reconstructed as isosurfaces and visualized within volume renderings of visually informative opacity and transparency. See Supplementary Materials for detailed reconstruction and scanning protocols.

Individual 4.4 micron thick “slice” reconstructions are thinner than physical ground thin sections, and difficult to interpret based on traditional classifications of bone tissue type. We used the thick stack function in ImageJ to overlay 15 individual slices, replicating the thickness of a ground thin section. This enabled us to test whether perinates were embryonic, post-hatching, or mature by discriminating between the coarse latticework of embryonic bone and the compacted, fibrolamellar appearance of post-hatching bone.

### 2.3. Bone Density

Wolff’s Law [16,17] describes how bones remodel under stress, becoming both thicker and denser in response to mechanical loading. The radical changes in patterns of bone loading from embryo to hatchling, which exceed those seen at any other time of life, are therefore predicted to produce changes in bone structure, particularly bone density, as hatchlings transition from effectively motionless in the egg to experiencing significant loading and strain after hatching and locomotion. CT scans of titanosaur hatchlings show that bone density increases around the time of hatching and progressively increases as the hatchling grows (Supplementary Materials).

To characterize bone density changes, a series of 15 CT slices were stacked, creating a 60 µ section equivalent to a thin section. Gray values of the slices were measured using the “Plot Profile” and mapped using the “Surface Plot” functions of ImageJ 1.52k [18]. Gray value functions as a proxy for bone density; although differences in preservation, scanning and post-image processing make comparisons of absolute values between different fossils difficult, they are used here to examine density differences within a single fossil to examine ontogenetic changes in bone density. We verified this approach by testing it on a hatchling titanosaur [19], which shows changes in bone density associated with the hatching line [19].

### 2.4. Estimation of Reproductive Parameters

Body mass for juvenile tyrannosaurids was estimated using the regression of body mass against the respective widths, lengths, and areas of the distal articulation of metatarsal III for 78 bird species, using both Ordinary Least Squares (OLS) and Phylogenetic Generalized Least Squares (PGLS) regressions. We used the best-fitting regression (OLS, area) as determined by AICcs score to estimate body mass of the two neonate tyrannosaur specimens along with their 95% prediction intervals and used crocodilian (65%) and avian (67%) estimates of hatchling to total egg mass to estimate egg mass in *T. rex*.

We compared egg mass estimates from the dimensions of RSKM P2416.82 against predictions of egg size derived from avian and crocodilian models that phylogenetically bracket the possible range of masses in non-avian dinosaurs. In order to predict egg size in *T. rex*, we regressed log-transformed egg mass against log-transformed body mass for both clades, with body and egg mass data taken from a recent comprehensive data set for amniotes [20]. Our extrapolations for egg size in *T. rex* are based on the estimated body mass of MOR 1125 of 6100 kg [21], which was identified as a gravid female due to the presence of medullary bone [22], and for FMNH PR 2081, as one of the largest and most complete specimens [23].

For all avian PGLS regressions, we employed the Jetz et al. [24] phylogeny because it includes most of the extant avian biodiversity. We sampled a single tree with the Hackett et al. [25] ordinal-level phylogeny as a backbone from the 1000 trees in the data set available on Dryad to expedite processing time. For crocodilians, we selected the recent Colston et al. [26] phylogeny, as it has the most complete sampling of extant taxa. Branch lengths were left unmodified in the source trees. The degree of phylogenetic signal in the observed body mass and egg mass data was assessed using the K metric [27]. We evaluated the model fit to the data using log likelihood scores with a ∂Loglikelihood ≥ 2 taken as significant and ≥10 as highly significant following common practice in systematics. We used the better-fitting model (either OLS or PGLS) for each clade to estimate the 95% prediction interval for the *T. rex* egg mass and clutch mass estimates.

We also used OLS regressions of crocodilians and of all diapsids to extrapolate clutch mass for *T. rex,* as there is a strong relationship across clades, and we had no well-sampled, calibrated diapsid phylogeny to use for PGLS. All analyses were performed in R statistical software (ver. 4.2.1, R Development Group 2022) through the R Studio interface.

### 2.5. Growth Curves

Growth curves were produced in R (version 4.1.1), whereby each tyrannosaur specimen had a body mass estimate and an age at death estimate based on the number of LAGs, collated and corrected as required from the literature (Table S4). Logistic growth models were applied to each species, with consideration of controversial tyrannosaur taxonomies, and best-fitting curves were corrected via vertical transformation along the axis of body mass based on hatchling mass estimates reported herein (see Supplementary Materials). Parameters of the growth curves of extant taxa were obtained from the literature (see Supplementary Materials).

## 3. Results

### 3.1. Systematic Paleontology

Dinosauria Owen 1842 [28].

Theropoda Marsh 1881 [29].

Coelurosauria von Huene 1914 [30].

Tyrannosauridae Osborn 1906 [31].

Tyrannosaurinae Osborn 1906 [31].

*Tyrannosaurus rex* Osborn 1905 [32].

*Material*. FMNH (Field Museum of Natural History) PR 4920 partial dentary; Hell Creek Formation, Buffalo, South Dakota. YPM (Yale Peabody Museum) 55604, 55570, 55590, incisors; YPM 55564, 55567, 55020, lateral teeth; Lance Formation, Wyoming. RSKM (Royal Saskatchewan Museum) P2416.82, metatarsal III; Frenchman Formation, Saskatchewan. All latest Maastrichtian (Lancian) in age [33].

**Figure 1:**
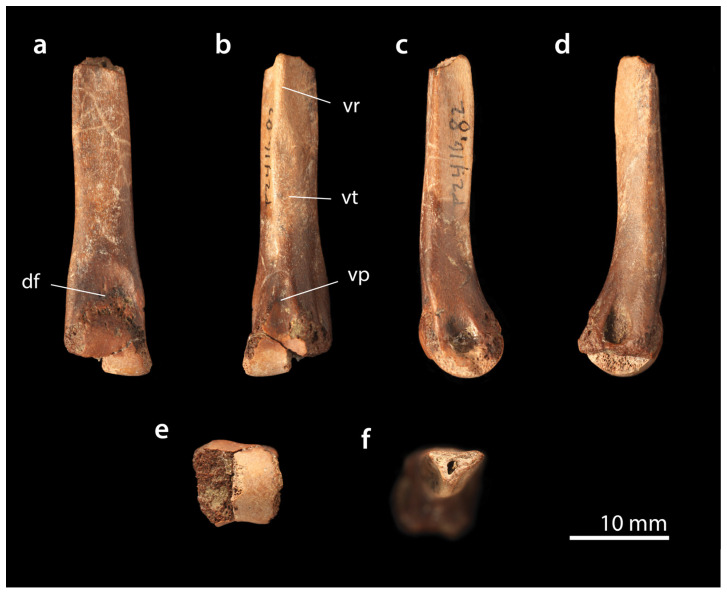
*T. rex*, left metatarsal III, RSKM P2416.82, latest Maastrichtian Frenchman Formation of Saskatchewan: (**a**) anterior, (**b**) posterior, (**c**) medial, (**d**) lateral, (**e**) distal, and (**f**) proximal views. Abbreviations: df, dorsal fossa; pl, platform; vp, ventral pillar; vr, ventral ridge.

*Metatarsal III.* A diminutive left third metatarsal, RSKM P2416.82 (Figure 1a–f), differs from all other theropod clades known from the Late Cretaceous (Figure S1) and shows a unique combination of derived characters diagnostic of Tyrannosauridae, specifically Tyrannosaurinae (Figure S2). Histology (see below) suggests that the specimen pertains to a neonate (<1 year old).

The shaft is very narrow proximally but expands distally to overlap metatarsals II and IV as in Tyrannosauridae [34] and convergently in Ornithomimidae [35,36], Alvarezsauridae [37], Troodontidae [33], Caenagnathidae [38] and Microraptorinae. The shaft is asymmetrical, with the convex flange overlapping metatarsal II being larger and more proximally placed than the flange overlapping metatarsal IV. This feature is only weakly developed in small juvenile Tyrannosauridae [39] but becomes exaggerated in large adult tyrannosaurids [34,39], where it gives the metatarsal a twisted or sigmoidal appearance (Figure S2). This asymmetrical, twisted shaft is not seen in other arctometatarsalian theropods [35,37,38] and helps diagnose the metatarsal as tyrannosaurid.

Below the articulations with II and IV, the shaft expands where it forms the phalangeal articular surface. Just proximal to the articular surface, the metatarsal shaft bears a deep U-shaped dorsal pit or extensor depression. This U-shaped fossa is characteristic of Tyrannosauridae, including *Tyrannosaurus* [34], *Daspletosaurus* [39], and *Albertosaurus* [39]; it also occurs in Nanotyrannidae [40]. The extensor depression is weakly developed in Alioraminae [41] and is shallower and more dorsally extended in Troodontidae [42]. In Ornithomimidae, there is only a very shallow, poorly defined depression, not a deep, discrete fossa [36]. The flexor pit is absent in Caenagnathidae [43], and Alvarezsauridae [37].

In lateral or medial view, the distal end of the shaft of metatarsal III and the condyle are strongly deflected ventrally relative to the proximal shaft (Figure 1c,d). This condition is shared with *T*. *rex* [34] and is weakly developed in *Daspletosaurus* [39], but absent in *Nanotyrannus* [40] and Alioramini, where the distal shaft is straight [41]. Other coelurosaurs have a straight shaft with limited ventral deflection.

On the ventral surface of metatarsal III, there is a prominent longitudinal ridge between the articulations for metatarsals II and IV (Figure 1b). In most arctometatarsalian theropods, this ridge is typically sharply keeled, e.g., Alvarezsauridae [37], or broadly rounded, e.g., Ornithomimidae [36] and Albertosaurinae [44]. In RSKM P2416.82, the ridge is sharp proximally but distally forms a distinct pillar-shaped swelling or tubercle. This pillar-shaped ridge is a synapomorphy of Tyrannosaurinae, which is present in *Daspletosaurus* [39], *Bistahieversor* (NRL pers. obs.) and *Tyrannosaurus* [34], but not Albertosaurinae [39] or other basal Tyrannosauridae such as Nanotyrannidae (BMRP 2002.4.1), where the ridge remains sharp distally.

Distal to the ventral pillar, two ridges extend to connect the ventral pillar and the articular surface (Figure 1b). The two ridges delimit a triangular platform with a depressed area between them. A similar triangular platform occurs in young *T. rex* (RSKM P.2643) and juvenile *Daspletosaurus* [39]. In adult *T*. *rex,* the ridges are present as well, but the platform between them is broader and has a flat surface [34]; in *Albertosaurus,* the platform is relatively small [39].

The articular surface is well-developed. It has a strongly U-shaped proximal margin in dorsal view (Figure 1a), with its apex slightly offset, again resembling the condition in tyrannosaurids [34,39]. The proximal margin of the articular surface is strongly U-shaped and symmetrical in Caenagnathidae [43], weakly convex in Ornithomimidae [36] and Alvarezsauridae [37], and B-shaped in Troodontidae [42].

The articular surface is as wide as tall in distal view (Figure 1e), as in Tyrannosauridae [34], Caenagnathidae [43] and Alvarezsauridae [37]. The articular surface is wider than tall in Ornithomimidae [36], and taller than wide in Dromaeosauridae [45] and Troodontidae [42]. The distal articular surface lacks a ginglymus condyle, in contrast to Deinonychosauria [42,45].

In lateral or medial view (Figure 1b,c), the articular surface is subcircular. The condyle is dorsally projected relative to the distalmost end of the shaft. This feature is typical of Tyrannosauridae [34], whereas the condyle does not project above the shaft in Nanotyrannidae [40].

The combination of characters seen here—including a pinched MT III with a sigmoidal shape, a ventral pillar, a triangular ventral platform, a strong dorsal extensor depression, a ventrally deflected distal shaft, an asymmetrical U-shaped proximal margin to the distal condyle, and a condyle about as tall as wide in distal view—is unique to Tyrannosauridae among all known theropod families. The ventral pillar and strong deflection of metatarsal III are seen only in Tyrannosaurinae and most closely resemble *Tyrannosaurus* (Figure S2), the only tyrannosaurine currently known from the Late Maastrichtian of the Western Interior. Specimens referred to *Nanotyrannus* (BMRP 2002.4.1) lack the ventral pillar seen here and in adult *T*. *rex* [46] or the strong ventral deflection of the distal end of metatarsal III and the dorsal projection of the distal condyle in lateral view.

**Figure 2:**
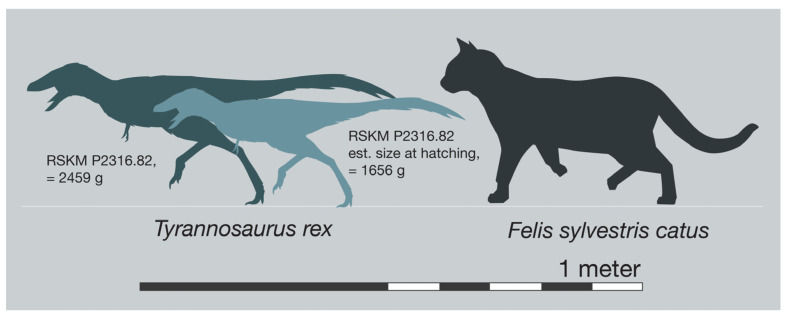
Size of perinate *T. rex*. Reconstruction based on the third metatarsal RSKM P2416.82, assuming proportions of a juvenile *Tarbosaurus baatar* [12]; post-hatching individual scaled to 84% of the linear dimensions of RSKM P2416.82.

*Comparisons:* RSKM P2416.82 comes from an animal approximately 75 cm long (Figure 2), based on comparisons with juvenile tyrannosaurines [12]. To more precisely estimate the specimen’s size, we used regressions of body mass against metatarsal dimensions in extant birds to predict body mass (Supplementary Materials Data S1; Table S2). The mean mass estimated from the condyle area (8 mm × 7.5 mm) is 2459 g (95% cPI: 1229–5623 g: Supplementary Materials Table S2). The use of distal condyle area to estimate mass is appropriate because both specimens fall within the size range of birds used in the regression, because the condyle area is tightly correlated with body mass in extant birds (Supplementary Materials), because distal articular surface areas correlate strongly with mass in related animals with similar gait kinematics [47,48], and metatarsus kinematics were likely similar in birds and non-avian theropods [49].

Hatchling mass averages 65% egg mass in crocodilians [50] and 67.2% egg mass in birds [51]. Based on the previous body mass estimate, predicted egg masses (Table S6) are 3783 g (95% cPI 1945–7358 g) and 3659 g (95% cPI 1881–7118 g), respectively, for RSKM P2416.82.

These mass estimates will be overestimates, assuming the animal underwent substantial growth in the days or weeks after hatching and prior to death. While the hatching line itself is not visible in the synchrotron scans, distinct changes in density are found between the inner and outer cortex. A change in bone density (as measured by brightness in the CT scans) occurs about two-thirds of the way through the cortex. This suggests that the inner two-thirds of the cortex is embryonic bone and the outer one-third is hatchling bone, with the embryo–hatchling transition approximately 2/3 of the way through the bone (see below). We estimate that the bone diameter at hatching was about 84% of the bone diameter at the time of death. If we rescale the dimensions of the condyle accordingly, then the estimated hatchling mass becomes 1656 g for RSKM P2416.82. The corresponding egg mass estimates are then 2547 g (crocodilian egg model) and 2463 g (bird egg model).

**Figure 3:**
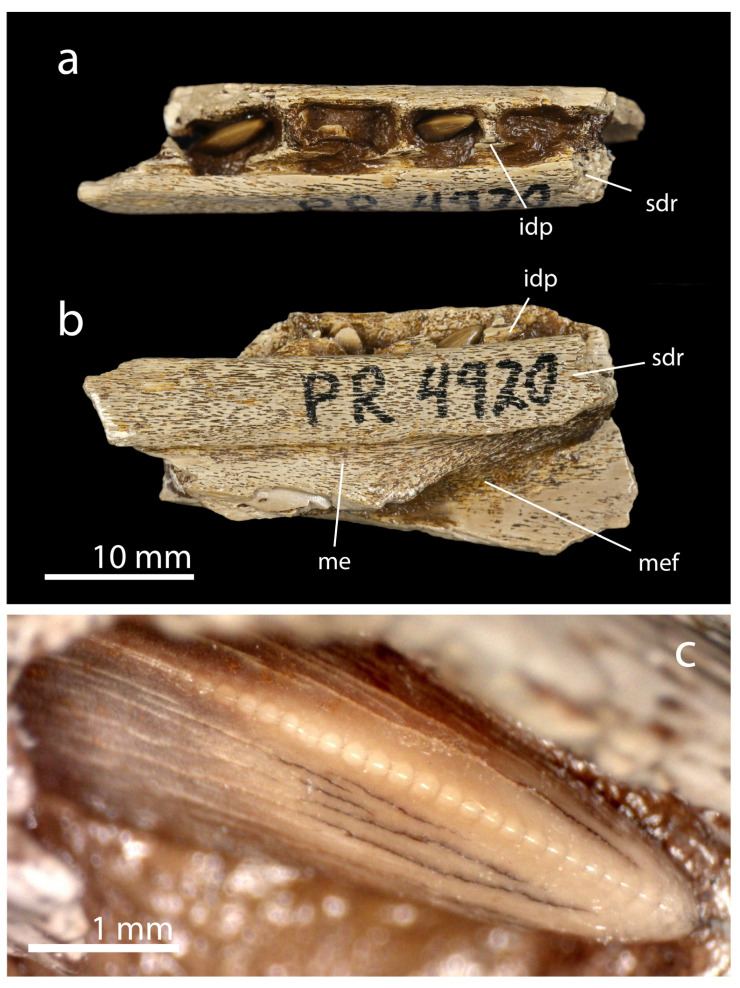
Partial dentary of young *T. rex*, in (**a**) dorsal, (**b**) medial views; (**c**) closeup of posterior tooth showing large denticles typical of *T*. *rex.* Abbreviations: idp, interdental plate; me, Meckelian groove; mef, Meckelian fossa; sdr, subdental ridge.

*Dentary.* A partial dentary, FMNH PR 4920, preserves the posterodorsal margin of the jaw. It bears four alveoli which contain three partially erupted teeth (Figure 3). The bone surface shows a striated texture of grooves for blood vessels, as is characteristic of young, rapidly growing dinosaurs [10,52].

Alveoli are broad and subrectangular when seen in dorsal view, as in *T. rex*. The alveoli are bounded laterally by the dentary parapet; medially they are bounded by large interdental plates, and below that, by the subdental ridge or lingual bar. The subdental ridge is relatively narrow; *T. rex* has a subdental ridge that is extremely deep anteriorly and very narrow posteriorly [32,34]. In *Nanotyrannus* (Figure S3)*,* the subdental ridge is similar in depth anteriorly and posteriorly [46]. The subdental ridge extends far up the medial surface of the jaw so that the alveoli are largely obscured, while the interdental plates are small. Again, this feature is shared with *T*. *rex* [46]; in *Nanotyrannus,* the subdental ridge is more ventrally positioned such that alveoli are broadly exposed in medial view, and the interdental plates are taller as well [46]. Crowns in FMNH PR 4920 are robust, being broad labiolingually, and have large mesial serrations near the tip. The coarse denticles help distinguish the teeth from *Nanotyrannus* [53].

**Figure 4:**
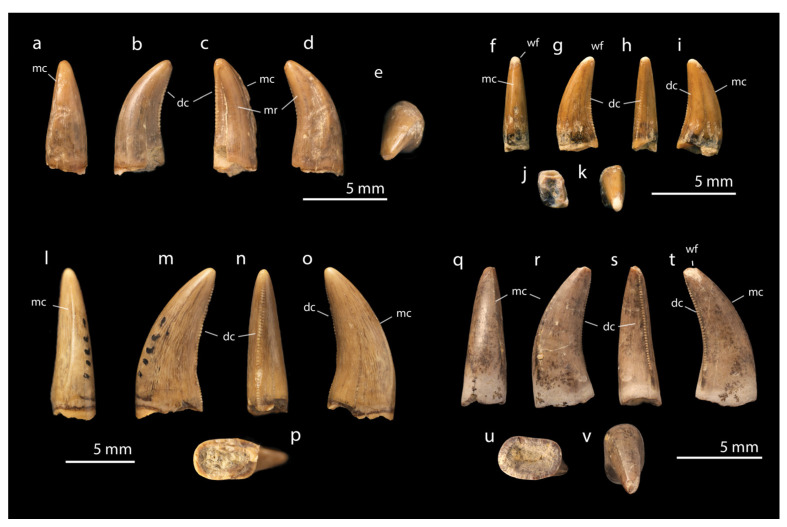
Hatchling *T. rex* teeth, late Maastrichtian Lance Formation, Wyoming. (**a**–**e**) YPM 55604, premaxillary tooth in (**a**) anterior, (**b**) lateral, (**c**) posterior, (**d**) medial, and (**e**) apical views. (**f**–**k**) YPM 55564, lateral tooth in (**f**) anterior, (**g**) lateral, (**h**) posterior, (**i**) medial, (**j**) basal views and (**k**) ventral views. (**l**–**p**) YPM 54474, lateral tooth in (**l**) anterior, (**m**) medial, (**n**) posterior, (**o**) lateral, and (**p**) basal views. YPM 55551, lateral tooth in (**q**) anterior, (**r**) medial, (**s**) posterior, (**t**) lateral, (**u**) basal and (**v**) apical views. Abbreviations: dc, distal carina; mc, mesial carina; mr, mesial ridge; wf, wear facet.

*Teeth*. Isolated teeth (Figure 4) from the Lance Formation of Wyoming can be referred to Tyrannosaurinae based on the shape of crowns and denticles. In combination with provenance, this allows referral to *T*. *rex*.

Premaxillary teeth (Figure 4a–e) are robust, with their labiolingual thickness approaching their mesiodistal width. Crowns are weakly curved. They have the characteristic D-shaped section of tyrannosaurids [54], in which the mesial carinae twist onto the crown’s lingual surface to orient posteriorly. The crown’s lingual surface bears a distinct ridge running apico-basally as in other tyrannosaurids [54]. The apicobasal ridge is broad, and narrow channels separate this ridge from the carinae. In contrast, in Albertosaurinae [55] and Nanotyrannidae (BMRP 2002.4.1), the apicobasal ridge is narrower, and is flanked by broad gutters separating the ridge from the carinae. The apex of the premaxillary tooth is pointed as in other tyrannosaurines, including *T. rex* [54], as well as adult [56] and juvenile *Tarbosaurus baatar* [12]; the crown lacks the straight, blunt, chisel-shaped apex of Albertosaurinae [55,57] or *Nanotyrannus* [40,46].

In the premaxillary tooth described here, the mesial carina is unserrated, and the distal carina is serrated. In adult Tyrannosaurinae, both carinae are always serrated [54], and this is also the case in a young *Tarbosaurus baatar* [12]—which represents a young juvenile, but not a hatchling. Meanwhile, an embryonic tyrannosaurid jaw lacks serrations on all teeth [13]. This suggests that serration patterns can change over ontogeny in Tyrannosauridae. Nanotyrannid teeth have been described as lacking serrations in the premaxillary teeth [40,46], although a few denticles are weakly developed near the base of the distal carina in the premaxillary teeth of BMRP 2002.41 (NL pers obs.).

Lateral teeth (Figure 4f–v) are robust despite their small size; in basal view, the crown has an ovoid section, and the crown’s labiolingual diameter is more than 50% of the mesio-distal diameter. Robust crowns are typical of *Tyrannosaurus*; *Nanotyrannus* has more bladelike teeth with a narrower cross-section. Dromaeosaurid teeth are labiolingually compressed and have a “pinched” or figure-8 section where fullers run along the base of the crown and down to the root.

The crowns are weakly curved and taper at the apex. The narrow, pointed, “railroad spike” tooth apex is typical of *T. rex*, particularly in anterior maxillary and dentary teeth [54]. By contrast, the crowns of *Tarbosaurus baatar* tend to be slightly more bladelike [56], as are those of *Nanotyrannus* [46].

Mesial carinae twist medially in anterior view towards the base of the crown; distal carinae are deflected laterally towards the base; these twisted carinae characterize tyrannosaurids [54]. As with the premaxillary tooth, the lateral teeth bear unserrated mesial carinae, but serrated distal carinae. In somewhat larger specimens, e.g., FMNH PR 4920, both the anterior and posterior carinae are serrated, suggesting ontogenetic change in serration, with serrations developing first on the distal carinae, and later on the mesial carinae.

Tooth wear is visible on the apices of YPM 55564 and 55551. Enamel is worn away to expose dentine at the tooth apex in both teeth and tooth wear along the mesial carina in YPM 55564 and onto the lateral surface of the apex in YPM 55551. Such tooth wear is typical of tooth wear described for teeth of large Tyrannosauridae [58]. Dromaeosaurids typically have minimal tooth wear [59], while tooth wear in Troodontidae is typically seen on the face of the crown as large, well-developed wear facets [60]. The similarity between the tooth wear seen here and in other Tyrannosauridae provides additional support for referral to Tyrannosauridae.

The teeth can be identified as neonates rather than embryos on the basis of this tooth wear, indicating feeding, and the fact that the root has been resorbed, showing the teeth were used and then shed.

Similar juvenile/hatchling teeth have been previously reported from the Lance Formation as Tyrannosauridae [14] and are here referred to *T*. *rex*.

Albertosaurinae Currie, Hurum & Sabath 2003

cf. *G. libratus* Lambe 1914

**Figure 5:**
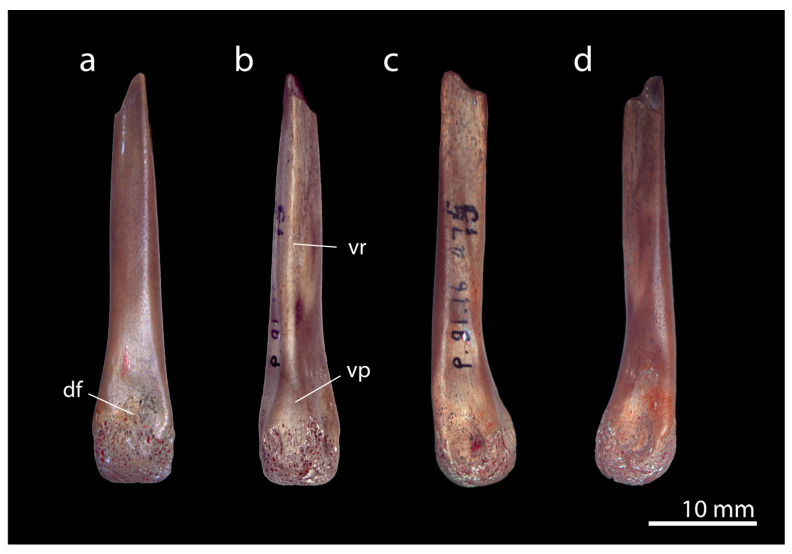
*G. libratus*, left metatarsal III, TMP 1981.16.475, middle Campanian Dinosaur Park Formation of Alberta, in (**a**) dorsal, (**b**) ventral, (**c**) lateral, and (**d**) medial views. Abbreviations: df, dorsal fossa; vp, ventral platform; vr, ventral ridge.

*Metatarsal III.* TMP 1981.16.475, a small third metatarsal from the Dinosaur Park Formation of Alberta, Canada, (Figure 5), shows a combination of features allowing referral to Tyrannosauridae. Furthermore it shows a character combination unique to Albertosaurinae among tyrannosaurs known from the Campanian of Alberta.

The shaft of the metatarsal is pinched as in other tyrannosaurs. The asymmetry seen in the shaft in young *T*. *rex* and adults of other species is present, but only incipiently developed. The shaft is distinctly more slender than in the hatchling *T. rex* (Figure 1) and in this feature resembles *G. libratus* (Figure S2), which has a relatively slender metatarsus. As with juvenile *T*. *rex* and other Tyrannosauridae, there is a distinct pit on the dorsal surface of the metatarsal proximal to the articular surface.

The distal end of the shaft is deflected ventrally (Figure 5c,d), but only weakly, in contrast to the strong ventral deflection of metatarsal III in *T. rex* RSKM P2416.82.

On its ventral surface, TMP 1981.16.475 bears a strong, sharp proximodistal ridge extending nearly to the end of the bone (Figure 5b); the ventral surface lacks the ventral pillar seen in *T*. *rex* and other tyrannosaurinae. There is a small triangular ventral platform between the ventral ridge and the condyle, but it is narrow and lacks the strong lateral ridges and depression seen in *T*. *rex*.

The combination of characters seen here allows referral to Tyrannosauridae. However, the absence of the ventral tubercle and the very gracile construction of the metatarsal argue against referral to Tyrannosaurinae. Instead, this combination of features is characteristic of Albertosaurinae. This argues for tentative referral of TMP 1981.16.475 to *Gorgosaurus libratus*, the only albertosaurine currently named from the Dinosaur Park Formation [61].

**Figure 6:**
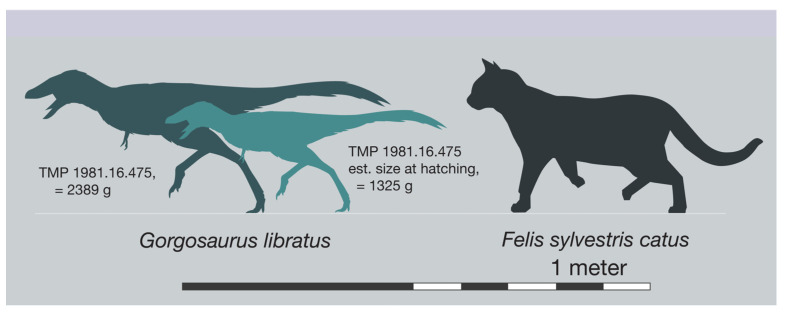
Size of perinate *G. libratus*. Reconstruction based on third metatarsal TMP 1981.16.475, post-hatching individual scaled to 77%.

Condyle area (7.5 mm × 7.8 mm) suggests a body mass of 2389 g and egg mass of 3676 g (crocodilian model) or 3555 g (avian model), assuming no growth. Histology suggests a transition from embryonic bone to hatchling bone about 50% of the way through the metatarsal cortex. The diameter of the third metatarsal at hatching was accordingly estimated at 77% of the diameter of the bone at the time of death, suggesting the animal was slightly older than the *T*. *rex* hatchling when it died. If we downscale the metatarsal linear dimensions accordingly, we arrive at a body mass of 1325 g and egg masses of 2039 g using a crocodilian model and 1972 g using a bird model. Body length of TMP 1981.16.475 is estimated as ~70 cm (Figure 6); immediately post-hatching, it is estimated to have been ~50 cm.

Nanotyrannidae

Nanotyrannidae indet.

**Figure 7:**
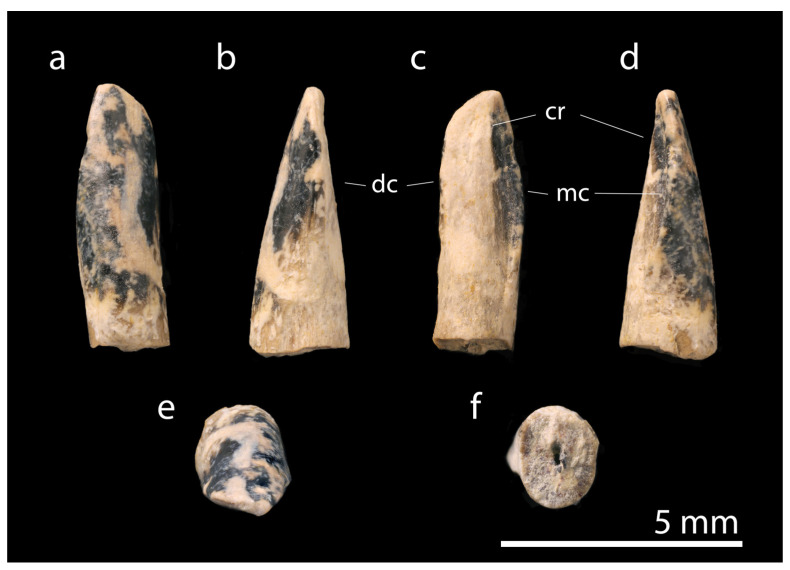
Hatchling tooth, Nanotyrannidae, late Maastrichtian Lance Formation, Wyoming. (YPM 55587; premaxillary tooth in (**a**), anterior, (**b**), lateral, (**c**), posterior, (**d**), medial, (**e**), apical view, (**f**) basal view. Abbreviations: cr, central ridge; dc, distal carina; mc, mesial carina.).

*Tooth.* A premaxillary tooth, YPM 55587 (Figure 7), closely conforms to the morphology of Nanotyrannidae [46]. Several distinct taxa of Nanotyrannidae occur in the late Maastrichtian. The number of taxa and their stratigraphic distribution is unclear, but they include *Nanotyrannus lancensis* [46,62,63], possibly *Stygivenator molnari* [46], and *Nanotyrannus* (=*Stygivenator*?) *lethaeus* [40]; accordingly, this fossil is simply identified as Nanotyrannidae indet.

The nanotyrannosaur premaxillary tooth closely resembles that of the adults. It has the characteristic tyrannosauroid D-shaped cross-section with both carinae lying on the posterior surface of the crown (Figure 7a–f). The tooth differs from *T*. *rex* and *Tarbosaurus* but resembles *Nanotyrannus* in having a wide, blunt, chisel-shaped apex [46,64]. The crown is broad in lingual view, with mesial and distal carinae diverging distally then converging again towards the apex, giving the crown a spoon-like shape.

Carinae are unserrated; crowns of adult specimens referred to *Nanotyrannus* lack serrations on their mesial carina; the distal carina is unserrated apically and bears only a few serrations basally [46]. In mesial/medial or distal/lateral view, the crown is very straight, whereas the apex of the crown of tyrannosaurines is recurved in premaxillary teeth.

Juvenile/hatchling teeth perfectly matching this morphology have previously been reported [14] as *Aublysodon mirandus*, and are here reinterpreted as Nanotyrannidae.

### 3.2. Histology, Ontogenetic Stage, and Locomotion

Histology of the perinate *T*. *rex* (RSKM P2416.82) and *G. libratus* (TMP 1981.16.475) metatarsals suggests that they represent young hatchlings (<1 yrs of age) rather than embryos.

**Figure 8:**
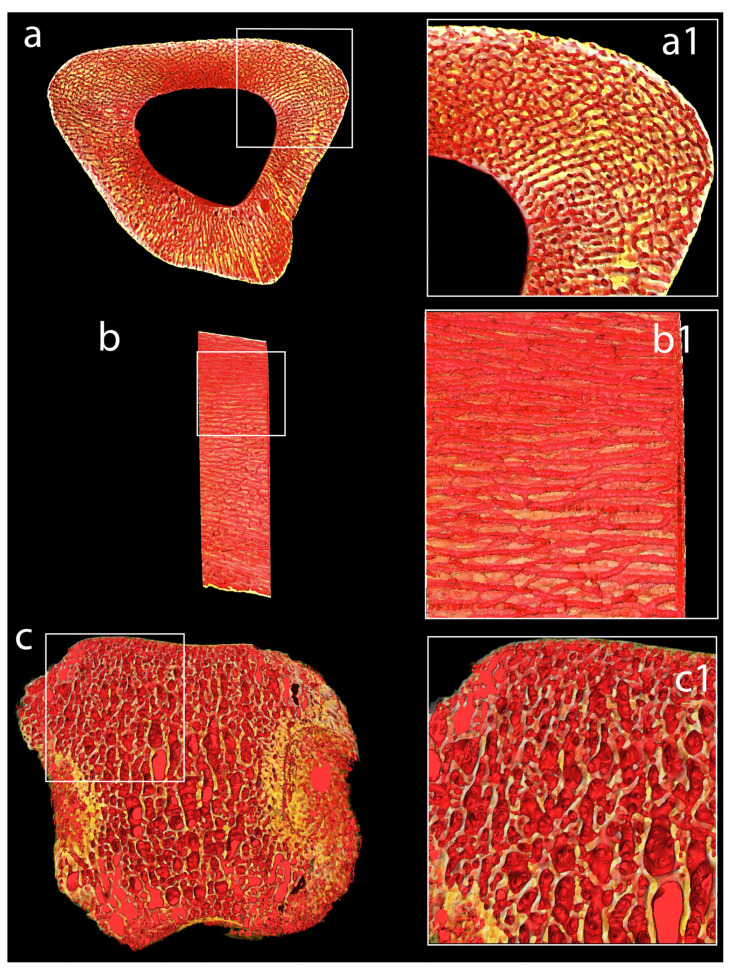
Synchrotron computed microtomography of RSKM P2416.82, left metatarsal III of *T*. *rex*, showing internal architecture. (**a**) Section through metatarsal midshaft in transverse plane, (**a1**) closeup showing extensive vascularization, (**b**) midshaft section rotated to show longitudinal canals, in sagittal plane (**b1**) closeup of longitudinal canals, (**c**) transverse section through articular surface showing well-developed cancellous structure, and (**c1**) closeup. The central image is rotated to show circumferential canals.

**Figure 9:**
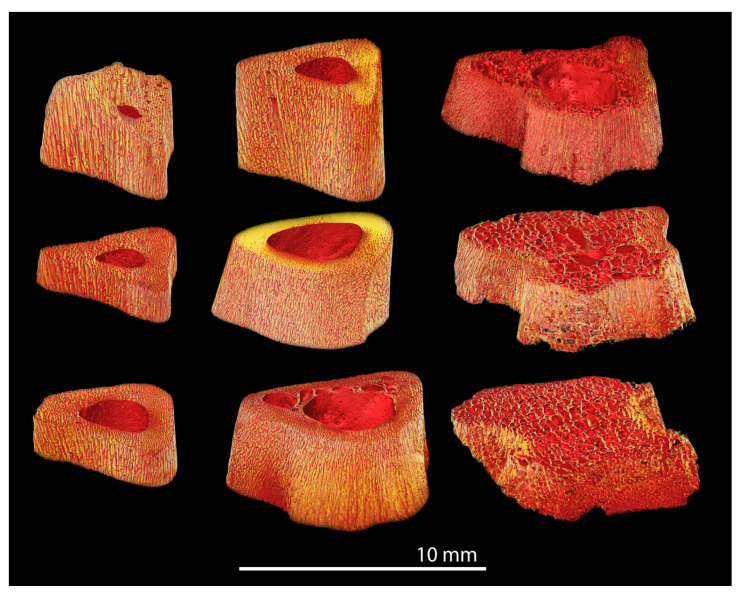
Synchrotron computed microtomography of *T*. *rex* RSKM P2416.82, left metatarsal III, serial sagittal sections from proximal (**top left**) to distal (**bottom right**) showing highly vascularized bone characteristic of very young, rapidly growing dinosaurs.

**Figure 10:**
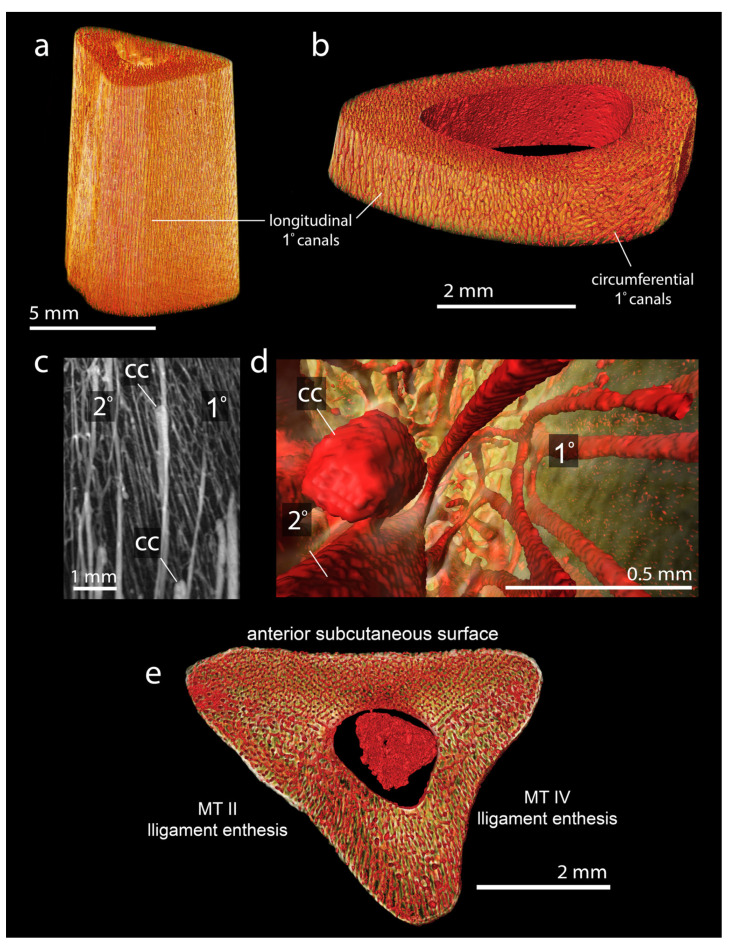
Vasculature in perinate tyrannosaurid left MT III of (**a**) *Gorgosaurus libratus* TMP 1981.16.475 and (**b**–**e**) *Tyrannosaurus rex* RSKM P2416.82. (**a**,**b**) Longitudinal and avian-like circumferential primary canals in (**a**) anterior oblique (*Gorgosauru*s) and (**b**) posterior oblique (*Tyrannosaurus*) section views; (**c**) cutting cones and broad secondary remodeling canals; (**d**) proximodistally oriented cutting cone, secondary remodeling canals, and primary osteons; (**e**) proximal *T. rex* MT III in transverse section.

**Figure 11:**
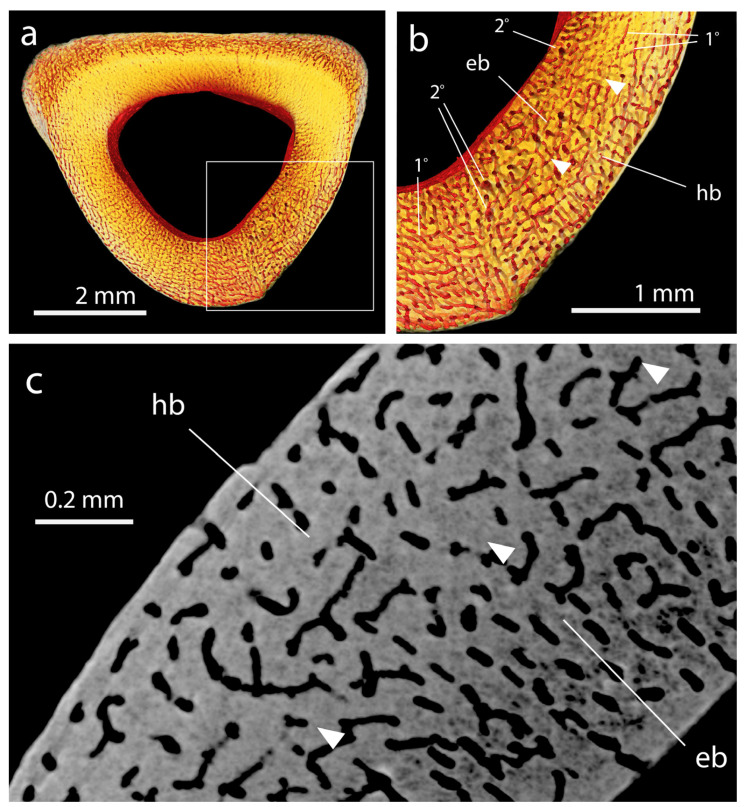
Histology of perinate *T*. *rex*. (**a**–**c**) *T. rex* RSKM P2416.82, showing transition between embryonic and hatchling bone. (**a**) Synchrotron scan volume rendering showing mid-shaft cortex and medullary space; (**b**) closeup showing transition from spongy embryonic bone to denser outer bone (arrows), possibly representing hatching line; (**c**) four stacked slices showing a likely transition from low-density embryonic bone to dense post-hatching bone and inferred position of embryo-hatchling transition (arrows). Abbreviations: eb, embryonic bone, hb, hatchling bone.

**Figure 12:**
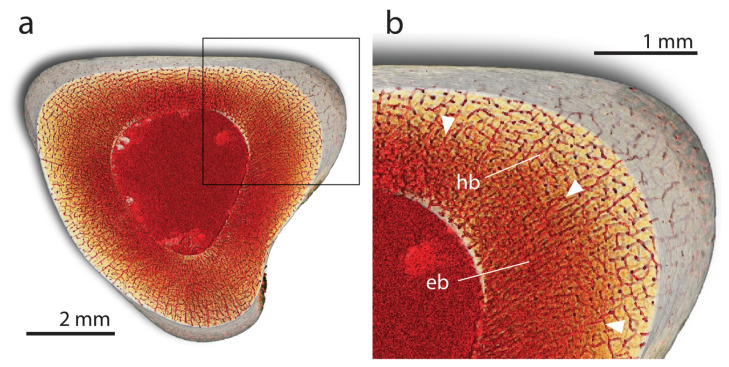
Histology of perinate *G. libratus* 1981.16.475, showing transition between embryonic and hatchling bone. (**a**) Cross-section; (**b**) closeup showing position transition between porous, highly vascularized embryonic bone and denser post-hatching bone (approximate position indicated by arrows). Abbreviations: eb, embryonic bone, hb, hatchling bone.

*LAGs*. First, lines of arrested growth (LAGs) are absent in the cortices of both the *T*. *rex* (Figure 8, Figure 9, Figure 10 and Figure 11) and *G. libratus* (Figure 12) metatarsals, whereas in older juvenile tyrannosaurid metatarsals, concentric vascular zonation is seen (Figure S11), and two or three LAGs are visible [7,65]. Lack of zonation and the high density of canals in hatchling bones contrasts with metatarsals of juvenile *T. rex* (age = ~3 years; Figure S11) and adult *G. libratus* (age = ~22 years [60]); e.g., bone from an adult *G. libratus* (Figure S14) is heavily remodeled with large-diameter cross-running secondary canals, and primary canals are absent; remodeling is evident in ground thin sections from the same bone [66]. The histology and extremely small size of the bones therefore indicate that they can only be hatchlings or embryos (i.e., between 0 and <1 years of age).

Synchrotron tomography furthermore reveals that the inner cortices of neonate *T*. *rex* and *G. libratus* are largely composed of primary bone and are replete with primary vascular canals (Figure 8, Figure 9 and Figure 10) seen as primary osteons and connecting canals (Volkman’s canals). Primary canals form a dense network running longitudinally along the metatarsals’ long axis (Figure 10). These features are also seen in cortices, periosteum and condyles under light microscopy. This high vascularization, common in woven bone, characterizes embryonic dinosaurs [67,68,69,70] and birds [69], being formed in the egg before hatching, and is retained after hatching in neonates [19,69].

The outer cortices of the metatarsals are relatively densely mineralized but contrast with open latticework of mid- and late-stage avian, troodontid, and ornithischian embryos [69]. This dense outer cortex is consistent with the post-hatching condition and inconsistent with embryonic bone.

In extant birds [71,72], crocodilians [73], squamates [74,75], and mammals [76], a distinct hatching line or (in viviparous taxa) a neonatal line characterizes the transition from embryonic bone to the bone of hatchlings or neonates. The hatching or neonatal line reflects changes in growth and loading following hatching or birth. A similar hatching line has been reported in dinosaurs [19,77], fossil rhynchocephalians and stem mammals [78], suggesting it is a conserved feature of amniotes.

Given its broad phylogenetic and temporal distribution and the dramatic shift in loading and growth that characterizes the embryo-neonate transition, such a transition in histology would be expected to be primitive and widespread in amniotes. The hatching line is narrow, and the associated histological cues are subtle; even using synchrotron scanning, we could not precisely and confidently identify a discrete hatching line. Instead, gradual changes in cortical bone structure (Figure 11 and Figure 12) are hypothesized to correspond to the embryo–hatchling transition, following Wolff’s Law [16,17], which observes that bone thickness and density respond to mechanical loads.

**Figure 13:**
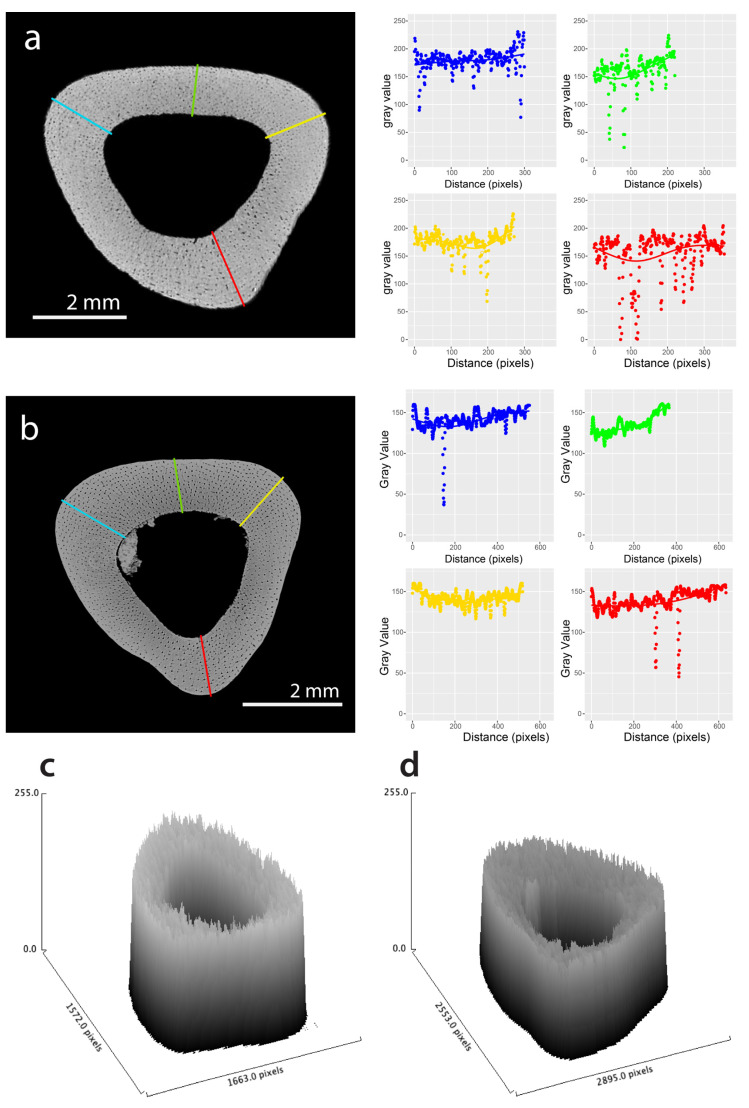
Bone density profiles of perinate tyrannosaurids. Bone density profiles for (**a**) *T. rex* RSKM P2416.82; (**b**) *G. libratus* TMP 1981.16.475, using gray value as a proxy for density; surface maps of CT slice data for (**c**) *T. rex* RSKM P2416.82; (**d**) *G. libratus* TMP 1981.16.475.

In the *T*. *rex* and *G. libratus* metatarsals, there is a marked change in patterns of cortical bone structure and density through the cortex (Figure 11 and Figure 12). The *T. rex* metatarsal is composed mostly of woven bone, with a thinner peripheral layer being formed of more compact bone (Figure 9 and Figure 11). Density is also low in the interior of the cortex, then begins to increase towards the periosteum, with density showing an inflection point about 25% of the distance from the inner cortex to the outer surface of the bone (Figure 11 and Figure 13). This density increase is seen in multiple transects of a single slice and in different slices, indicating it is a real feature rather than an artifact.

A similar change in bone structure density is seen in the *G. libratus* metatarsal (Figure 12 and Figure 13). Again, the inner cortex is more open, and the outer cortex is more compact; the cortex shows a region of relatively low density, which is followed by a concentric region of increasing density. Again, this feature occurs in multiple transects and multiple slices. Here, density begins to increase around 50% of the distance between the inner cortex and the outer surface of the bone.

These changes in bone architecture and density are hypothesized to correspond to the transition between embryonic bone and bone deposited in the days and weeks following hatching. The open, low-density inner cortex is interpreted as embryonic bone, which is subjected to limited mechanical loads in ovo. The zone of increasing bone density appears to reflect changes in bone density resulting from loading and perhaps also from changes in bone growth following hatching. These patterns occur in both *G. libratus* and *T*. *rex* hatchlings, again suggesting they are not an artifact. The density shift also appears at roughly the same place in the cortex in each specimen, although nearer the periosteum in *T. rex* but deeper in *G. libratus*. Similar changes in bone density are seen in hatchling titanosaur bones [19], with the shift in density coinciding with the inferred hatching line (Supplementary Materials), although the transition is more marked in tyrannosaurs. This suggests that this increase in bone density seen in these perinate metatarsals can act as an indicator of the embryo–hatchling transition.

The hypothesized hatchling bone layer is relatively thick, about 25% of the width of the cortex in *T*. *rex* and 50% of the width of the cortex in *G. libratus*. This suggests that both animals survived for some weeks or months after emerging from the egg. The thicker layer of dense bone in the *G. libratus* individual suggests it survived longer.

Another piece of evidence for the hatchling status of the bone is the presence of a relatively dense, weakly vascularized to avascular layer of bone along the inside of the medullary cavity in both the *T*. *rex* and the *G. libratus* metatarsal, which is particularly well-developed along the ventral part of the medullary canal. This layer appears to represent endosteal bone deposition, which typically takes place after hatching, and again supports a hatchling rather than embryo status for the material described here.

Further evidence for the hatchling status of the *T*. *rex* comes from the presence of secondary canals and cutting cones attesting to Haversian remodeling activity. One of the major functions of Haversian remodeling is to repair fatigue damage incurred during loading [79]. Osteoclasts tunnel through the bone, resorbing damaged primary bone tissue at the surface of the cutting cone. Subsequently, osteoblasts deposit new, secondary bone lamellae filling in the tunnels, save a Haversian canal in the center housing blood vessels. Haversian remodeling has not been reported for dinosaur [69] or bird embryos [72] but is seen in neonate dinosaurs [19]. The presence of this tissue in the *T. rex* specimen suggests it experienced increased bone strain magnitude and frequency necessitating repair owing to post-hatching motility.

Despite its small size, the *T*. *rex* metatarsal shows this Haversian remodeling (Figure 10), consistent with locomotion. Cutting cones of remodeling canals, which form the osteoclastic front of Haversian-style secondary osteons [80], run proximodistally, some originating near the phalangeal articulation (Figure 10) and others originating more proximal and superficial in the shaft. They are indistinguishable from remodeling canals of post-natal humans and rodents [80,81]. Similarly, canals in the perinate tyrannosaurs may reflect localized bending loads on the shaft [80], ground reaction forces and tension from ligaments concentrating compressive stresses [82], and load-induced fatigue fractures on the articular surface.

Although in ovo motions [83] could conceivably induce limb bone remodeling, the low strain magnitudes and frequencies resulting from in ovo movements seem unlikely to account for the remodeling seen here. Overall, these features are most consistent with osteological modification of the bones resulting from locomotion, as expected in precocial hatchlings and neonates. Finally, the relatively well-developed articular surfaces of the bone are highly suggestive of locomotion.

## 4. Discussion

### 4.1. Hatchling Size, Egg Size, and Clutch Size Estimation

The fossils described here allow us to constrain tyrannosaurid hatchling size. The tyrannosaurid hatchlings described here weighed 2.5 kg or less and measured ~700 mm in length at the time of death. They may have been even smaller when they emerged from the egg (Table 1), weighing about 1.7 kg in the case of *T*. *rex* and 1.3 kg in the case of *G*. *libratus*. This is smaller than previous reconstructions [13], which are based on the misattribution of juvenile ornithomimid remains to Tyrannosauridae (Supplementary Materials).

However, these estimates broadly agree with a small embryonic tyrannosaurid [13] (cf. Albertosaurinae) represented by a dentary. Although this fossil was previously identified as a young embryo, the well-developed tooth roots and replacement teeth and well-developed bone [13] suggest this specimen is a late embryo or even a hatchling. Comparisons with embryonic alligator [84] show that the bones of the skull develop and the teeth project up above the jaw margin very late in ontogeny. The reconstructed length of this specimen, 710 mm, agrees with the reconstructed hatchling sizes presented here; therefore, three different tyrannosaur specimens representing at least two, and perhaps three, distinct species (*T. rex*, *G. libratus*, cf. Albertosaurinae) independently yield broadly similar estimates of hatchling size.

This new information on hatchling size has important implications for understanding tyrannosaur reproduction and life history. Using the constraints provided by the specimens described here, it is possible to use the relationships between hatchling, egg, and clutch sizes to estimate tyrannosaurid reproductive parameters.

**Figure 14:**
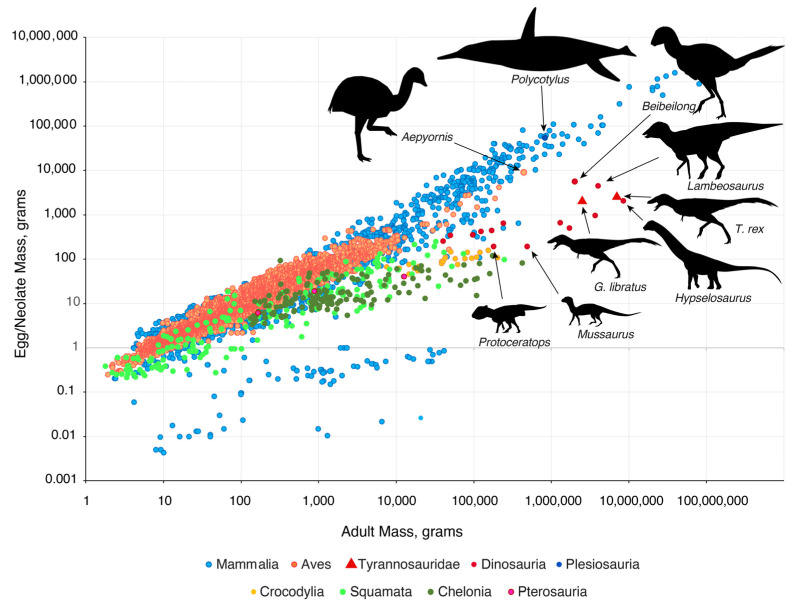
Egg size of tyrannosaurids and other tetrapods. Relative size of eggs/young of dinosaurs, birds, reptiles, and mammals; egg size for Dinosauria, extant and extinct, and inferred egg size for *T. rex*.

**Figure 15:**
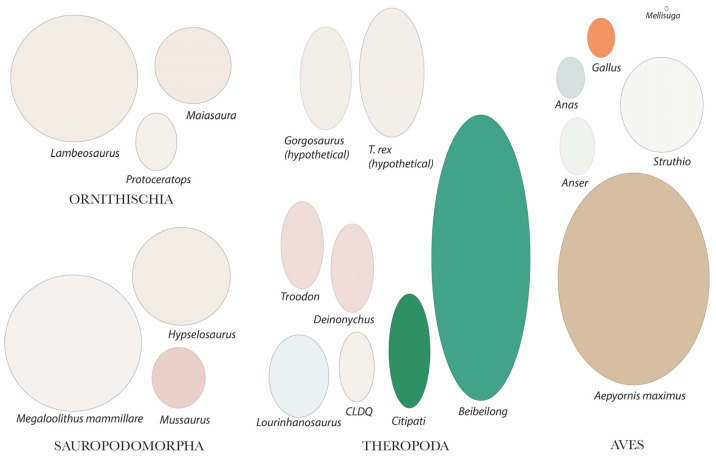
Egg size for Dinosauria, extant and extinct, and inferred egg size for *T. rex*.

Tyrannosaurid hatchlings, and therefore eggs, are relatively larger (Figure 14) than in squamates, turtles, crocodilians, pterosaurs, or non-avian Dinosauria such as protoceratopsids and basal sauropodomorphs [85]. Tyrannosaurid eggs are comparable in absolute and relative size to those in Saurolophinae and Titanosauria (Figure 15) but relatively smaller than in other dinosaurs, including Lambeosaurinae, Eumaniraptora, and crown Aves. Tyrannosaur eggs are absolutely smaller than the largest non-avian dinosaur eggs known, which come from giant oviraptorosaurs. Remarkably, *T. rex* eggs would have weighed less than eggs of the largest modern birds, the elephant bird *Aepyornis maximus* or the moa *Dinornis robustus*, even though an adult *T*. *rex* was more than an order of magnitude more massive.

**Figure 16:**
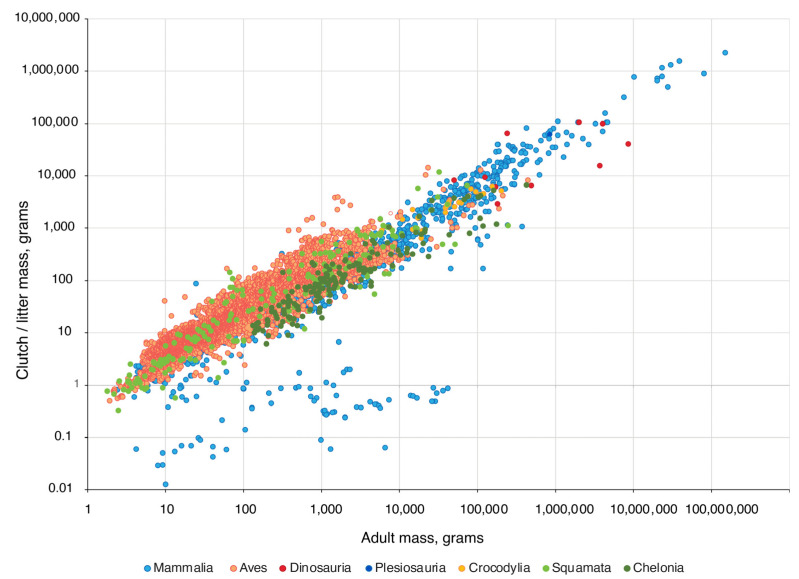
Clutch/litter mass versus body mass for dinosaurs and other amniotes. Amniotes show a strong positive correlation between clutch/litter mass and body mass. Marsupials form the cloud below the other mammals.

Given the tradeoff between egg/offspring size and clutch number, tyrannosaurs (and dinosaurs in general) likely laid fewer eggs than predicted for comparable-sized crocodilians and turtles, but more than predicted for similar-sized birds. Since clutch mass correlates strongly with body mass (Figure 16), we can approximately estimate *T. rex* clutch mass (Table S6).

The most conservative clutch mass estimates (Table 2) suggest a 54 kg or 60 kg clutch in MOR 1125, a 6100 kg [21], reproductively mature female *T. rex* [22], depending on whether a crocodilian OLS regression or avian PGLS regression model is used. Estimates using these models are 71 kg and 79 kg in FMNH PR 2081, a 9500 kg [21] *T*. *rex* (Table 2) which represents one of the largest known *T. rex* specimens. It remains unknown whether this individual is male or female, and so it is possible that as in many birds, the largest female *T*. *rex* were somewhat smaller than males, in which case maximum clutch mass was correspondingly smaller.

**Figure 17:**
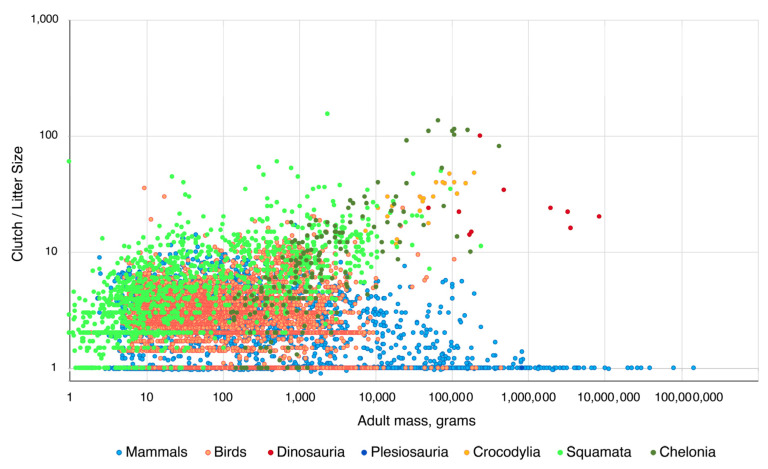
Clutch/litter size versus body mass for dinosaurs and other amniotes. Squamates, turtles, and crocodilians show increasing clutch size at large size. Non-avian dinosaurs show high variability and no clear trend between clutch size and body size. Mammals and crown birds tend to show smaller clutch size at larger body size.

These clutch mass estimates in turn imply clutch sizes of 21–32 eggs (Table 3), more than most birds, but fewer than in large turtles and crocodylians (Figure 17). These clutch sizes overlap with those of crocodilians, but given the positive relationship between clutch size and body size in crocodilians (Figure 17), a *T*. *rex*-sized crocodilian should have a larger clutch size than *T*. *rex*.

Other models suggest much larger clutch masses and correspondingly larger clutch sizes. An OLS model for all diapsids (Table 2) suggests clutch masses of 120 kg and 169 kg for a 6100 and 9500 *T*. *rex*, respectively, suggesting clutch sizes of approximately 50–70 eggs (Table 3). An avian OLS model suggests clutch masses of 190 kg and 272 kg, respectively, implying clutch sizes of approximately 80–110 eggs (Table 3). Although these are very large clutch masses, at least two non-avian dinosaur nests are known with estimated clutch masses of ~100 kg (Supplementary Materials). One nest was laid by a giant oviraptorosaur, which are known to have reached a size of 2000 kg, far less than an adult *T*. *rex*. The other was laid by the hadrosaurid *Hypacrosaurus*, with a mass of 4000 kg, again less than the mass of an adult *T*. *rex*. A clutch mass on the order of 100–300 kg for a large, 9–10 tonne *T*. *rex* therefore is not biologically impossible or even implausible. This would only represent around 1–3% of the body mass, within the range seen in many birds and dinosaurs.

What we can therefore say with some confidence is that tyrannosaurs probably laid at least 15–30 eggs. Larger clutches of 50 or even 100 eggs are not impossible. None of our models support the idea that tyrannosaurs had small clutches.

These numbers can create a sense of false precision: we emphasize that these are not intended to be *precise* estimates of reproductive parameters but rather approximations, i.e., an order-of-magnitude problem or Fermi Problem approach. Furthermore, they estimate species means. The size of any given *T*. *rex* clutch must have varied from individual to individual, and from year to year, as it does in extant birds.

Furthermore, because in non-avian dinosaurs, reproductive maturity began before reaching full size [86], reproductive output likely varied throughout life. In crocodilians [87] and squamates [88,89], both egg mass and clutch size increase in larger females. It is conceivable that the same was true of tyrannosaurs and other non-avian dinosaurs.

Nevertheless, while these estimates are not meant to be precise, the Fermi approach tends to produce surprisingly accurate results even when many parameters are estimated. This is because errors in multiple parameter estimates tend to cancel out, so long as there is no bias in a particular direction. Furthermore, as shown here, these findings are robust against different assumptions; i.e., even using different estimates for offspring size, egg size, and clutch size, and even accounting for uncertainty in our estimates, all estimates broadly agree that tyrannosaurids had relatively small eggs and offspring (Table S6) and that these in turn imply relatively large clutch sizes compared to modern birds or even non-avian Maniraptora such as Dromaeosauridae, Troodontidae and Oviraptorosauria. It is difficult to use a plausible set of estimated parameters and arrive at a calculation showing large offspring and small clutches for *T*. *rex* (Table 3).

### 4.2. Parental Care in Tyrannosauridae

Large clutches suggest that tyrannosaurs employed an *r*-selected reproductive strategy, emphasizing high reproductive rate while limiting parental investment in individual offspring. Generally speaking, animals with large clutches, such as lizards and turtles, tend to provide limited or no parental care (Figure 17), as the sheer number of offspring limits the ability to devote attention or invest heavily in any single offspring. Still, tyrannosaurs likely provided a degree of parental care for their hatchlings.

Turtles and nearly all squamates [90] provide minimal parental care and typically abandon eggs after laying. However, crocodilians guard nests and hatchlings [91], as do extant Dinosauria (Aves). Phylogenetic bracketing [92] implies these behaviors are ancestral for Archosauria and Dinosauria. This conclusion is corroborated by the discoveries of brooding oviraptorosaurs [93,94] and troodontids [95] and also juvenile–adult associations in *Psittacosaurus* [96], suggesting parental care was widespread in non-avian Dinosauria.

Yet parental care may have been less intensive among dinosaurs than in modern birds. In contrast to many modern birds, parental care was likely uniparental rather than biparental [97]. Given that parental care is uniparental in crocodilians and many basally diverging bird groups, including most ratites and galloanserines, uniparental care likely represents the ancestral condition. Furthermore, among birds, intensive parental care, where young are nestbound and dependent on parental feeding, is restricted to Neoaves; Galloanseres and Palaeognathae resemble crocodilians in having precocial, self-feeding offspring [98]. They may sometimes feed their young, but chicks still are able to forage for themselves [98].

Given the more limited, primitive forms of parental care and the high degree of precociality seen in crocodilians and basally diverging birds, the phylogenetic bracket implies that hatchling tyrannosaurs and most non-avian dinosaurs resembled early-diverging birds in being precocial and self-feeding, although altriciality has been proposed for some species [99].

The Haversian remodeling seen in even the smallest known tyrannosaurids and well-developed joint surfaces also suggest that tyrannosaurs lacked a nestbound phase. They apparently left the nest soon after hatching and likely hunted as well. However, while juvenile crocodilians feed on invertebrates and small vertebrates, the tooth wear seen in hatchling tyrannosaurids suggests they ate relatively large vertebrates.

### 4.3. Reproductive Strategy in Tyrannosaurs

It is also possible to use the relative size of hatchlings and inferred clutch sizes to make general inferences about reproductive strategies. Differences between species in offspring size and offspring number reflect distinct reproductive strategies, often characterized as *r*-selection versus *K*-selection [100].

Organisms can either maximize offspring number, or per-offspring investment. However, given finite energy and resources, they cannot do both. A tradeoff between offspring number and offspring size necessarily emerges.

Large clutch sizes increase reproductive rate (*r*) but limit investment per offspring, so r-selection tends to be favored when high levels of predation or harsh environments reduce juvenile survival [101]. Investing heavily in few offspring limits *r* but increases individual offspring fitness. This strategy may be favored if juvenile mortality is relatively low and environments are near carrying capacity (*K*), creating intraspecific competition [101].

Compared to Aves, tyrannosaurids and other non-avian dinosaurs typically pursued an *r*-selected strategy, laying relatively smaller, more numerous eggs [102]. Yet non-avian dinosaurs appear *K*-selected relative to turtles, squamates, and crocodilians, laying larger and fewer eggs (Figure 14); their reproductive strategies appear intermediate between those of extant birds and mammals and diapsid outgroups. Some clades, however (Protoceratopsidae, basal Sauropodomorpha, pterosaurian outgroup), plesiomorphically retain small eggs (Figure 14), suggesting more crocodilian-like reproductive strategies.

These relatively large clutches suggest neonate survival was low for tyrannosaurids and other non-avian dinosaurs, as seen in other carnivores, and as previously proposed for tyrannosaurids [65]. Predation by theropods likely increased mortality. Predation likely included cannibalism, given evidence for such behavior [5] in tyrannosaurids and other theropods [103] and given that cannibalism is common in extant large predators such as alligators [104], bears [105], and lions [106].

**Figure 18:**
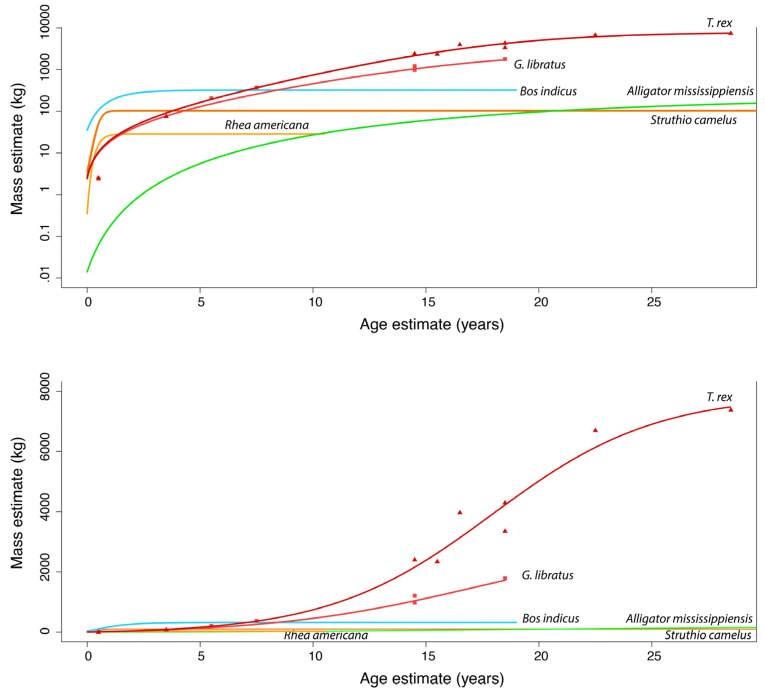
Growth curves (kg versus years of age) for *T. rex*, *G. libratus*, a mammal (*Bos indicus*), ratites (*Rhea americana*, *Struthio camelus*), and a crocodilian (*Alligator mississippiensis*) plotted on a log axis (**top**) and a non-log axis (**below**). The slope of the data plotted with a logarithmic Y-axis reflectsrelative growth, and demonstrates an earlier asymptote of growth for extant ratites and mammals versus longer-term growth in the tyrannosaurs and *Alligator*. (Specimens whose taxonomic status remains contested have been excluded). Data sources: Data for *Rhea* from Navarro et al. [107]; for *Struthio* from Ramos et al. [108]; for *Alligator* from Wilkinson and Rhodes [109] using Farlow et al. [110] to convert length to mass; for cattle from Bahashwan et al. [111]; and for Asian elephant from Mumby et al. [112].

Parental care may also have been less intensive in tyrannosaurids and non-avian dinosaurs, or simply less effective at reducing mortality, because young dinosaurs grew more slowly than birds (Figure 18). The rapid growth seen in birds such as ostriches means that young rapidly reduce vulnerability to predators by minimizing the period during which they are small and at highest risk of predation. By the time young ostriches fledge, they are large enough to fend for themselves. Dinosaurs grew more slowly; assuming tyrannosaurs received parental care for a year, similar to modern ratites, then tyrannosaurs would have achieved full independence at small size (Figure 18), leaving them vulnerable to predators upon independence.

### 4.4. Parental Care Evolution

With small offspring and large clutches, it seems unlikely that non-avian dinosaurs, especially tyrannosaurs, invested heavily in offspring. Nevertheless, dinosaur reproduction shows a major shift in reproductive strategies during the Mesozoic. While tyrannosaurids had small offspring relative to extant birds and mammals, they had larger offspring than early Dinosauria and non-dinosaurian diapsids (Figure 14) and likely fewer offspring relative to body mass. Dinosaur egg mass varied, but both relative and absolute egg size increased in the Mesozoic.

Given the existence of small eggs in both basal sauropodomorphs and ceratopsians, it is possible that large eggs evolved independently in Sauropoda, Ornithopoda, and Theropoda. The evolution of large eggs in Theropoda was followed by the evolution of yet larger eggs in Maniraptora and even larger eggs in crown Aves. The evolution of larger eggs may have driven the evolution of calcification of the egg shell [85] to help support these larger eggs.

It is noteworthy that shifts towards intensive parental investment and *K*-selected reproductive strategies, and away from ancestral *r*-selected strategies, did not just evolve in dinosaurs during the Mesozoic, but appeared in many other clades, taking diverse forms. Several clades of marine reptiles, including Ichthyosauria [113], Sauropterygia [114,115], and Mosasauroidea [116], evolved either ovoviviparity or viviparity. Egg retention reduces mortality at the embryo stage, letting these lineages pursue a *K*-selected strategy of investing heavily in relatively few, large young, without necessarily feeding or protecting them following hatching/birth. Groups of sharks characterized by live birth and *r*-selected reproductive strategies, such as Lamnidae [117], also appeared. Stem mammals evolved parental care [118], and tooth replacement patterns correlated with lactation [119] suggest the appearance of parental feeding. Therian mammals, characterized by viviparity, the production of milk, and young dependent on parental care, appeared [119,120]. Social insects, including termites, ants, wasps, and bees, all characterized by intensive parental care and young or larvae dependent on parental care, evolved and diversified in the Jurassic and Cretaceous [121]. Among plants, increased investment in propagules and *K*-selected reproductive strategies are seen in the appearance of larger seeds in the Cretaceous [122].

The shift towards more *K*-selected strategies seen in dinosaurs may therefore be part of a larger pattern of increased parental investment in the Mesozoic. Following extinction of dinosaurs across the K-Pg boundary, these trends continued as mammals [123] and birds [124] radiated and evolved intensive parental investment strategies, such as biparental care and feeding of chicks in Neoaves. These patterns raise the possibility that increased offspring investment in coelurosaurs like *T. rex* and other dinosaurs may be part of a long-term macroevolutionary trend towards increased parental investment in fewer offspring.

## 5. Conclusions

Bones and teeth of small tyrannosaurs represent hatchlings and young juveniles of *T. rex* and *G. libratus*. They show that tyrannosaur eggs and hatchlings were small, <2.5 kg, and suggest that young were highly precocial, able to move and feed themselves soon after hatching. Given that tyrannosaurs could produce large clutch masses, tyrannosaurs likely had large numbers of offspring. This suggests a more *r*-selected reproductive strategy than seen in modern birds. The reproduction of tyrannosaurs and many other non-avian dinosaurs was likely intermediate between the highly *r*-selected strategy of primitive diapsids such as turtles and crocodilians, which have very large numbers of small offspring and limited parental care, and extant birds, which use *K*-selected strategies with larger eggs and hatchlings but fewer of them. Dinosaur reproduction evolved over the course of the Mesozoic, with parental investment becoming more intensive.

## Figures and Tables

**Table 1:** Hatchling Mass Estimates and Egg Mass Estimates.

Species	Specimen	Model	Hatchling Mass Estimate, g	Egg Mass (Crocodilian Model), g	Egg Mass (Avian Model), g
*T. rex*	RSKM P2416.82	OLS, unadjusted	2459	3783	3659
		OLS, adjusted for growth	1668	2566	2482
*G*. *libratus*	TMP 1981.16.475	OLS, unadjusted	2389	3675	3555
		OLS, adjusted for growth	1325	2038	1972

**Table 2:** Clutch Mass Estimates using Diapsid, Crocodylian and Avian linear regression models.

Species	Specimen	Adult Body Mass, g	Clutch Mass, Diapsid OLS	Clutch Mass, Crocodylian OLS	Clutch Mass, Aves PGLS	Clutch Mass, Aves OLS
*T*. *rex*	FMNH PR 2081	9500000	169281	71093	79301	271667
*T*. *rex*	MOR 1125	6100000	120264	54138	59558	189986
*G*. *libratus*	NMC 2120	2487000	59423	31178	33351	92079

**Table 3:** Clutch size estimates, made using egg mass estimates (Table 1) and clutch mass estimates (Table 2).

Species	Specimen	Clutch Mass Model	Unadjusted Egg Mass Estimate		Adjusted Egg Mass Estimate	
			Crocodilian Egg Mass Model	Avian Egg Mass Model	Crocodilian Egg Mass Model	Avian Egg Mass Model
*T*. *rex*	MOR 1125	Diapsid Clutch Mass Model (OLS)	32	33	47	48
		Crocodilian Clutch Mass model (OLS)	14	15	21	22
		Avian Clutch Mass Model (PGLS)	16	22	23	24
		Avian Clutch Mass Model (OLS)	50	52	74	77
*T*. *rex*	FMNH PR 2081	Diapsid Clutch Mass Model (OLS)	45	46	66	68
		Crocodilian Clutch Mass model (OLS)	19	19	28	29
		Avian Clutch Mass Model (PGLS)	21	22	31	32
		Avian Clutch Mass Model (OLS)	72	74	106	109
*G. libratus*	NMC 2120	Diapsid Clutch Mass Model (OLS)	16	17	29	30
		Crocodilian Clutch Mass model (OLS)	15	17	15	17
		Avian Clutch Mass Model (PGLS)	16	17	16	17
		Avian Clutch Mass Model (OLS)	25	26	45	47

## Data Availability

All data is in the paper or the Supplementary Materials.

## Supplementary Materials

The following supporting information can be downloaded at: https://www.mdpi.com/article/10.3390/biology15131090/s1, Supporting Information File S1; Supplementary Materials Data S1; Supplementary Materials Data S2. Refs. [7,13,18,19,20,21,22,23,24,25,26,32,34,35,36,37,38,39,42,43,44,45,46,50,51,54,61,110,112,125,126,127,128,129,130,131,132,133,134,135,136,137,138,139,140,141,142,143,144,145,146,147,148] are cited in Supplementary Materials.

## Abbreviations

The following abbreviations are used in this manuscript:

FMNH	Field Museum of Natural History, Chicago, Illinois, USA
MOR	Museum of the Rockies, Bozeman, Montana, USA
NMC	National Museum of Canada, Ottawa, Ontario, Canada
TMP	Royal Tyrrell Museum of Paleontology, Alberta, Canada, Canada
RSKM	Royal Saskatchewan Museum, Eastend, Saskatchewan, Canada
YPM	Yale Peabody Museum, New Haven, Connecticut, USA
