# Supplementary material for: Hatchlings of Tyrannosaurus rex and the Evolution of Dinosaur Reproductive Strategies
Source: Biology (Basel). 2026 Jul 7;15(13):1090. doi: 10.3390/biology15131090 (PMC13359715; doi:10.3390/biology15131090)
Supplement: Supplementary file 1 [file biology-15-01090-s001.zip › Supporting Information File S1.pdf]

ONLINE SUPPLEMENTARY INFORMATION

Hatchlings of *Tyrannosaurus rex* and the evolution of dinosaur reproductive strategies

Nicholas R. Longrich, Peter J. Makovicky, Tim Tokaryk, David M.L. Cooper, Evan T. Saitta, Gregory M. Erickson, Tamas Szekely, Eric Snively

Table of Contents

1. Diagnosis of Tyrannosaur Fossils
2. Mass Estimation
3. Growth Curves
4. Estimation of Reproductive Parameters
5. Synchrotron MicroCT Scanning and Reconstruction
6. Bone Density Profiles
7. Fossil Provenance
8. References

1. Diagnosis of Tyrannosaurid Fossils

Comparisons with other theropods show that the metatarsals described here do not match the metatarsals of other theropods from the Late Cretaceous of North America (Figure S1). Instead, they closely match the morphology of Tyrannosauridae (Figure S2).

Features supporting tyrannosaurid affinities include a metatarsal III that is pinched between metatarsals II and IV, a sigmoidal curvature of the metatarsal shaft; a deep depression proximal to the articular condyle on the dorsal surface of the metatarsal, a metatarsal articular surface that is subequal in width and height, ventral deflection of the articular surface and (in *T. rex*) strong ventral deflection of metatarsal III. Of 9 tyrannosaur characteristics identified, the metatarsals exhibit all 9 (Table S1) No derived characters were found supporting referral to any other theropod clade except those that are also found in Tyrannosauridae (e.g. pinched metatarsal III).

Although differences exist between the juvenile material referred to Tyrannosauridae here and adult Tyrannosauridae, most of these can be explained as the result of change in morphology with size. RSKM P2416.82 can be connected into an ontogenetic series that shows a continuous change in morphology with size between large and small Tyrannosauridae (Figure S2). Changes seen with increasing size in Tyrannosauridae include a wider MT III shaft, stronger twisting of the shaft, a broader condyle, and (in *Tyrannosaurus*) a wider ventral pillar.

Similarly, teeth exhibit characteristics of Tyrannosauridae. Premaxillary teeth exhibit a D-shaped cross-section with mesial carinae twisted onto the lingual surface of the crown and a strong lingual ridge running from the base of the crown to the apex. Lateral teeth exhibit a twisting of the carinae, with mesial carinae twisting onto the lingual surface of the crown towards the base, and distal carinae twisting onto the labial surface towards the base. Of dental characters for tyrannosaurs identified (Table 1) all are shared with some or all tyrannosaurs. Tooth wear (strong apical wear) is also typical of Tyrannosauridae. No characters were found to support an alternative placement of these teeth.

To test the affinities of the material described here, a character list was generated and the material was included in a character-taxon matrix. The matrix was run as part of a constrained phylogenetic analysis in PAUP* 4.0b10 [125] to test the affinities of the fossils described here. A backbone constraint tree was used to fix the position of Tyrannosauridae, Dromaeosauridae, Troodontidae, Oviraptorosauria, and Alvarezsauridae (see Figures S4, S5), with the isolated fossils free to assume any position in the tree. A heuristic search was run.

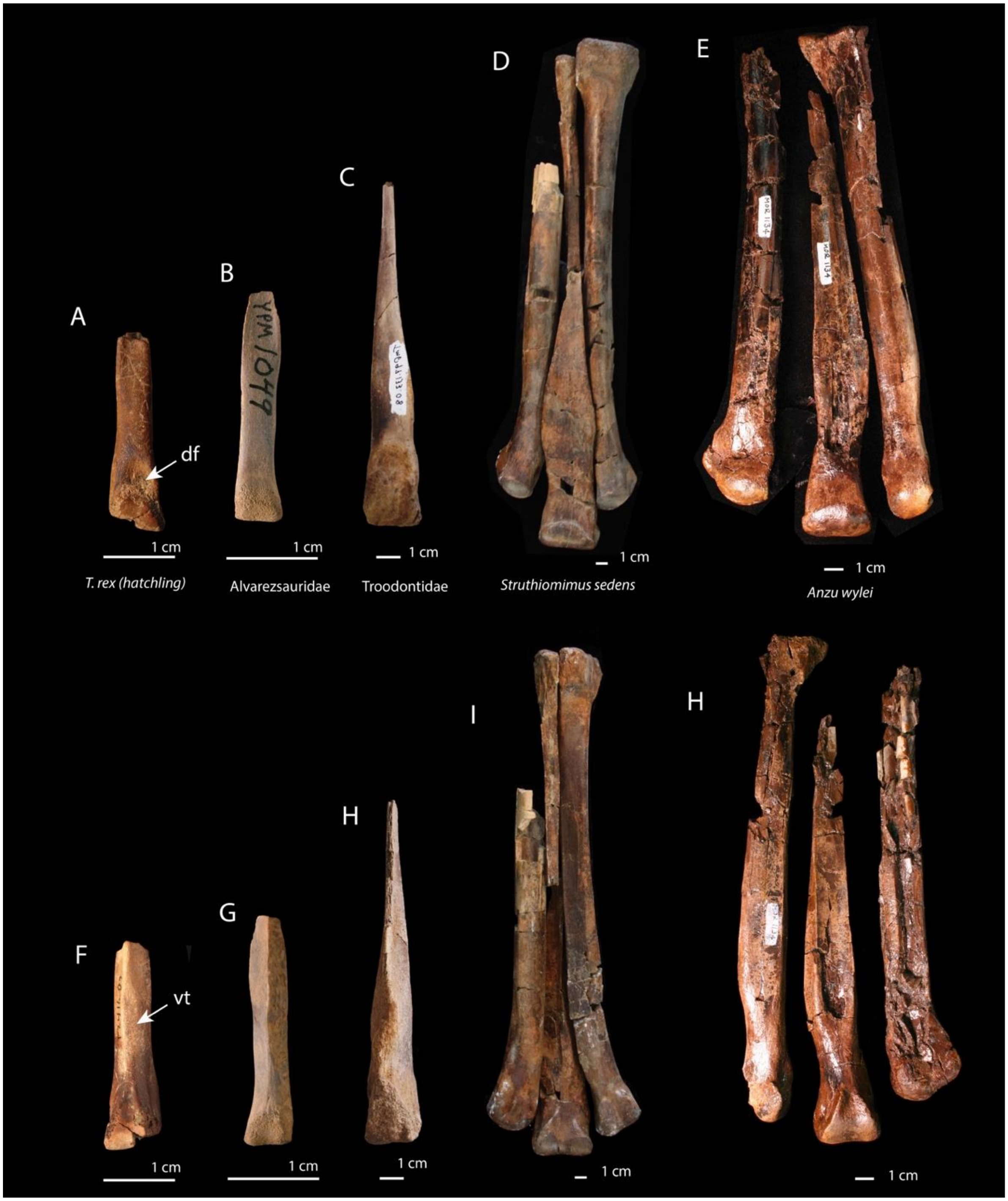

Figure S1. Metatarsal III of theropods from the Late Cretaceous of Laramidia. **A**,F hatchling *T. rex* RSKM P2416.82; **B**,**G** Alvarezsauridae (YPM 1049); **C**,**H** Troodontidae (Troodontidae indet. TMP 1997.133.08); **D**,**I** Ornithomimidae (*Struthiomimus sedens*, Royal Saskatchewan Museum); **E**,**H** Oviraptorosauria (cf. *Anzu wylei*, RSKM P2631.1). **E**, dorsal; **F-H**, ventral. Abbreviations: df, dorsal fossa; vt, ventral tubercle.

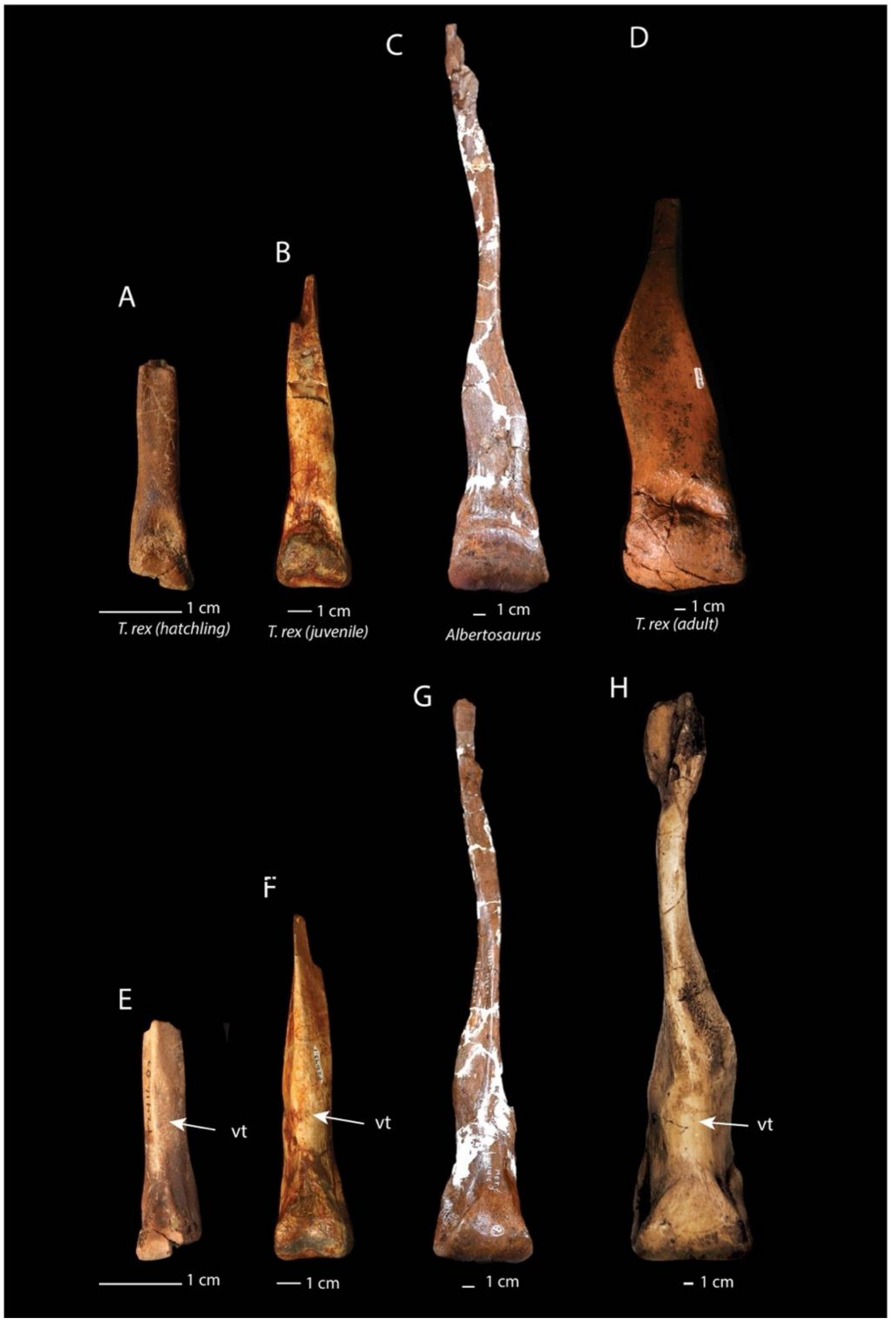

Figure S2. Size series of tyrannosaurid metatarsal III (scaled to the same estimated length). **A**, **E** hatchling *T. rex* RSKM P2416.82, **B**, **F** juvenile *T. rex* RSKM P2643.1; **C**, **G** *Albertosaurus sarcophagus* CMN 11315 (from Mallon et al., 2020), **D, H** adult *T. rex* (D1: MOR 1602; D2: LACM 7244-23744). **A-D**, dorsal; **A-H**, ventral. Allometric changes include expanded flanges of MT III and broader distal articular surface in larger animals. Abbreviation: vt, ventral tubercle.

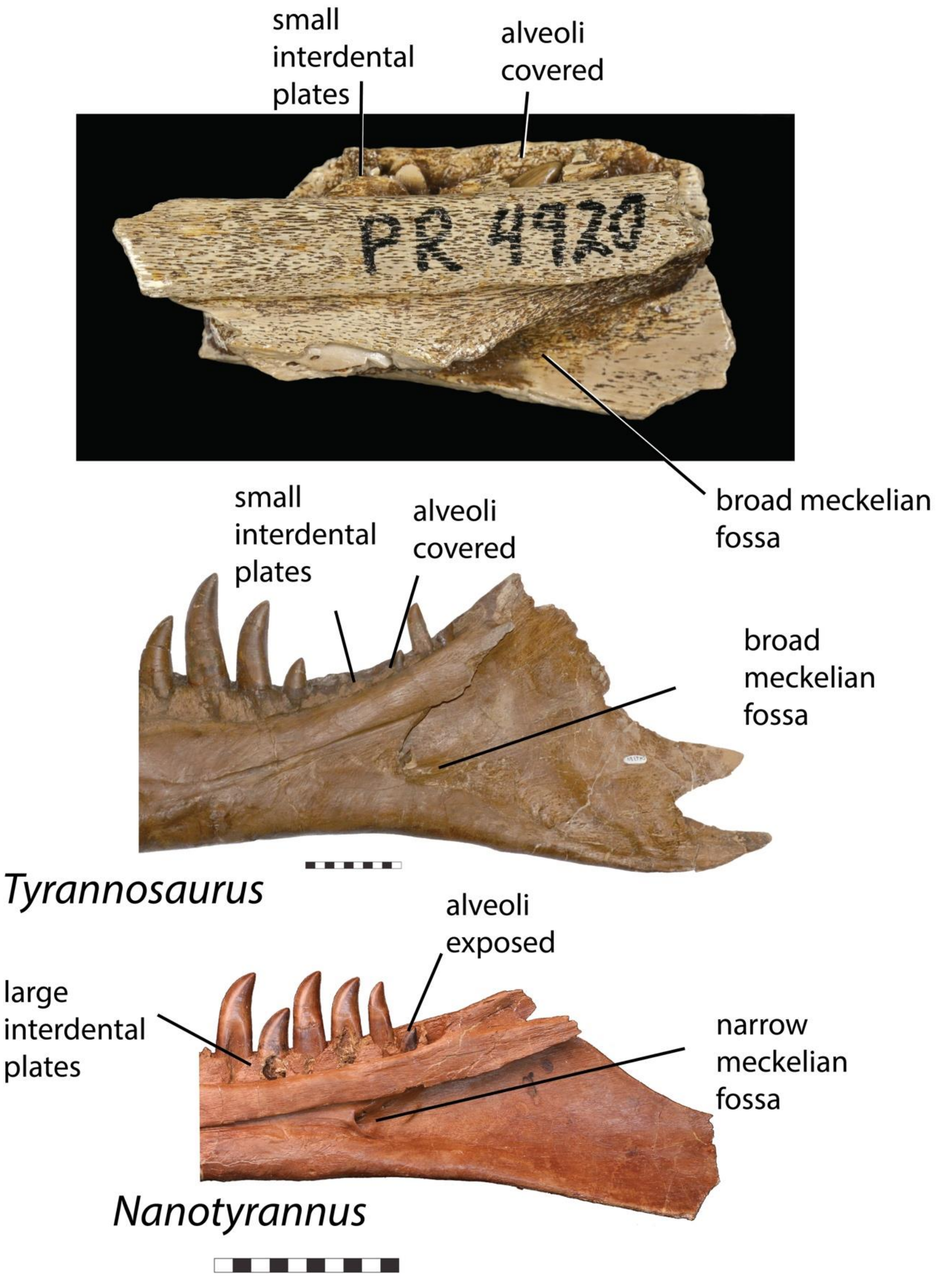

Figure S3. Comparison of *T. rex* and *Nanotyrannus* jaws. Features of FMNH PR 4920 allow referral to *Tyrannosaurus rex*.

Supplementary Table S1. Character List.

1. **Metatarsal III, shaft of metatarsal broadly exposed along entire length of metatarsus (0) or narrow, strongly pinched between metatarsals II and IV (1).** See Holtz [126]; Brusatte et al. [127]: Character 200.

   In RSKM P2416.82, metatarsal III has a triangular cross section with the shaft converging posteriorly to be constricted between metatarsals II and IV, a condition known as an arctometatarsus [126]. Distribution: Tyrannosauridae [34,39,44] (see also FMNH PR 2081, TMP 81.10.1), Troodontidae [42] (e.g. TMP 92.36.575), Ornithomimidae [35,128,129] (see also TMP 95.110.1), Alvarezsauridae [37] and Microraptorinae (Hwang et al., 2002); absent from Caenagnathidae [38,130] and Eudromaeosauria [131] (see also MOR 660; TMP 88.121.39).

2. **Metatarsal III, distal articular surface proportions: wider than tall (0), as wide as tall (1), or taller than wide (2). ORDERED.**

   In distal view, height and width of the distal articular surface are subequal in Tyrannosauridae [34,39], Caenagnathidae [43,130], and Alvarezsauridae (Longrich and Currie, 2009). The distal articular surface is wider than tall in Ornithomimidae [132]. It is taller than wide in Dromaeosauridae [45] (see also cf. *Saurornitholestes* MOR 660; TMP 88.121.39) and Troodontidae [42,133] (see also TMP 92.36.575).

3. **Metatarsal III, shape of distal end of shaft: straight in lateral view (0), deflected ventrally relative to proximal end in lateral view by 20º (1), or strongly deflected in lateral view by 40º (2). ORDERED.**

   NEW CHARACTER. Primitively in theropods such as dromaeosaurs, caenagnathids, and ornithomimids, the third metatarsal is relatively straight in lateral view. Tyrannosauridae are unique among North American theropods [34,39] in that the distal end of metatarsal III is deflected ventrally relative to the proximal end of the shaft, so that the shaft appears bent or crooked in lateral view. This character is weakly developed (State 1) in Albertosaurinae and *Daspletosaurus* cf. *torosus* (Yun, 2021), and more strongly developed (State 2) in *T. rex* (Figure S2). The distal section of the shaft of MT III is straight in *Albertonykus* [37], ornithomimids (TMP 95.110.1), eudromaeosaurids (MOR 660; TMP 88.121.39), and troodontids [42].

4. **Metatarsal III, anterior surface of shaft: extensor fossa shallow and indistinct (0), or deep, U-shaped extensor fossa proximal to distal articular surface of MT III, which slopes upwards proximomedially (1).**

   NEW CHARACTER. On the dorsal surface of metatarsal III, there lies a deep, asymmetrical, U-shaped depression or pit proximal to the distal articular surface in Tyrannosauridae [34,39,44].

The depression is poorly developed or indistinct in ornithomimids [132] and caenagnathids [38,134] and also shallow and indistinct in *Albertonykus* and other alvarezsaurids [37]. The extensor fossa is developed in Campanian eudromaeosaurids of North America (MOR 660; TMP 88.121.39; AMNH PR 30556 *Bambiraptor feinbergi*), but is bowl-shaped and lacks the distinct U-shape described here.

A similar depression is found in Troodontidae [42] among North American theropods, but in Troodontidae, the pit is taller, excluded from the medial 30% of the metatarsal's width, and lies on an autapomorphic flange that extends laterally towards MT IV.

5. **Metatarsal III, distal articulation, proximal margin of articular surface: straight or asymmetric in dorsal view (0) or strongly convex, U-shaped in dorsal view (1).**

   NEW CHARACTER. Tyrannosauridae, Alvarezsauridae, Caenagnathidae. In Ornithomimidae, the proximal margin is straight, e.g. *Ornithomimus velox* [36] to asymmetrical, e.g. *Struthiomimus altus* [128]. In Caenagnathidae [134], the proximal margin is gently convex, but less U-shaped than in tyrannosaurids such as *T. rex* (FMNH PR 2081).

6. **Metatarsal III, distal articular surface lacks strongly ginglymous joint (0) well-developed ginglymous joint (1).**

   See Brusatte et al. [127]: Character 199. The joint is weakly ginglymous in Ornithomimidae [36]. The surface bears a weak posterior groove in *Albertosaurus* [44], *Daspletosaurus* [39] and *Tyrannosaurus* [34] and also in Caenagnathidae [38] and Alvarezsauridae [37], but is otherwise convex. In Dromaeosauridae [131] (see also MOR 660; TMP 88.121.39; AMNH PR 30556 *Bambiraptor feinbergi*) and Troodontidae [42] metatarsal III has a strongly ginglymous articular surface, a derived condition.

7. **Metatarsal III, dorsal surface narrow, with weak expansion of shaft of metatarsal III (0) or wide and strongly expanded to dorsally overlie MT II and IV (1).**

   NEW CHARACTER. In Tyrannosauridae [34,44], Alvarezsauridae [37], Dromaeosauridae [45,131] and Troodontidae [42,133] the dorsal surface of metatarsal III is relatively narrow relative to the width of the distal articulation. In Ornithomimidae [36,128] and Caenagnathidae, the shaft of metatarsal III is broad between metatarsals II and IV, approaching the width of the distal articulation at its widest point, and expanded medially and laterally out over II and IV to broadly overly them.

8. **Metatarsal III, proximal end of shaft in dorsal (extensor) view: continuous with rest of shaft (0), or asymmetrical, medially skewed dorsal expansion of the shaft of metatarsal III (1).**

   NEW CHARACTER. Compared to other theropods, where the metatarsal shaft is unexpanded or symmetrically expanded, in tyrannosaurids [34,39] the shaft flares more medially towards MT II, and flares only weakly towards MT IV. A proximodistally-running tangent line would intersect more proximally with the medial curve, and more distally with the lateral curve. In contrast, the shaft is symmetrically flared in Ornithomimidae [36,132] and Caenagnathidae, and in Troodontidae [42] the expansion is laterally skewed. Not applicable to alvarezsaurids, where the proximal end of metatarsal III is absent.

9. **Metatarsal III, ventral surface bearing large, pillar-like tubercle on ventral surface of metatarsal III absent (0) or present (1).**

Yun [39].

The ventral surface of the metatarsus in *Tyrannosaurus* has a distinct pillar-like ridge or tubercle, which lies distal to the contact between metatarsals II and IV, and proximal to the distal articular surface (FMNH PR 2081; see also Figure S2). A small bump or continuous ridge is present in the same position in Albertosaurinae (Figure S2). In *Daspletosaurus* there is a well-developed tubercle but it does not form the distinctive pillar-like structure seen in *T. rex* [39]. This feature is also shared with *Tarbosaurus baatar* [39] among Tyrannosauridae.

10. **Dentary, interdental plates: present (0), or absent (1).**

Currie [135]; Makovicky and Sues [136]: Character 29, Brusatte et al. (2015): Character 88. Dentary, inter/paradental plates: present (0), or absent (1). There has historically been some debate as to whether such plates are absent or fused in taxa where distinct triangular or rectangular structures bordering the alveoli medially are absent. As shown by Leblanc et al. (2017), 'interdental plates' are not distinct bony plates, but represent alveolar bone deposits, which can take on different morphologies. Here we do not distinguish between plates 'fused' or 'absent', but simply observe whether defined triangular or rectangular structures are present along the dentary or not. Large interdental plates are present in Tyrannosauridae [32,34]. Interdental plates are fused to the dentary or absent in Dromaeosauridae and Troodontidae.

11. **Interdental plates: well developed in the back of the jaw (0), or highly reduced posteriorly (1).** Interdental plates are well-developed in the posterior part of the jaw, above the posterior end of the Meckelian groove, in Nanotyrannidae [46]. They are reduced behind the Meckelian groove in Albertosaurinae [61]. The interdental plates are highly reduced above the posterior end of the Meckelian groove in *Tyrannosaurus* [46] and *Tarbosaurus* [137].

12. **Dentition, teeth present (0), absent (1).**

Makovicky and Sues [136]: Characters 1, 2.

Dentition is present in Tyrannosauridae, Nanotyrannidae, Dromaeosauridae, Troodontidae, and most likely Alvarezsauridae among North American theropods. Teeth are absent from Ornithomimidae and Caenagnathidae.

13. **Premaxillary teeth, lack lingual ridge (0) or prominent lingual ridge running apico-basally, and bordered on either side by broad sulci between the ridge and the mesial and distal carinae (1), or lingual ridge expanded, and with narrow gutters separating the lingual ridge and carinae (2).**

Modified from Brusatte et al. [127]: Character 648.

In Nanotyrannidae and Tyrannosauridae, a large apico-basal ridge extends from the base of the crown to the apex, flanked on either side by a broad gutter (Nanotyrannidae, Albertosaurinae) or a narrow groove (*Tyrannosaurus*). This ridge is absent in Saurornitholestinae, where the premaxillary teeth are medially flat [138]. In Dromaeosaurinae, there is a low, poorly defined lingual ridge on the lingual surface of the

premaxillary teeth [139]. In Troodontidae the lingual surface of the premaxillary teeth is broadly convex [135].

14. **Premaxillary teeth, serrated (0) serrated distal but not mesial carinae (1), or mesial and distal carinae unserrated (2).** Both carinae are serrated in Tyrannosauridae, Eudromaeosauria, and Troodontidae. In Nanotyrannidae, mesial carinae are unserrated, only a few faint serrations are present on the distal carina and so it is coded as State 2.

15. **Premaxillary teeth, slight twist of carinae lingually and teeth mesiodistally broad, bladelike, or weakly D-shaped (0) or strongly D-shaped morphology with a strong twist of mesial carina onto posteromedial surface of tooth, and tooth crown mesiodistally compressed (1).**

    Modified from Brusatte, Lloyd, Wang and Norell [127]: Character 89.

    In most theropods, such as dromaeosaurids [138,139] and troodontids (Currie, 1987) the mesial carina is slightly shifted towards the posterior surface of the tooth. In tyrannosaurids [13,54,140], it is strongly twisted onto the posteromedial surface, typically creating a “D-shaped” tooth with both carinae lying posteriorly, and the crown is mesiodistally compressed, and longer labiolingually than mesiodistally. Although some theropods such as *Allosaurus* approach a D-shaped morphology with a posteriorly shifted mesial carina [141] they lack the strong mesiodistal compression seen in the teeth of Tyrannosauridae.

16. **Lateral teeth, bases subrectangular in section, pinched or teardrop-shaped (0) or rectangular-ovate (1).** In Nanotyrannidae and Albertosaurinae, the tooth bases are relatively narrow and rectangular. In *Tyrannosaurus*, the tooth bases have a more ovate shape associated with the overall swelling of the crown, particularly on the labial edge.

17. **Lateral teeth, serrated mesial and distal carina (0) unserrated mesial carina (1), unserrated mesial and distal carinae (2).**
    Modified from Brusatte, Lloyd, Wang and Norell [127]: Character 81.

18. **Lateral teeth, carinae aligned anteroposteriorly or slightly twisted, (0) or carinae strongly twisted, mesial carina shifted lingually, and distal carina shifted laterally (1).**

    NEW CHARACTER.

    In the lateral teeth of most theropods, including Dromaeosauridae [45,142] and Troodontidae [135], the mesial and distal carinae lie roughly anterior and posterior, along the long axis of the tooth cross section. In Tyrannosauridae, the carinae are twisted. The mesial carinae start on the anterior edge of the tooth, and twist onto the anteromedial surface of the tooth towards the base of the crown [54]. The distal carina is shifted such that it ends on the posterolateral surface of the crown [54].

19. **lateral teeth, carinae: serrations contiguous over apex of tooth, where they meet distal carina (0), or serrations terminate below apex (1).** NEW CHARACTER.

20. **Mesial carinae: denticles large (0) small relative to distal carina denticles (1).**

Currie, Rigby and Sloan [142].

Denticles are similar in size on the anterior and posterior carinae primitively in theropods, a condition retained in Tyrannosauridae. Denticles are reduced on the anterior carinae in many dromaeosaurs, or enlarged in some Troodontidae.

MATRIX

```
MATRIX
Allosauroidea      00000000000000000000
Ornithomimidae     100000100--1--------
Caenagnathidae     110010100--1--------
Alvarezsauridae    11?0100001-0020020-2
Troodontidae       1201?10001-0000-0010
Eudromaeosauridae  0200?10000-000000010
Microraptorinae    1?00?10?00-00-0--010
Nanotyrannidae     10111000000012100100
Albertosaurinae    11111001000010100100
Tyrannosaurus_rex  1121100110102-110100
RSKM_P2416.82      1121100?1???????????
TMP1981.16.475_    1111?00?0???????????
YPM_55587          ???????????0121?????
YPM_55604          ???????????0211?????
YPM_55564          ???????????0???111?1
YPM_54474          ???????????0???111?1
YPM_55551          ???????????0??-111?1
FMNH_PR_4290       ?????????010????0100
```

Analysis. Analyses were run as a branch-and-bound search on PAUP 4.0 b10. The following backbone constraint tree was used:

constraints Theropoda (backbone)= (((Ornithomimidae,(Alvarezsauridae,(Caenagnathidae,(Troodontidae,(Microraptorinae,Eudromaeosauridae))))),(Nanotyrannidae,(Albertosaurinae,Tyrannosaurus_rex))),Allosauroidea);

hsearch constraints=Theropoda enforce=yes

```
/-------------------------------------------------------------------------- Allosauroidea
|
|                                   /---------------------------------- Nanotyrannidae
|                                   |
|         /-------------------------+         /---------------------- Albertosaurinae
|         |                         |         |
|         |                         |         +---------------------- TMP1981.16.475
|         |                         \---------+
|         |                                   |      /------------ Tyrannosaurus rex
\---------+                                   \------+
          |                                          \------------ RSKM P2416.82
          |
          |        /--------------------------------------------------------- Ornithomimidae
          |        |
          \--------+         /--------------------------------------------- Alvarezsauridae
                   |         |
                   \---------+         /---------------------------------- Caenagnathidae
                             |         |
                             \---------+         /---------------------- Troodontidae
                                       |         |
                                       \---------+      /------------ Eudromaeosauridae
                                                 \------+
                                                        \------------ Microraptorinae
```

Figure S4. Constrained phylogenetic analysis of metatarsals. TMP 1981.16.475 emerges unresolved within Tyrannosauridae; RSKM P2416.82 clusters with *T. rex*. Tree length = 32 Consistency index (CI) = 0.7500, Retention index (RI) = 0.7500

```
/-------------------------------------------------------------------------- Allosauroidea
|
|                  /---------------------------------------------------------- Ornithomimidae
|                  |
|         /--------+         /--------------------------------------------- Alvarezsauridae
|         |        |         |
|         |        \---------+         /---------------------------------- Caenagnathidae
|         |                  |         |
|         |                  \---------+         /---------------------- Troodontidae
|         |                            |         |
|         |                            \---------+      /------------ Eudromaeosauridae
\---------+                                      \------+
          |                                             \------------ Microraptorinae
          |
          |                                             /------------ Nanotyrannidae
          |                            /----------------+
          |                            |                \------------ YPM 55587
          |                            |
          \----------------------------+         /---------------------- Albertosaurinae
                                       |         |
                                       |         |      /------------ Tyrannosaurus rex
                                       |         |      |
                                       \---------+      +------------ YPM 55604
                                                 |      |
                                                 |      +------------ YPM 55564
                                                 \------+
                                                        +------------ YPM 54474
                                                        |
                                                        +------------ YPM 55551
                                                        |
```

\----------- FMNH PR 4290

Figure S5. Constrained phylogenetic analysis of teeth and jaw. Strict consensus of 51 trees. Tree length = 34; Consistency index (CI) = 0.7353, Retention index (RI) = 0.7805

Other material attributed to juvenile or embryonic Tyrannosauridae. Funston et al. [13] report an ungual (UALVP 59599) as pertaining to a ~1 meter long embryonic tyrannosaurid. The ungual does not resemble unguals of tyrannosaurids such as *T. rex* [34] or *Albertosaurus sarcophagus* [44] or show any derived features unique to Tyrannosauridae, or a combination of primitive and derived features that would support referral to Tyrannosauridae.

Tyrannosaurid unguals tend to be relatively elongate, with strongly curved dorsal margins, and with a strongly tapered shape in dorsal or ventral view. Instead, UALVP 59599 more closely resembles that of Ornithomimosauria [128,143]. Features shared with Ornithomimosauria include a relatively straight claw, and the claw resembles *Struthiomimus altus* in being very short, broad and hooflike in dorsal or ventral view; specifically its shape closely resembles that of *S. altus* II-3 in dorsal view. Furthermore, the claw resembles *S. altus* in having a broadly rounded dorsal margin proximally, which narrows to a sharp dorsal ridge distally [143]. Accordingly we interpret this fossil as representing a juvenile or embryonic ornithomimid, related to or referable to *Struthiomimus altus*.

MOR 268, a dentary referred to *Daspletosaurus horneri* does conform to the morphology of Tyrannosauridae. However, the low angle formed by the symphysis and pointed tip of the jaw, as well as the weak upturn of the tip of the dentary better match those of *Gorgosaurus libratus*, e.g. TMP 91.36.500 and TMP 94.143.1 (see [61]) than *Daspletosaurus horneri* (MOR 590, see [144]) where the anteroventral margin of the symphysis rises up steeply and the anterior end of the dentary is strongly upturned. Accordingly we tentatively assign this specimen to Albertosaurinae.

2. Size Estimation

Mass estimation was conducted by measuring the length and width of the distal end of metatarsal III in extant birds to create a regression between the dimensions of the articular surface and body mass.

86 skeletons of 78 species were measured from the Yale Peabody Museum ornithology collections. To improve the accuracy of the regression, which is highly sensitive to outliers, birds spanning a wide range of body sizes were sampled, from 33 g to 111,000 g, targeting birds from the upper and lower end of these ranges. Volant and flightless birds were used, but no clear difference between them was visible when the data were plotted, validating this approach for flightless dinosaurs.

In some cases, associated mass data were available from the specimen itself. In other cases, data on mass was taken from the Handbook of Avian Body Masses [145]. When sex was known for the specimen and sex-specific mass averages were available, sex-specific mass was used. Otherwise, the species average for males and females was used. Individual skeletons may represent individuals either above or below the species average, but such differences should cancel out across the large number of individuals sampled. In some cases, average species masses were not available, only ranges. In these cases, the median mass was used.

Dimensions of the distal articular surface are:

8 mm wide x 7.5 mm tall for *T. rex* (RSKM P2416.82);
7.5 mm wide x 7.8 mm tall for *Gorgosaurus libratus* (TMP1981.16.475).

Data were logarithmically transformed (Log10) prior to regression analysis.

Three correlations between mass and a MT III measurement were calculated using both Ordinary Least Squares (OLS) and Phylogenetic Generalized Least Squares (PGLS) linear regression models, using a densely sampled avian phylogeny from Jetz et al [24]. Log likelihoods were calculated for each regression and combination of independent and dependent variables to determine which of the six regression models best fit the data, and this was determined to be OLS regression of mass on the distal articular area of MT III. PGLS on the distal width of MT III fit nearly as well when assessed by Log-Likelihood score. We therefore selected those two models to determine a best fit estimate for the mass of the two tyrannosaurid metatarsals as well as their associated 95% prediction interval. These mass estimates as well as slopes, intercepts, and log likelihoods are summarized in Table S2.

Best fit mass estimates range from 2459 g to 3425 g with a cumulative 95% prediction interval ranging from 1255 g to 6310 g. These values bracket the 5 kg value assumed by Erickson et al. [7] for a hatchling *T. rex* to constrain their growth study. For calculation of clutch size, we chose the OLS model, as it had the highest Log-Likelihood score.

Table S2. Slopes, intercepts and standard errors for OLS and PGLS regressions of avian body mass on three measures of MT III condyle in birds. Best fitting models (OLS for MT distal articulation area and PGLS for MT width) are marked in boldface and used to estimate body mass and 95% prediction interval for both the RSKM 2416.82 and TMP1981.16.475 metatarsals. Mass estimate ranges reported in the text represent the range between the two best fitting means. The cumulative 95% prediction interval reported in the text extends from the lower bound of the 95% prediction interval for the lower mass estimate (OLS of MT area) to the upper bound of the 95% prediction interval of the higher estimate (PGLS of MT width), thus capturing the extremes plus the overlap of both estimates.

Inferred body mass for hatchling *T. rex* (RSKM P2416.82) as a function of distal MT III measurements

**Measurement**	**Blomberg's K**	**OLS/ Pgls**	**Slope**	**Std. error**	**Intercept**	**Std. error**	**LogLikelihood**	**RSKM P2416.82 mass**	**lwr 95% prediction limit**	**upper 95% prediction limit**
MTIII width	0.9513549	OLS	2.254459	0.06509003	1.516075	0.04516119	28.03865	3565 g		
		PGLS	2.269777	0.08074762	1.484863	0.09887641	35.42229	3425 g	1585 g	6310 g
MTIII height	0.8648262	OLS	2.21272	0.0692771	1.281912	0.05562003	22.9834	1653 g		
		PGLS	2.056834	0.1094003	1.411233	0.1459545	11.79856	1626 g		
MTIII area	0.9235752	**OLS**	**1.1340954**	**0.02899469**	**1.374149**	**0.04332137**	**35.70045**	**2459 g**	**1264 g**	**4783 g**
		PGLS	1.116321	0.04266597	1.389471	0.10797555	31.06817	2369		

Inferred body mass for hatchling G. *libratus* (TMP 1981.16.475) as a function of distal MT III measurements

**Measurement**	**Blomberg's K**	**OLS/ Pgls**	**Slope**	**Std. error**	**Intercept**	**Std. error**	**LogLikelihood**	**TMP 1981.16.475 mass**	**lwr 95% prediction limit**	**upper 95% prediction limit**
MTIII width		PGLS	**2.269777**	**0.08074762**	**1.484863**	**0.09887641**	**35.42229**	2958 g		
MTIII area		OLS	**1.1340954**	**0.02899469**	**1.374149**	**0.04332137**	**35.70045**	2389 g		

Table S3. Regressions for determining body size from metatarsal dimensions.

Mass (M) in grams versus articular surface width (W) in millimeters:

$M = 2.2612W + 1.5053$. ($R^2 = .9675$)

Mass (M) in grams versus articular surface height (H) in millimeters:

$M = 2.3039H + 1.2201$. ($R^2 = .9586$)

Mass (M) in grams versus articular surface height (W*H) in millimeters:

$M = 1.1529 (W*H) + 1.3467$ ($R^2 = .973$)

In addition, comparisons of the isolated metatarsal III with other Tyrannosaurus (LACM 7244-23744) were used to estimate the length of the complete metatarsal III (Figure S6).

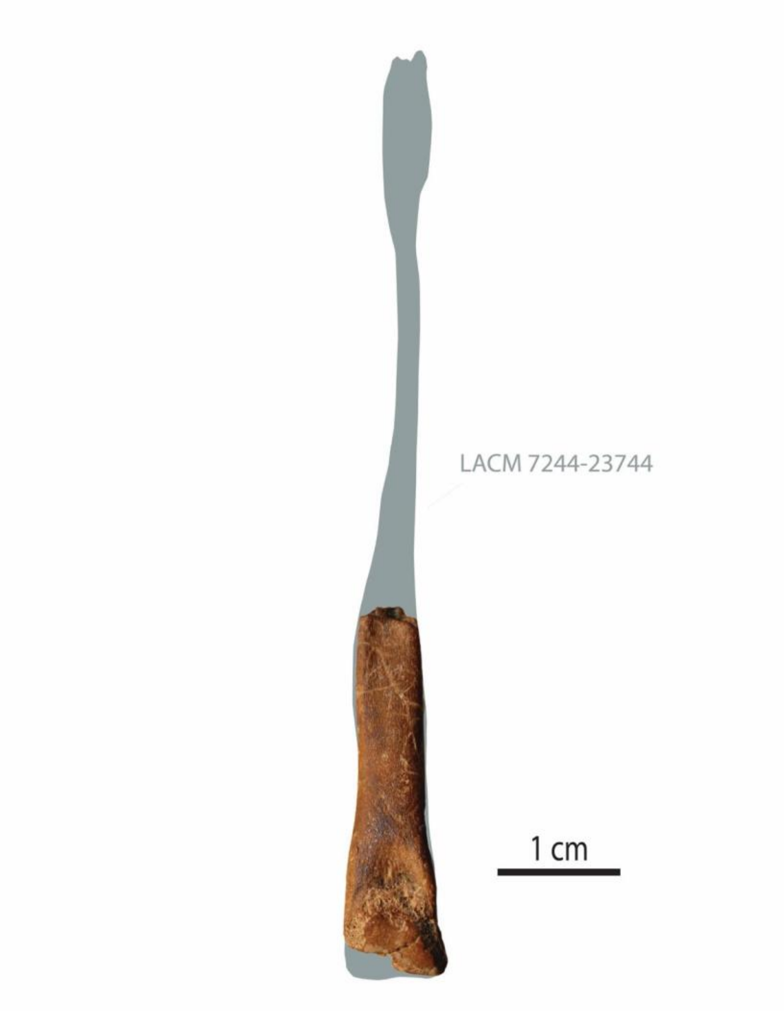

Figure S6. *Tyrannosaurus rex* hatchling metatarsal, RSKM P2416.82. Missing portions reconstructed after LACM 7244-23744, suggesting a length of ~ 75 mm.

Juvenile *Tyrannosaurus* are only known from relatively large and fragmentary individuals (LACM 7244-23744) and so a skeletal reconstruction of a juvenile *Tarbosaurus* was used as a stand-in, with the skeletal reconstruction scaled to fit the metatarsal [146]. This suggests a length for RSKM P2416.82 of approximately 70 cm (Figure 2).

3. Growth Curves

For tyrannosaurs, age values were determined by taking the total number of lines of arrested growth (LAGs) from the literature, as described in Table S4, and adding 0.5 to obtain an estimate of age in years (i.e., accounting for the fact that some amount of growth can occur after the last deposited LAG).

Mass values were determined from the literature with some corrections, as described in S2.

Growth curves were calculated in R.

Best-fitting logistic curves were first calculated for the data (either excluding or including debated *Nanotyrannus* specimens in the *T. rex* data), while including the hatchling datapoints described in this study for *T. rex* and *Gorgosaurus*. For taxa with hatchling mass data (i.e., *T. rex* and *Gorgosaurus*), the best-fitting curves were then vertically transformed along the y-axis such that hatchling mass at age = 0 was equal to the fossil evidence (i.e, by subtracting the estimated mass at age = 0 from the original fitted regression by the observed empirical mass estimate derived from the smallest hatchling fossils, and then subtracting the y-values for mass across the entire regression by this correction value to yield a more realistic growth curve at very young ages). The corrections subtracted from the original regressions to yield juvenile growth curves consistent with the empirical hatchling mass estimates from fossils were as follows: 22.69467 kg for *T. rex* and *Nanotyrannus* combined, 52.57274 kg for *T. rex* alone, and 63.44671 kg for *Gorgosaurus*.

For extant taxa, growth curves were analysed using the reported parameters from previous studies rather than through independent regression analysis of the raw data. Male and female *Rhea* from Navarro et al. 2005 were modelled with Gompertz curves, converting g to kg and days to years. Male and female *Struthio* from Ramos et al. [108] were modelled with logistic curves with the lowest AIC scores, converting days to years. Male and female *Alligator* from Wilkinson & Rhodes 1997 were modelled using von Bertalanffy curves, using Farlow et al. [110] to convert length to body mass. The reported relationship between total length in mm and body mass in kg is $\log_{10}(M) = 3.47 * \log_{10}(L) – 10.03$ in Farlow et al. [110]. Dhofari cattle from Bahashwan et al. 2015 were modelled using the von Bertalanffy curve with the highest R-squared value, converting months to years. Male and female Asian elephant from Mumby et al. [112] were modelled using von Bertalanffy curves (wild and captive data combined). Growth curves are shown in Figure 18 and Figure S7, and summary statistics in Table S5.

The updated weight parameters (rounded) for Mumby et al. (2015) are as follows:

female, captive only	K = 0.081, t0 = -0.994
female, wild & captive	K = 0.085, t0 = -0.916
female, longitudinal	K =0.091, t0 = -0.713
male, captive	K = 0.058, t0 = -1.609
male, wild & captive	K = 0.058, t0 = -1.661
male, longitudinal	K = 0.071, t0 = -0.821

Table S4. Tyrannosaur data with sources and corrections. DME = developmental mass estimation. Specimens used to establish adult mass for DME are in bold. Hatchling and juvenile specimens described in this study are in lighter colours.

Taxon	Specimen #	Mass (kg)	Log_{10}(Body mass	LAGs	Age (year	Relevant sources	Notes
Tyrannosaurus rex	AMNH 30564	2340.28049	3.369267912	15	15.5	Erickson et al. (2004); Horner & Padian (2004); Lee & Werning (2008);	New DME mass based on new adult mass estimate revised from Campione et al. (2014) with FMNH PR2081 (SUE) as the base femur
Tyrannosaurus rex	LACM 23845	2401.412178	3.380466709	14	14.5	Erickson et al. (2004); Horner & Padian (2004); Lee & Werning (2008);	New DME mass based on new adult mass estimate revised from Campione et al. (2014) with FMNH PR2081 (SUE) as the base femur
Tyrannosaurus rex	ICM 2001.90.1	3965.586021	3.598307375	16	16.5	Erickson et al. (2004); Horner & Padian (2004); Lee & Werning (2008);	New DME mass based on new adult mass estimate revised from Campione et al. (2014) with FMNH PR2081 (SUE) as the base femur
Tyrannosaurus rex	MOR 1125	3351.611241	3.525253638	18	18.5	Erickson et al. (2004); Horner & Padian (2004); Lee & Werning (2008);	New DME mass based on new adult mass estimate revised from Campione et al. (2014) with FMNH PR2081 (SUE) as the base femur
Tyrannosaurus rex	RTMP 81.6.1	4292.507656	3.632711078	18	18.5	Erickson et al. (2004); Horner & Padian (2004); Lee & Werning (2008);	New DME mass based on new adult mass estimate revised from Campione et al. (2014) with FMNH PR2081 (SUE) as the base femur
Tyrannosaurus rex	RTMP 81.12.1	6697.906683	3.825939093	22	22.5	Erickson et al. (2004); Horner & Padian (2004); Lee & Werning (2008);	New DME mass based on new adult mass estimate revised from Campione et al. (2014) with FMNH PR2081 (SUE) as the base femur
Tyrannosaurus rex	**FMNH PR2081**	**7377**	**3.867879783**	**28**	**28.5**	**Campione et al. (2014) for mass; Erickson et al. (2004) &**	
Tyrannosaurus rex	RSM P2691.1 17393	75	1.875061263	3	3.5	This study	See this study for details
Tyrannosaurus rex	RSM P2416.82	2.5	0.397940009	0	0.5	This study	See this study for details
Nanotyrannus lancensis?	LACM 28471	39.86849216	1.600629811	2	2.5	Erickson et al. (2004); Horner & Padian (2004); Lee & Werning (2008); Saitta et al. (2020)	The DME value (with FMNH PR2081 [SUE] as the base femur) for LACM 28471 is compromised due to the absence of a complete femur; the one measured in the paper is an Alberstosaurus femur added to the cast in the UK by RCI to make it 'complete'. The age estimate should be from Cullen et al (2020); Woodward et al. (2020) overcounted a multi-LAG.
Nanotyrannus lancensis?	BMRP 2002.4.1	954	2.979548375	13	13.5	Hutchinson et al. (2011); Woodward et al. (2020); Cullen et al. (2020)	The Hutchinson et al. (2011) value for BMRP 2002.4.1 derived from the 3D model (954 kg) is probably reliable.
Gorgosaurus libratus	FMNH PR 2211	205.7285068	2.313294474	5	5.5	Erickson et al. (2016)	New DME mass based on new adult mass estimate revised from Campione et al. (2014) with RTMP 94.12.602 as the base femur
Gorgosaurus libratus	RTMP 86.144.1	370.959276	2.569326235	7	7.5	Erickson et al. (2016)	New DME mass based on new adult mass estimate revised from Campione et al. (2014) with RTMP 94.12.602 as the base femur
Gorgosaurus libratus	RTMP 73.30.1	1210.072398	3.082811355	14	14.5	Erickson et al. (2016)	New DME mass based on new adult mass estimate revised from Campione et al. (2014) with RTMP 94.12.602 as the base femur
Gorgosaurus libratus	RTMP 99.33.1	983.2850679	2.992679444	14	14.5	Erickson et al. (2016)	New DME mass based on new adult mass estimate revised from Campione et al. (2014) with RTMP 94.12.602 as the base femur
Gorgosaurus libratus	**RTMP 94.12.602**	**1790**	**3.252853031**	**18**	**18.5**	**Bredely et al. (2015); Erick**	**Mass recalculated using Campione et al. (2014) equation 7 applied to femoral circumference = 330 mm from Bredely et al. (2015); old mass estimate from Erickson et al. (2016) = 1105 kg**
Gorgosaurus libratus	TMP1981.16.475	2.4	0.380211242	0	0.5	This study	See this study for details
Albertosaurus sarcophagus	RTMP 2002.45.46	63.46961471	1.802565862	2	2.5	Erickson et al. (2016)	New DME mass based on new adult mass estimate revised from Campione et al. (2014) with RTMP 81.10.1 as the base femur
Albertosaurus sarcophagus	RTMP 86.64.01	961.5078809	2.982952848	15	15.5	Erickson et al. (2016)	New DME mass based on new adult mass estimate revised from Campione et al. (2014) with RTMP 81.10.1 as the base femur
Albertosaurus sarcophagus	USNM 12814/ AMNH 5428	1278.225044	3.106607322	18	18.5	Erickson et al. (2016)	New DME mass based on new adult mass estimate revised from Campione et al. (2014) with RTMP 81.10.1 as the base femur
Albertosaurus sarcophagus	AMNH 5432	1617.654991	3.208885902	22	22.5	Erickson et al. (2016)	New DME mass based on new adult mass estimate revised from Campione et al. (2014) with RTMP 81.10.1 as the base femur
Albertosaurus sarcophagu	**RTMP 81.10.1**	**1441**	**3.158663981**	**24**	**24.5**	**Bredely et al. (2015); Eric**	**Mass recalculated using Campione et al. (2014) equation 7 applied to femoral circumference of 305 mm from Bredely et al. (2015); old mass estimate from Erickson et al. (2016) was 1105 kg**

Table S5. Summary statistics for growth models of Tyrannosauridae and extant amniotes, ranked by age at maximum growth rate from youngest to oldest (i.e., protractedness of growth curve). Adult size is an average deriving from the estimated asymptote of the growth curve. Maximum growth rate (i.e., highest derivative value) and the age at maximum growth rate (i.e., inflection point) are likewise derived from the fitted growth curves. *Adult size for *Gorgosaurus*, *T. rex* + *Nanotyrannus*, and *T. rex* alone are the asymptote values prior to the hatchling size correction; given the very small sizes of hatchling relative to adults, these corrections (of ~20–60 kg) have minimal impact on the estimate for average adult asymptotic size and the non-corrected curve asymptotes are expected to be better estimates for average adult mass than the corrected curve asymptotes. Sex: M = male, F = female, B = both, U = unknown. **Age of 0 yr derives from the model used by Mumby et al. (2015), in which slope continuously decreases with age.

**Taxa**	**Sex**	**Adult size (kg)**	**Maximum growth rate (kg/yr)**	**Age at maximum growth rate (yr)**
Elephant	M	3407	179.4573	0**
Elephant	F	2548	200.3568	0**
Rhea	M	28.7063	41.81754	0.375
Rhea	F	22.5074	36.62418	0.375
Cattle	B	322	149.408	0.425
Ostrich	M	103.7	170.3173	0.505
Ostrich	F	101.7	176.3078	0.51
Alligator	F	83.3365	3.332683	12.42
Albertosaurus	U	1569.9217	146.2382	14.2649
Alligator	M	244.274	7.331796	17.01
Gorgosaurus	U	3121.6738*	174.1114	17.2007
T. rex + *Nanotyrannus*	U	7731.094*	620.4203	17.829
T. rex	U	7944.1463*	549.5363	17.9421

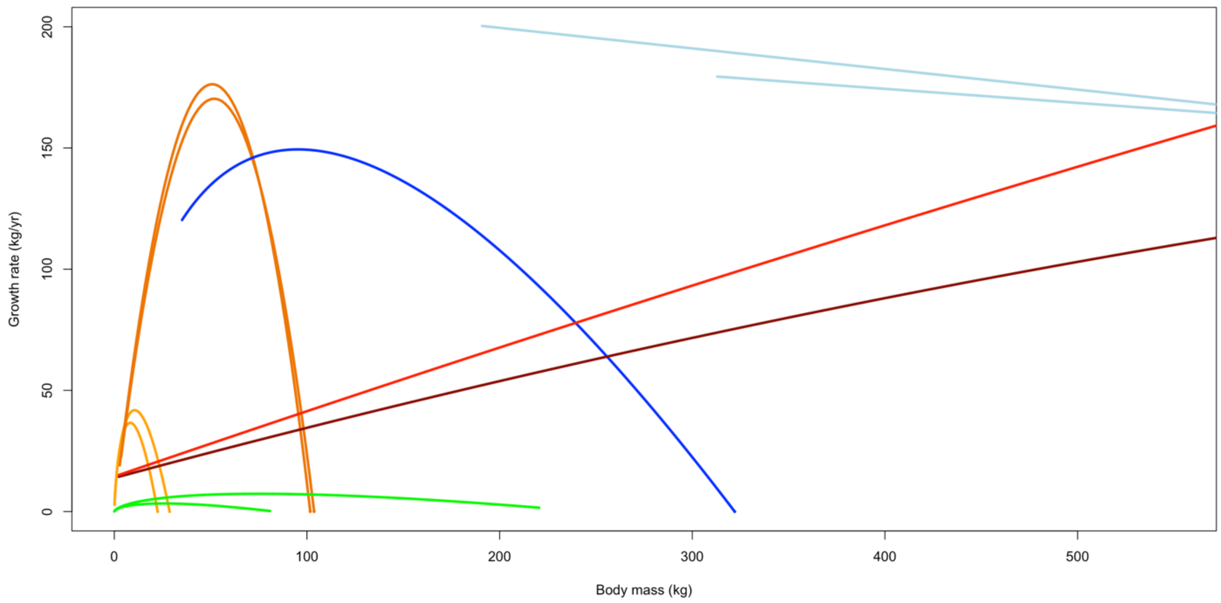

Figure S7. Growth rates (kg/yr) versus body mass for juvenile *T. rex* and *G. libratus*; a mammal (*Bos indicus*), ratites (*Rhea americana*, *Struthio camelus*) and a crocodilian (*Alligator mississippiensis*). As young juveniles, tyrannosaurids grew faster than equivalently sized crocodilians, but much slower than equivalently sized hatchling ratites or new-born mammals (note that mammal mass at birth is much greater than archosaur mass at hatching). (Specimens whose taxonomic status remains contested have been excluded in these plots). Data Sources: Data for *Rhea* from Navarro et al. (2005); for *Struthio* from Ramos et al. (2013); for *Alligator* from Wilkinson and Rhodes (1997) using Farlow et al. (2005) to convert length to mass; for cattle from Bahashwan et al. (2015); for Asian elephant from Mumby et al. (2015).

4. Estimation of Reproductive Parameters

Egg mass. Egg masses were estimated using estimated body masses of *T. rex* and *G. libratus* (Table S6) and then estimating the egg mass using a crocodilian model (in which the hatchling is 65% of egg mass) [50] and an avian model (in which the hatchling is 67.2% egg mass) [51].

We then compared estimates for *T. rex* neonate / egg masses against predictions of egg size for birds and crocodilians as phylogenetically appropriate models bracketing the possible range of masses in non-avian dinosaurs. We used a series of analyses, both phylogenetic and non-phylogenetic, and a range of datasets, including birds, crocodilians, and Diapsida (birds, crocodilians, squamates, turtles, and non-avian Dinosauria).

Table S6. Estimated masses for juveniles and eggs of *Tyrannosaurus rex* and *Gorgosaurus libratus* based on two different mass estimates (OLS and PGLS regressions) and both avian and crocodilian egg models.

	Mass	Egg mass (avian model hatchling = .672 egg mass)	Egg mass (crocodilian model, hatchling = .65 egg mass)
T. rex (OLS)	2459 g	3659 g	3783 g
95% prediction interval	(1264 g - 4783 g)	(1881g -7118 g)	(1945-7358 g)
T. rex (PGLS)	3425 g	5097 g	5269 g
95% prediction interval	(1585 g – 6310 g)	(2359 g – 9390 g)	(2438 g – 9708 g)
T. rex (OLS)<br>(at hatching)	1668 g	2482 g	2566 g
95% prediction interval	852-3217	(1267 g – 4787 g)	(1310 g – 4949 g)
G. libratus (OLS)	2389 g	3675 g	3675 g
G. libratus (PGLS)	2959 g	4403 g	4552 g
G. libratus (OLS)<br>(at hatching)	1325 g	1972 g	2039 g

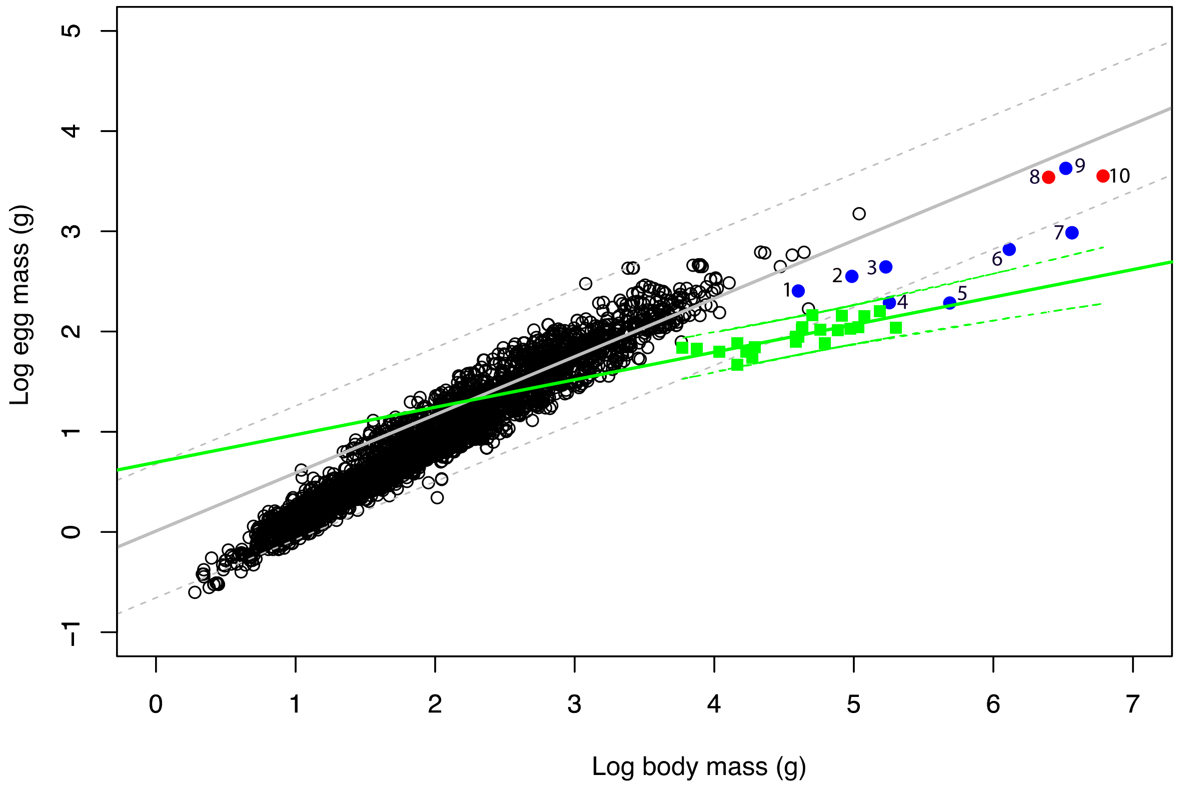

Figure S8. Plots of egg size as a function of body mass in birds (black circles), crocodilians (green) with OLS regressions and 95% prediction intervals. Red dots = *Gorgosaurus libratus* and *Tyrannosaurus rex*; blue = other non-avian dinosaurs. Non-avian dinosaurs are identified by numerals as follows: 1, *Troodon*, 2 *Deinonychus*, 3, *Citipati*, 4, *Protoceratops* 5, *Massospondylus*, 6, *Mussaurus*, 7 *Maiasaura*, 8, *Gorgosaurus*, 9 *Hypacrosaurus*, 10 *Tyrannosaurus*. 9-10 based on results of this study, other data provided in SI Data2.

To predict egg size in a gravid *T. rex*, we used Ordinary Least Squares and Phylogenetic Generalized Linear Model linear correlations of log-transformed egg mass against log transformed body mass for both clades (Figure S8), with body- and egg mass data taken from a recent data set for amniotes [20]. For the avian PGLS regression, we used the Jetz et al. [24] phylogeny, because of its species-rich sampling. Specifically, we sampled a single tree with the Hackett et al. [25] ordinal- level phylogeny as a backbone from the 1000 trees in the dataset available on Dryad to expedite processing time. For extant crocodilians, we used the phylogeny of Colston et al. [26] for PGLS analysis, as it has the most complete taxon sampling. Branch lengths were left unmodified in the source trees.

The phylogenetic signal in body mass and egg mass data was assessed using the K metric (Blomberg et al., 2003). We evaluated model fit to the data using log likelihood scores with a ∂Log likelihood ≥ 2 taken as significant and ≥ 10 as highly significant following common practice.

We used the better fitting model (either OLS or PGLS) for each clade to estimate the 95% prediction interval for the *T. rex* egg mass and clutch mass estimates. As R does not have a standard command to generate prediction intervals for GLS models, we modified a script to allow their calculations and used it for all comparisons. All analyses were performed in R version 4.1.2 (2021-11-01) the R Studio interface, and the code and data are provided in the appendices. Results are summarised in Table S6.

Combining the list of bird taxa with known body- and egg mass with the Jetz et al [24] phylogeny yielded a dataset of 3701 taxa with egg and clutch masses, a third of extant avian diversity. This large dataset exhibits a strong phylogenetic signal (Blomberg's K = 2.072; p = 0.01) and a large difference in log likelihoods between the OLS and PGLS correlations (∂logLikelihood = 132.093). Comparison of the trendlines shows that the OLS line has a steeper slope but lower intercept than that for PGLS. Whereas the OLS trendline shows a better fit to data points close to the origin, the PGLS trendline exhibits a better fit through the cluster of datapoints for the largest birds, which are ratites.

Extrapolations for egg size in *T. rex* are based on the estimated body mass of MOR 1125, identified as a gravid female due to presence of medullary bone [22]. Using a modified version of the formula for body size estimation in bipeds [23], Persons et al. [21] derive a body mass estimate of 6100 kg for this individual.

Based on that body mass estimate, our best fit OLS model for avian data (Figure S8) arrives at a corresponding estimate of 66.31 kg for egg mass, with a 95% prediction interval ranging from 54.8 kg to 80.3 kg. The corresponding PGLS estimate yields a much lower value of 8.8 kg, though with a 95% prediction interval of 2.1 kg to 40.9 kg. The OLS derived values for the bird data exceed the maximum known size for a bird egg, seen in the elephant bird, *Aepyornis maximus*. Eggshell thickness scales positively with egg mass, and is thought to set an upper limit to egg size as constrained by the ability of the hatchling to break out of the shell [147,148], and the OLS extrapolations might be biologically infeasible. By contrast, the best fit PGLS value is comparable to the size of an *Aepyornis* egg. Assuming a similar eggshell thickness to *Aepyornis*, it is impossible for eggs of this size to have been able to withstand being directly brooded by a *T. rex* adult weighing multiple tons [147,148]. Therefore, *T. rex* likely buried its eggs as in living reptiles, possibly to extended incubation periods as in other non-avian dinosaurs (Erickson et al., 2017).

Fits for the OLS (Figure S8) and PGLS models to the crocodilian data are not significantly different from each other, although the slopes and intercepts differ, and phylogenetic signal in the data is weak (K = 0.32). Based on crocodilian data, estimates for average egg mass in a 6100 kg *T. rex* are 382 g (95% prediction interval ranges from 189 to 692 grams) for the better fitting OLS model and 284 g for PGLS, and thus about an order of magnitude smaller than the estimated average egg mass for the RSKM P2416.82 neonate.

Finally, an Ordinary Least Squares regression was done first for all diapsids, including extant birds, non-avian dinosaurs, squamates, and chelonians (Figure S9). This regression shows a very high R2 despite the wide range of taxa included, and despite having a very different developmental strategy, large placentals fall along this same regression line when mammals are included in the dataset).

Clutch size shows a poorer correlation with size (Figure 17) than clutch mass; accordingly we attempted to infer tyrannosaur clutch size from clutch mass and egg size (Table 3).

Table S7. Summary statistics for correlations between log body mass and log egg mass, and log body mass and log clutch mass in birds and crocodilians using both Ordinary Least Squares (OLS) and Phylogenetic Generalized Least Squares (PGLS) regressions. Statistics include Blomberg's K (a measure of phylogenetic signal) slopes, intercepts and their associated standard errors, and the log likelihood for each regression. The log likelihoods are used to determine which of the two regression models yields a better fit for each comparison. Predicted egg and clutch masses are calculated for a 6100 kg *T. rex* for each regression model and 95% prediction intervals are provided for the better fitting model, and for both models for avian egg mass.

Log transformed egg mass as a function of log transformed body mass

Clade	Blomberg's K	p-value for K	OLS/ Pgls	Slope	Std. error	Intercep t	Std. error	LogLikelihood
			OLS	0.8096	0.0032	-0.67278	0.0066	1714.8
AVES	3.4117	0.01	**PGLS**	**0.5798**	**0.00743**	**0.01085**	**0.1176**	**2880.8**

Predicted egg size in a MOR 1125 sized T.rex (6100 kg; Persons et al. 2015) based on OLS regression for crown group birds is 66313g
Predicted egg size in a 6100 kg T.rex based on PGLS regression for birds is 8817 g with lower 95% prediction interval of 2121 g and and an upper 95% prediction bound of 40949 g

			OLS	**0.2747 7**	**0.04528**	**0.6949**	**0.20843**	**23.989**
CROCODYLIA	0.32	0.003	PGLS	0.1979	0.049	1.03121	0.2503	22.99

Predicted egg size for a 6100 kg T.rex based on OLS regression for crocs is 362g with a 95% prediction interval of 244 g to 538 g.

Predicted egg size for a 6100 kg T.rex based on PGLS regression for crocodylian reproductive data is 246g

Log transformed clutch mass as a function of log transformed body mass

Clade	Blomberg's K	p-value for K	OLS/ Pgls	Slope	Std. error	Intercep t	Std. error	LogLikelihood
			OLS	0.8073	0.0052	-0.19893	0.0107	150.462
AVES	2.1628	0.01	**PGLS**	**0.6463**	**0.0127**	**0.38971**	**0.1176**	**896.75**

Predicted clutch size for a 6100 kg T.rex based on OLS regression for birds is 189984 g.
Predicted clutch size in a 6100 kg T.rex based on PGLS regression for birds is 59552 g with a 95% prediction interval of 11220 g to 467735 g

			**predine**	**0.6150 4**	**0.06892**	**0.5602**	**0.31729**	**14.324**
CROCODYLIA	0.338	0.003	PGLS	0.5789	0.0734	0.68188	0.37537	13.669

Predicted clutch size in a 6100 kg T.rex based on OLS regression for crocs is 54137 g with a 95% prediction interval of 19015 g to 154135g

Predicted clutch size for a 6100 kg T.rex based on PGLS regression for crocs is 40702 g.

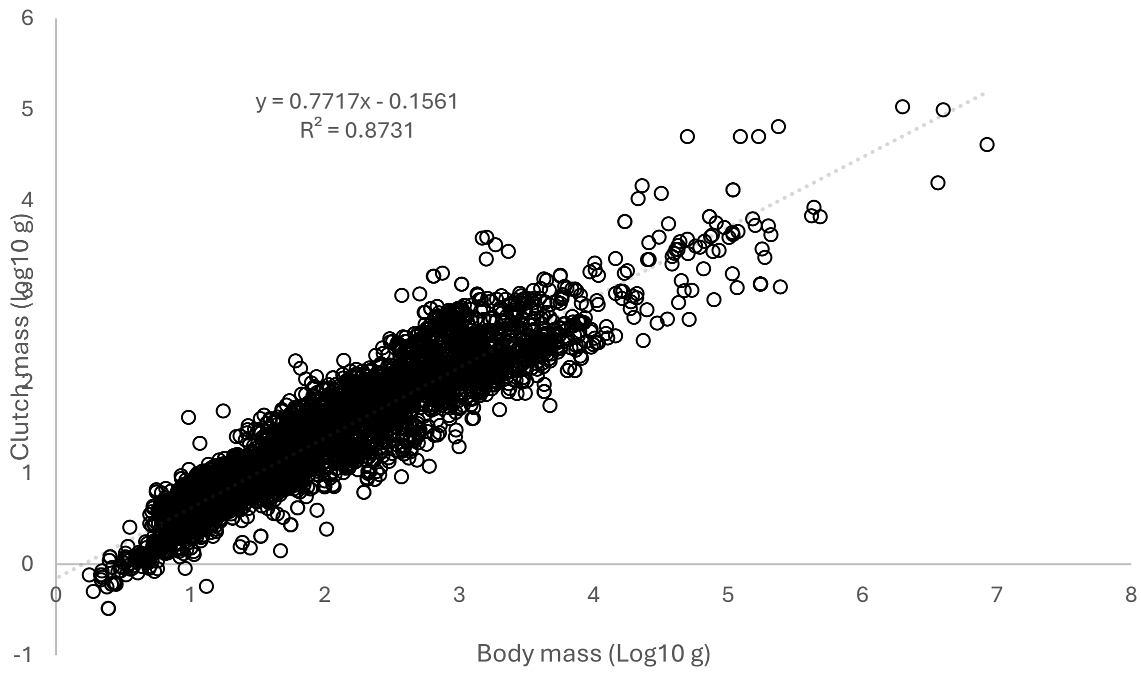

Figure S9. Clutch mass as a function of body mass for Diapsida (birds, non-avian dinosaurs, crocodilians, turtles, and squamates). The gray line represents the best fit regression. Log Clutch mass = 0.770568 * Log Body Mass - 0.154348.

5. Synchrotron microCT scanning, reconstruction, and interpretation

Reconstructed data from the synchrotron scans are available on MorphoSource. All elements were scanned at the Canadian Light Source (CLS) synchrotron (Saskatoon, SK, Canada), on the BMIT ID (BioMedical Imaging and Therapy Insertion Device) beam line and SOE-1 end station.

Scanning. Specimens were mounted in x-ray translucent plexiglass cylinders of appropriate respective diameters. Plexiglass screws held RSKM 2641.1 (the larger juvenile *Tyrannosaurus*) upright within its container. Scans were 180 degrees rotation for both hatchling metatarsals (RSKM P2416.82 and TMP 1981.16.475) and the embedded drill core of *Gorgosaurus libratus* (UALVP 49310). To enlarge the field of view for the large diameter of RSKM 2641.1, the specimen processed about an offset center of rotation for 360 degrees (a procedure called half-acquisition mode).

We applied phase-contrast x-ray imaging rather than x-ray attenuation, because phase contrast better clarifies boundaries between objects at a given resolution (Dunlop et al., 2011; Parsons et al., 2008). All scanning was at 4.4 micron pixel size at 50 keV for each projection image, with 80-500 ms exposure time. Flat- and dark field projections (10 each) were taken to correct for image noise in NRecon (Bruker).

Reconstruction. Processing of raw data was applied on custom SkyScan high performance computing clusters (CLS). We carried out 3D visualization on custom Windows workstations, and Apple iMac and Mac Pro computers (72 and 256 GB RAM, respectively), all with 4-16 GB of VRAM.

Tomographic reconstructions from raw data were realized through custom macros in NRecon and ImageJ (National Institutes of Health, USA) (Pratt et al., 2018); minimal reduction of ring artifacts was necessary. Because resolution depends on contrast between adjacent pixels/voxels, tomographic object resolution is 1/3 that of projection pixel size. For our scans the reconstructed object resolution was 13.2 microns. Reconstructed tomographic (slice) output was in 16-bit grayscale, reduced to 8-bit as needed for 3D visualization. This reduction to 8-bit halves the data size for efficient visualization processing. Phase-contrast microCT ensures that the reduced dynamic resolution does not appreciably affect object resolution, enabling fine-scale 3D reconstruction.

Bone and canals were reconstructed in 3D using Amira and Avizo (Thermofisher), by a custom protocol: Reconstructed CT data is first thresholded for grey levels corresponding to bone to create a "material," and "remove islands" applied to fill canals. The bone material exclusively is then thresholded for darker grey levels corresponding to canals, and the selection applied to a new material for the canals. An isosurface is created for the canals, with minimal smoothing to preserve detail. Surrounding bone is then imaged with a volume rendering, and that rendering's opacity manipulated to visualize the canal isosurface at the desired clarity. Volume rendering opacities of 0.4 reveal vessels near the surface, and of 0.04 depict internal canals in full while maintaining visual context of the full bone volume.

Interpretation. Synchrotron phase-contrast microCT introduces trade-offs of interpretation in comparison with ground thin sections. Individual sections from our scanning are 4.4 microns

thick in any sampled plane, compared with a typical 30-50 microns in single planes for ground thin sections. This fine-scale sampling complicates interpreting any one slice as from a given tissue type, or identifying structures as traditionally classified osteons or Haversian systems. Conversely, translucent thin sections have sharper object resolution under high magnification. Although phase contrast resolves cement lines and often osteocyte lacunae at 4.4 micron resolution, it did not resolve osteon lamellae in our scanning.

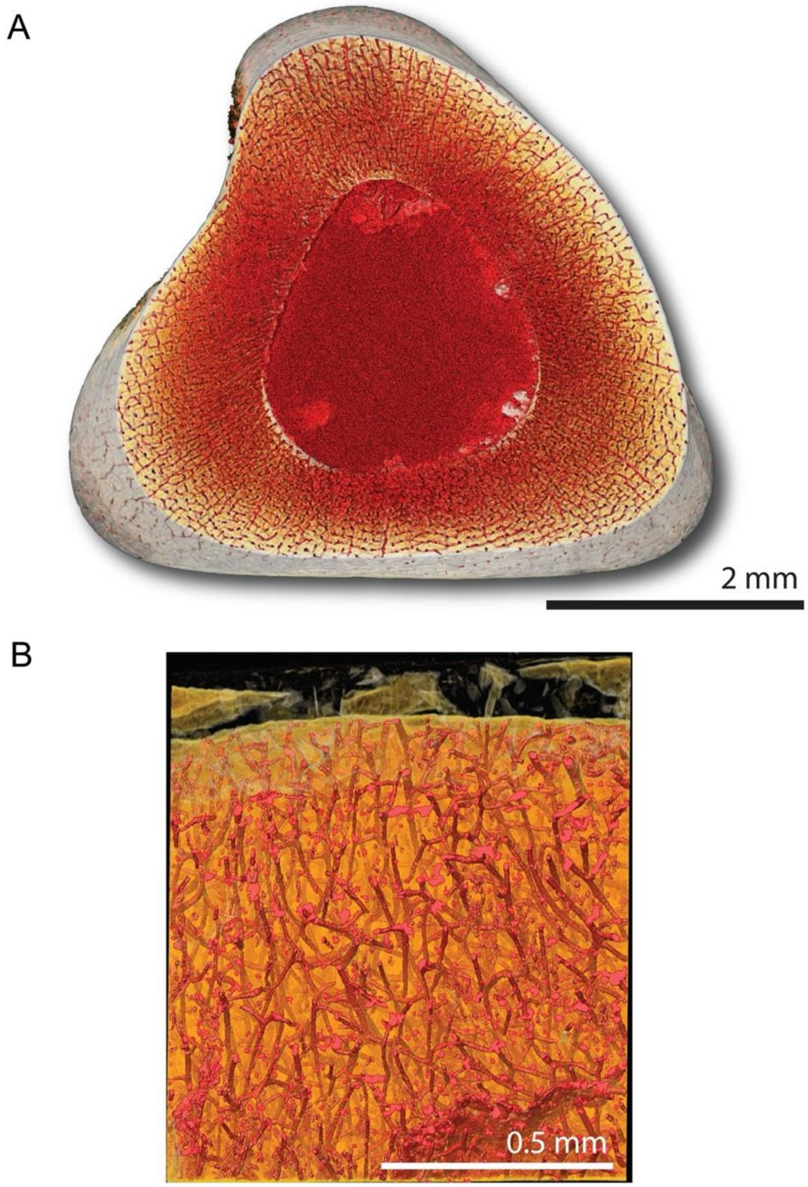

Figure S10. Vascular histology of hatchling *Gorgosaurus libratus* (TMP 1981.16.475). a, Proximal view of third metatarsal, with regularly arrayed primary canals (posterior is at apex). **B,** Lateral view of drill core of *Gorgosaurus libratus* adult (UALVP 49310) MT III, with irregular Haversian remodelling canals and no primary canals.

We therefore refer to structures as primary or secondary (remodelling) canals, rather than as primary or secondary osteons, despite the temptation to equate the terminology. Osteon and tissue classification (e.g. woven, fibrolamellar, Haversian) is contingent on three dimensional ontogeny, which is better revealed through 3D reconstruction than inferred from thin sections. MicroCT and 3D reconstruction complement ground thin sections by directly revealing canal directionality and connectivity. For example, primary (modelling) canals have variable (Figure S10) but smaller diameter, and often tighter connectivity evident in 3D reconstruction, than definitive Haversian canals as in adult *Gorgosaurus* (Surring et al. 2022).

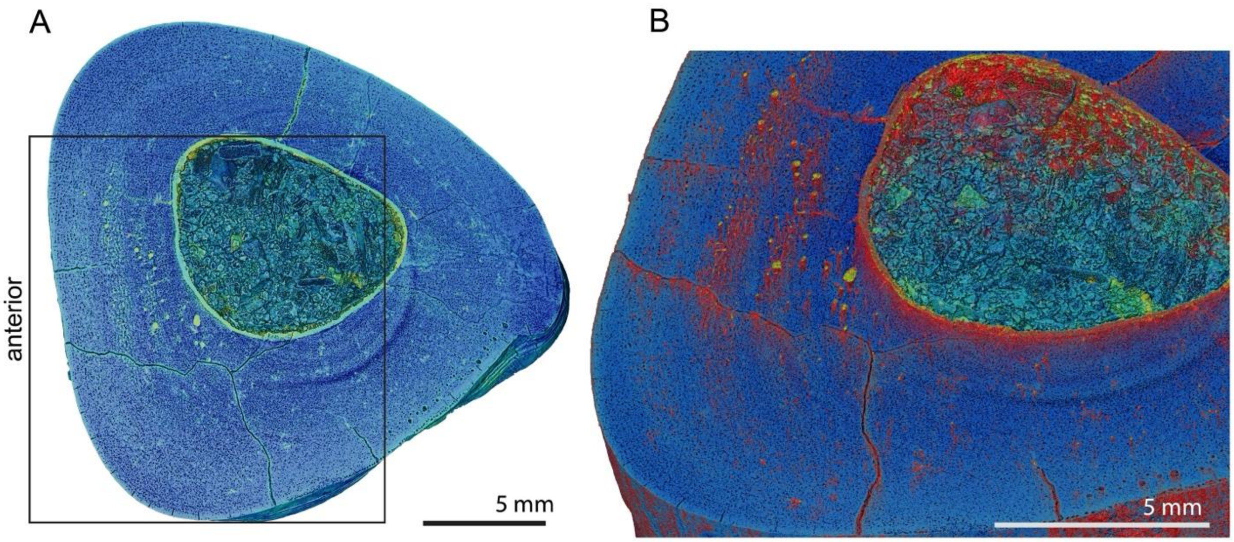

Figure S11. Synchrotron-scanned microCT reconstruction of *Tyrannosaurus rex* RSKM P2393. A. middle of preserved shaft. The bone is 2 cm wide. **B.** Closeup of zonation suggest two major annuli, for an estimated age of 3 years.

6. Bone density profiles

Bone density profiles were examined in ImageJ, using image brightness as a proxy for bone density. Surface maps were generated as well as transects through the radius of the bones. Density was plotted against distance, in pixels, from the center of the bone.

The approach was validated using scan data for hatchlings of the titanosaur *Rapetosaurus*, in which the hatching line has previously been identified [19]. At the approximate position of the hatching line, bone density begins to increase; the same pattern was seen in two different elements and occurs roughly the same distance from the center in each (Figures S12-S14)

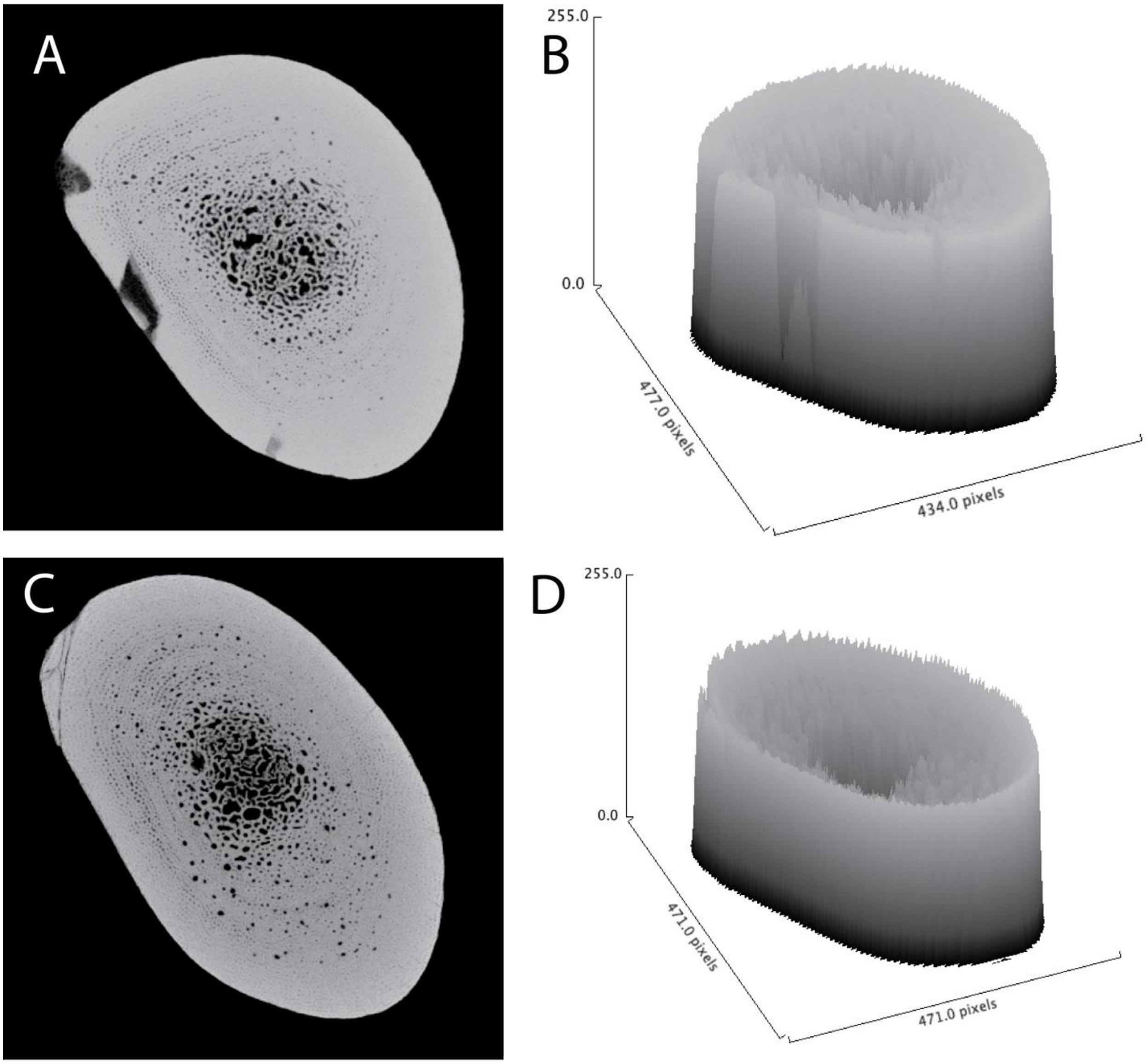

Figure S12. Bone density of hatchling *Rapetosaurus krausei*. A, CT section of femur of UA9998, B, surface map of density (gray value); C, tibia of UA9998, D, surface map of density.

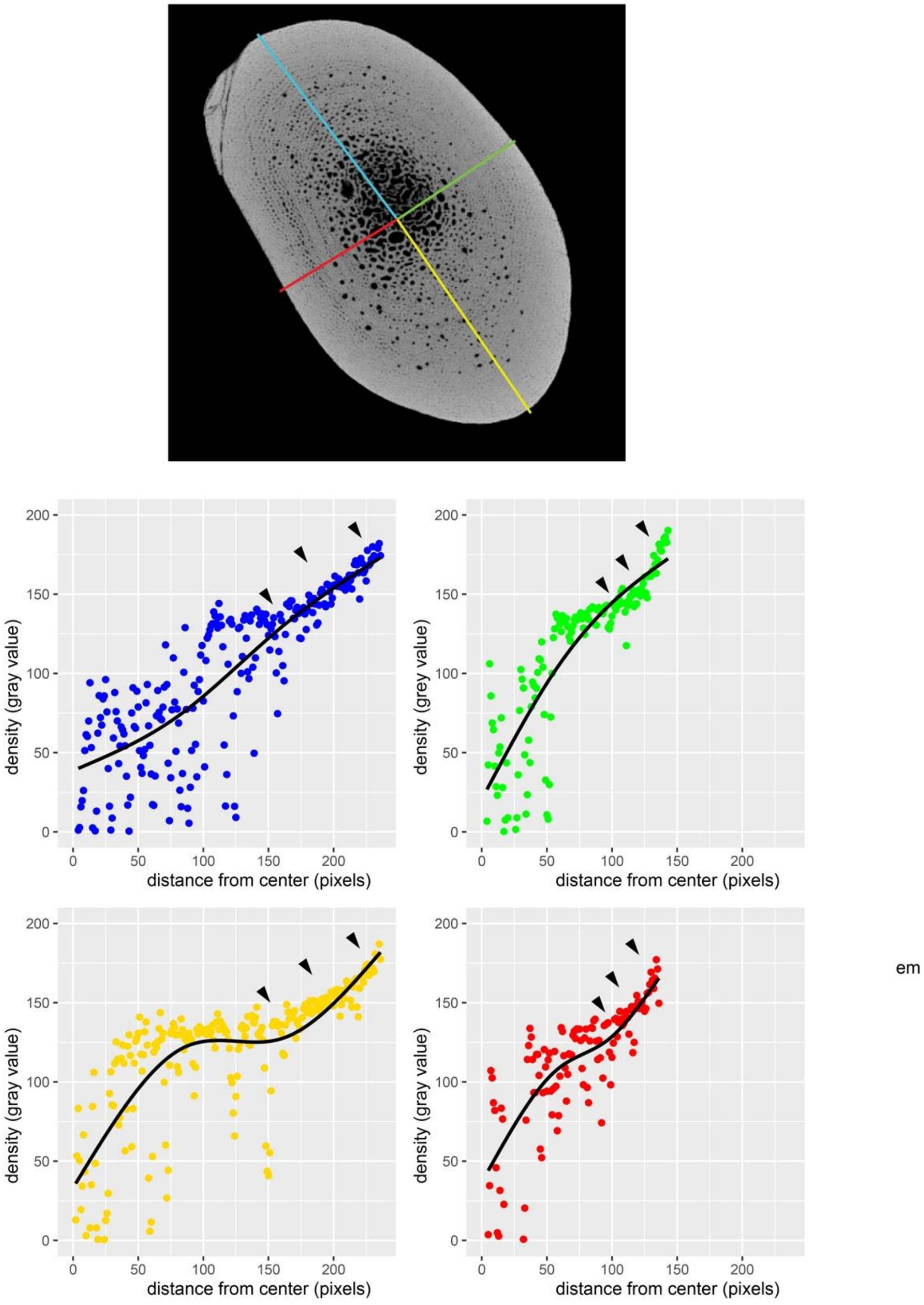

Figure S13. Bone density of hatchling *Rapetosaurus krausei*. Top, CT section of tibia of UA9998 showing position of transects taken in ImageJ [18]. Bottom, density (pixel gray value) versus distance from the center of the bone (pixels). Image courtesy Kristi Currie-Rogers.

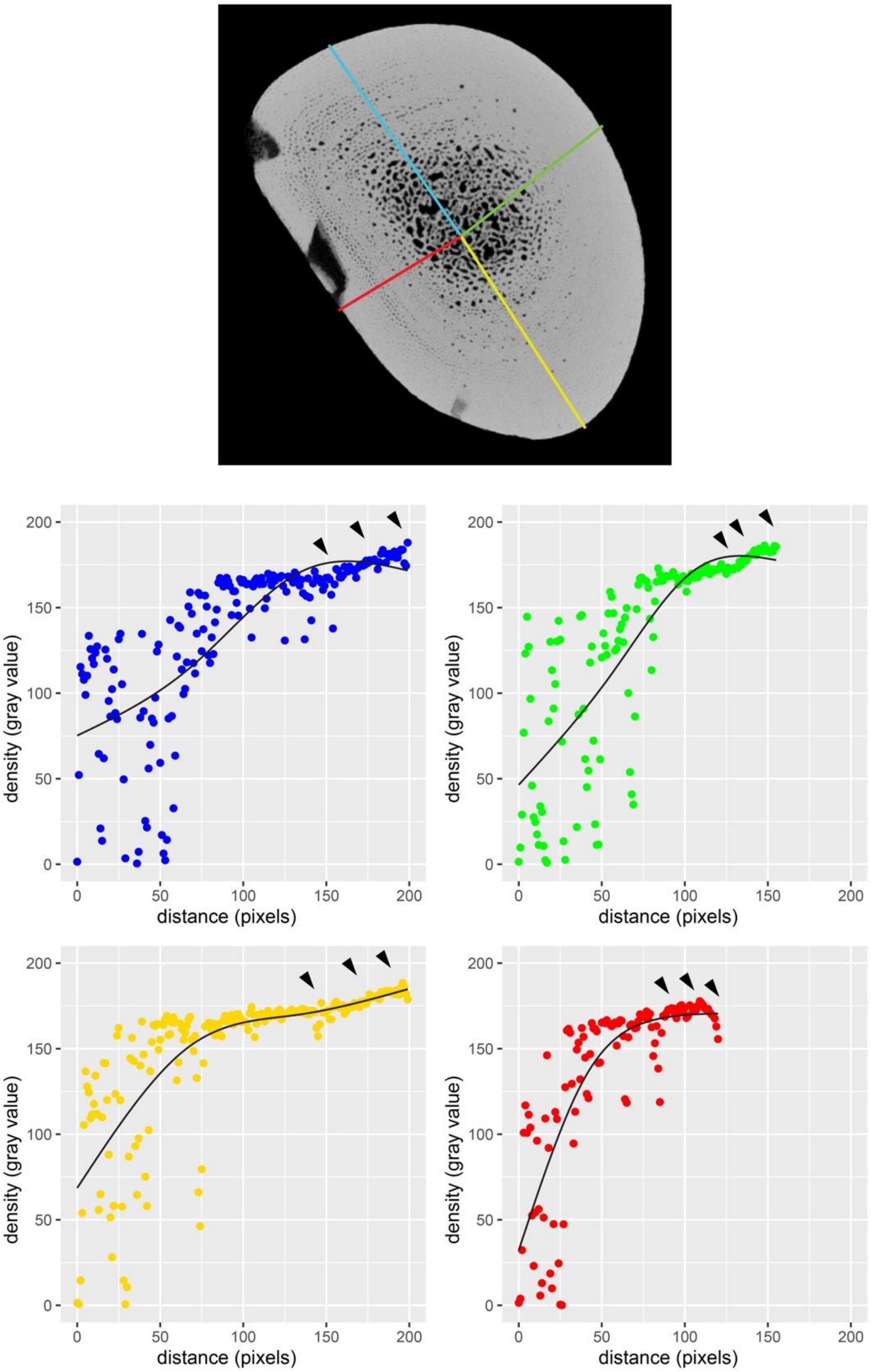

Figure S14. Bone density of hatchling *Rapetosaurus krausei*. Top, CT section of femur of UA9998 showing position of transects taken in ImageJ [18]. Bottom, density (pixel gray value) versus distance from the center of the bone (pixels). Image courtesy Kristi Currie-Rogers.

7. Fossil Provenance

Fossils described here are part of the collections of the Royal Saskatchewan Museum (RSKM), Yale Peabody Museum (YPM), and Field Museum of Natural History (FMNH).

All were collected by these institutions with the exception of one specimen, FMNH PR 4920, collected by Rob Sula from the Hell Creek Formation on July 15th, 2008 from exposures of the upper Hell Creek, from privately held land in Bowman County, North Dakota, and acquired by the Field Museum in 2017.

8. References

1. Holtz, T.R. Tyrannosauroidea. In *The Dinosauria*, Second ed.; Weishampel, D.B., Dodson, P., Osmolska, H., Eds.; University of California Press: Berkeley, 2004; pp. 111-136.
2. Brusatte, S.L.; Norell, M.A.; Carr, T.D.; Erickson, G.M.; Hutchinson, J.R.; Balanoff, A.M.; Bever, G.S.; Choiniere, J.N.; Makovicky, P.J.; Xu, X. Tyrannosaur paleobiology: new research on ancient exemplar organisms. *Science* **2010**, *329*, 1481-1485.
3. Nesbitt, S.J.; Denton, R.K.; Loewen, M.A.; Brusatte, S.L.; Smith, N.D.; Turner, A.H.; Kirkland, J.I.; McDonald, A.T.; Wolfe, D.G. A mid-Cretaceous tyrannosauroid and the origin of North American end-Cretaceous dinosaur assemblages. *Nature Ecology & Evolution* **2019**, *3*, 892-899.
4. Brusatte, S.L.; Carr, T.D. The phylogeny and evolutionary history of tyrannosauroid dinosaurs. *Scientific Reports* **2016**, *6*, 20252.
5. Longrich, N.R.; Horner, J.R.; Erickson, G.M.; Currie, P.J. Cannibalism in *Tyrannosaurus rex*. *PLOS One* **2010**, *5*, e13419.
6. Erickson, G.M.; Van Kirk, S.D.; Su, J.; Levenston, M.E.; Caler, W.E.; Carter, D.R. Bite-force estimation for *Tyrannosaurus rex* from tooth-marked bones. *Nature* **1996**, *382*, 706-708.
7. Erickson, G.M.; Makovicky, P.J.; Currie, P.J.; Norell, M.A.; Yerby, S.A.; Brochu, C.A. Gigantism and comparative life-history parameters of tyrannosaurid dinosaurs. *Nature* **2004**, *430*, 772-775.
8. Carr, T.D.; Williamson, T.E. Diversity of late Maastrichtian Tyrannosauridae (Dinosauria: Theropoda) from western North America. *Zoological Journal of the Linnean Society* **2004**, *142*, 479-523.
9. Carr, T.D. A high-resolution growth series of *Tyrannosaurus rex* obtained from multiple lines of evidence. *PeerJ* **2020**, *8*, e9192.
10. Carr, T.D. Craniofacial ontogeny in Tyrannosauridae (Dinosauria, Coelurosauria). *Journal of Vertebrate Paleontology* **1999**, *19*, 497-520.
11. Longrich, N.R.; Currie, P.J. A microraptorine (Dinosauria-Dromaeosauridae) from the Late Cretaceous of North America. *PNAS* **2009**, *106*, 5002-5008.
12. Tsuihiji, T.; Watabe, M.; Tsogtbaatar, K.; Tsubamoto, T.; Barsbold, R.; Suzuki, S.; Lee, A.H.; Ridgely, R.C.; Kawahara, Y.; Witmer, L.M. Cranial osteology of a juvenile specimen of *Tarbosaurus bataar* (Theropoda, Tyrannosauridae) from the Nemegt Formation (Upper Cretaceous) of Bugin Tsav, Mongolia. *Journal of Vertebrate Paleontology* **2011**, *31*, 497-517.
13. Funston, G.F.; Powers, M.J.; Whitebone, S.A.; Brusatte, S.L.; Scannella, J.B.; Horner, J.R.; Currie, P.J. Baby tyrannosaurid bones and teeth from the Late Cretaceous of western North America. *Canadian Journal of Earth Sciences* **2021**, *58*, 756-777.

14. Carpenter, K. Baby dinosaurs from the Late Cretaceous Lance and Hell Creek formations and a description of a new species of theropod. *Contributions to Geology, University of Wyoming* **1982**, *20*, 123-134.
15. Pratt, I.V.; Johnston, J.D.; Walker, E.; Cooper, D.M. Interpreting the three-dimensional orientation of vascular canals and cross-sectional geometry of cortical bone in birds and bats. *Journal of Anatomy* **2018**, *232*, 931-942.
16. Wolff, J. Das gestetz der transformation der knochem. *Verlag von August Hirschwald* **1892**.
17. Pearson, O.M.; Lieberman, D.E. The aging of Wolff's "law": ontogeny and responses to mechanical loading in cortical bone. *American Journal of Physical Anthropology* **2004**, *125*, 63-99.
18. Collins, T.J. ImageJ for microscopy. *Biotechniques* **2007**, *43*, S25-S30.
19. Curry Rogers, K.; Whitney, M.; D'Emic, M.; Bagley, B. Precocity in a tiny titanosaur from the Cretaceous of Madagascar. *Science* **2016**, *352*, 450-453.
20. Myhrvold, N.P.; Baldridge, E.; Chan, B.; Sivam, D.; Freeman, D.L.; Ernest, S.M. An amniote life-history database to perform comparative analyses with birds, mammals, and reptiles: Ecological Archives E096-269. *Ecology* **2015**, *96*, 3109-3109.
21. Persons IV, W.S.; Currie, P.J.; Erickson, G.M. An older and exceptionally large adult specimen of *Tyrannosaurus rex*. *The Anatomical Record* **2020**, *303*, 656-672.
22. Schweitzer, M.H.; Wittmeyer, J.L.; Horner, J.R. Gender-specific reproductive tissue in ratites and *Tyrannosaurus rex*. *Science* **2005**, *308*, 1456-1460.
23. Campione, N.E.; Evans, D.C.; Brown, C.M.; Carrano, M.T. Body mass estimation in non-avian bipeds using a theoretical conversion to quadruped stylopodial proportions. *Methods in Ecology and Evolution* **2014**, *5*, 913-923.
24. Jetz, W.; Thomas, G.; Joy, J.; Hartmann, K.; Mooers, A. The global diversity of birds in space and time. *Nature* **2012**, *491*, 444-448.
25. Hackett, S.J.; Kimball, R.T.; Reddy, S.; Bowie, R.C.; Braun, E.L.; Braun, M.J.; Chojnowski, J.L.; Cox, W.A.; Han, K.-L.; Harshman, J. A phylogenomic study of birds reveals their evolutionary history. *Science* **2008**, *320*, 1763-1768.
26. Colston, T.J.; Kulkarni, P.; Jetz, W.; Pyron, R.A. Phylogenetic and spatial distribution of evolutionary diversification, isolation, and threat in turtles and crocodilians (non-avian archosauromorphs). *BMC Evolutionary Biology* **2020**, *20*, 1-16.
27. Blomberg, S.P.; Garland Jr, T.; Ives, A.R. Testing for phylogenetic signal in comparative data: behavioral traits are more labile. *Evolution* **2003**, *57*, 717-745.
28. Owen, R. Report on British fossil reptiles. Part II. *Report of the British Association for the Advancement of Science* **1842**, *11*, 60-204.
29. Marsh, O.C. Classification of the Dinosauria. *American Journal of Science* **1881**, *23*, 81-86.
30. Huene, F.v. The dinosaurs not a natural order. *American Journal of Science (Series 4)* **1914**, *38*, 145-146.
31. Osborn, H.F. *Tyrannosaurus*, Upper Cretaceous carnivorous dinosaur (second communication). *Bulletin of the American Museum of Natural History* **1906**, *22*, 281-296.
32. Osborn, H.F. *Tyrannosaurus* and other Cretaceous carnivorous dinosaurs. *Bulletin of the American Museum of Natural History* **1905**, *35*, 733-771.

33. Fowler, D.W. Revised geochronology, correlation, and dinosaur stratigraphic ranges of the Santonian-Maastrichtian (Late Cretaceous) formations of the Western Interior of North America. *PLOS One* **2017**, *12*, e0188426.
34. Brochu, C.A. Osteology of *Tyrannosaurus rex*: insights from a nearly complete skeleton and high-resolution computed tomographic analysis of the skull. *Journal of Vertebrate Paleontology, Memoirs* **2003**, *7*, 1-138.
35. Makovicky, P.J.; Kobayashi, Y.; Currie, P.J. Ornithomimosauria. In *The Dinosauria*, Weishampel, D.B., Dodson, P., Osmolska, H., Eds.; University of California Press: Berkeley, 2004; pp. 137-150.
36. Claessens, L.P.; Loewen, M.A. A redescription of *Ornithomimus velox* Marsh, 1890 (Dinosauria, Theropoda). *Journal of Vertebrate Paleontology* **2016**, *36*, e1034593.
37. Longrich, N.R.; Currie, P.J. *Albertonykus borealis*, a new alvarezsaur (Dinosauria: Theropoda) from the Early Maastrichtian of Alberta, Canada: Implications for the systematics and ecology of the Alvarezsauridae. *Cretaceous Research* **2009**, *30*, 239-252.
38. Currie, P.J.; Russell, D.A. Osteology and relationships of *Chirostenotes pergracilis* (Saurischia, Theropoda) from the Judith River (Oldman) Formation of Canada. *Canadian Journal of Earth Sciences* **1988**, *25*, 972-986.
39. Yun, C.-G. A juvenile metatarsal of cf. *Daspletosaurus torosus*: implications for ontogeny in tyrannosaurid theropods. *Acta Palaeontologica Romaniae* **2021**, *17*, 15-22.
40. Zanno, L.E.; Napoli, J.G. *Nanotyrannus* and *Tyrannosaurus* coexisted at the close of the Cretaceous. *Nature* **2025**, 1-3.
41. Brusatte, S.L.; Carr, T.D.; Norell, M.A. The osteology of *Alioramus*, a gracile and long-snouted tyrannosaurid (Dinosauria: Theropoda) from the Late Cretaceous of Mongolia. *Bulletin of the American Museum of Natural History* **2012**, *2012*, 1-197.
42. van der Reest, A.J.; Currie, P.J. Troodontids (Theropoda) from the Dinosaur Park Formation, Alberta, with a description of a unique new taxon: implications for deinonychosaur diversity in North America. *Canadian Journal of Earth Sciences* **2017**, *54*, 919-935.
43. Longrich, N.R.; Barnes, K.; Clark, S.; Millar, L. Caenagnathidae from the Upper Campanian Aguja Formation of West Texas, and a revision of the Caenagnathinae. *Bulletin of the Peabody Museum of Natural History* **2013**, *54*, 23-49.
44. Mallon, J.C.; Bura, J.R.; Schumann, D.; Currie, P.J. A problematic tyrannosaurid (Dinosauria: Theropoda) skeleton and its implications for tyrannosaurid diversity in the Horseshoe Canyon Formation (Upper Cretaceous) of Alberta. *The Anatomical Record* **2020**, *303*, 673-690.
45. Ostrom, J.H. Osteology of *Deinonychus antirrhopus*, an unusual theropod from the Lower Cretaceous of Montana. *Peabody Museum of Natural History, Bulletin* **1969**, *30*, 1-165.
46. Longrich, N.R.; Saitta, E.T. Taxonomic status of *Nanotyrannus lancensis* (Dinosauria: Tyrannosauroidea)—a distinct taxon of small-bodied tyrannosaur. *Fossil Studies* **2024**, *2*, 1-65.
47. Ruff, C. Hindlimb articular surface allometry in Hominoidea and *Macaca*, with comparisons to diaphyseal scaling. *Journal of Human Evolution* **1988**, *17*, 687-714.

48. Godfrey, L.; Sutherland, M.; Boy, D.; Gomberg, N. Scaling of limb joint surface areas in anthropoid primates and other mammals. *Journal of Zoology* **1991**, *223*, 603-625.
49. Bishop, P.J.; Falisse, A.; De Groote, F.; Hutchinson, J.R. Predictive simulations of running gait reveal a critical dynamic role for the tail in bipedal dinosaur locomotion. *Science Advances* **2021**, *7*, eabi7348.
50. Deeming, D.; Ferguson, M. Incubation and embryonic development in reptiles and birds. In *Avian Incubation*, Tullett, S.G., Ed.; Butterworth and Co: Kent, 1990; pp. 3-37.
51. Rahn, H.; Paganelli, C. Energy budget and gas exchange of avian eggs. In *Avian Incubation*, Tullett, S.G., Ed.; Butterworth and Co: Kent, 1990; pp. 175-194.
52. Tumarkin-Deratzian, A.R.; Vann, D.R.; Dodson, P. Bone surface texture as an ontogenetic indicator in long bones of the Canada goose *Branta canadensis* (Anseriformes: Anatidae). *Zoological Journal of the Linnean Society* **2006**, *148*, 133-168.
53. DePalma, R.A.; Burnham, D.A.; Martin, L.D.; Rothschild, B.M.; Larson, P.L. Physical evidence of predatory behavior in *Tyrannosaurus rex*. *Proceedings of the National Academy of Sciences* **2013**, *110*, 12560-12564.
54. Smith, J.B. Heterodonty in *Tyrannosaurus rex*: implications for the taxonomic and systematic utility of theropod dentitions. *Journal of Vertebrate Paleontology* **2005**, *25*, 865-887.
55. Torices, A.; Reichel, M.; Currie, P.J. Multivariate analysis of isolated tyrannosaurid teeth from the Danek Bonebed, Horseshoe Canyon Formation, Alberta, Canada. *Canadian Journal of Earth Sciences* **2014**, *51*, 1045-1051.
56. Hurum, J.H.; Sabath, K. Giant theropod dinosaurs from Asia and North America: skulls of *Tarbosaurus bataar* and *Tyrannosaurus rex* compared. *Acta Palaeontologica Polonica* **2003**, *48*, 161–190.
57. Lambe, L.M. The Cretaceous Theropodous Dinosaur *Gorgosaurus*. *Canada. Department of Mines. Geological Survey Memoir* **1917**, *100*, 1-84.
58. Farlow, J.O.; Brinkman, D.L. Wear surfaces on the teeth of tyrannosaurs. *The Paleontological Society Special Publications* **1994**, *7*, 165-176.
59. Longrich, N.R. Small theropod teeth from the Lance Formation of Wyoming. In *The Unique Role of Vertebrate Microfossil Assemblages in Paleoecology and Paleobiology*, Sankey, J.T., Baszio, S., Eds.; Indiana University Press: Bloomington, IN, USA, 2008; pp. 135-158.
60. Fiorillo, A.R. On the occurrence of exceptionally large teeth of *Troodon* (Dinosauria: Saurischia) from the Late Cretaceous of northern Alaska. *PALAIOS,* **2008**, *23*, 322-328.
61. Currie, P.J. Cranial anatomy of tyrannosaurid dinosaurs from the Late Cretaceous of Alberta, Canada. *Acta Palaeontologica Polonica* **2003**, *48*, 191-226.
62. Bakker, R.; Williams, M.; Currie, P.J. *Nanotyrannus*, a new genus of pygmy tyrannosaur, from the latest Cretaceous of Montana. *Hunteria* **1988**, *1*, 1-30.
63. Larson, P. The case for *Nanotyrannus*. In *Tyrannosaurid Paleobiology*, Parrish, J.M., Molnar, R.E., Currie, P.J., Koppelhus, E.B., Eds.; Indiana University Press: Bloomington, Indiana, 2013; pp. 15-53.
64. Witmer, L.M.; Ridgely, R.C. The Cleveland tyrannosaur skull (*Nanotyrannus* or *Tyrannosaurus*): new findings based on CT scanning, with special reference to the braincase. *Kirtlandia* **2010**, *57*, 61-81.

65. Erickson, G.M.; Currie, P.J.; Inouye, B.D.; Winn, A.A. Tyrannosaur life tables: an example of nonavian dinosaur population biology. *Science* **2006**, *313*, 213-217.
66. Surring, L.; Burns, M.; Snively, E.; Barta, D.; Holtz, T.; Russell, A.; Witmer, L.; Currie, P. Consilient evidence affirms expansive stabilizing ligaments in the tyrannosaurid foot. *Vertebrate Anatomy Morphology Palaeontology* **2022**, *10*, 49-64.
67. Garcia, R.A.; Cerda, I.A. Dentition and histology in titanosaurian dinosaur embryos from Upper Cretaceous of Patagonia, Argentina. *Palaeontology* **2010**, *53*, 335-346.
68. González, R.; Cerda, I.A.; Filippi, L.S.; Salgado, L. Early growth dynamics of titanosaur sauropods inferred from bone histology. *Palaeogeography, Palaeoclimatology, Palaeoecology* **2020**, *537*, 109404.
69. Horner, J.R.; Padian, K.; de Ricqlès, A. Comparative osteohistology of some embryonic and perinatal archosaurs: developmental and behavioral implications for dinosaurs. *Paleobiology* **2001**, *27*, 39-58.
70. De Ricqlès, A.; Mateus, O.; Antunes, M.T.; Taquet, P. Histomorphogenesis of embryos of Upper Jurassic theropods from Lourinhã (Portugal). *Comptes Rendus de l'Académie des Sciences-Series IIA-Earth and Planetary Science* **2001**, *332*, 647-656.
71. Chinsamy, A.; Angst, D.; Canoville, A.; Göhlich, U.B. Bone histology yields insights into the biology of the extinct elephant birds (Aepyornithidae) from Madagascar. *Biological Journal of the Linnean Society* **2020**, *130*, 268-295.
72. Pechak, D.; Kujawa, M.; Caplan, A. Morphology of bone development and bone remodeling in embryonic chick limbs. *Bone* **1986**, *7*, 459-472.
73. Pereyra, M.E.; Bona, P.; Siroski, P.; Chinsamy, A. Ontogenetic and interelemental study of appendicular bones of Caiman latirostris Daudin, 1802 sheds light on osteohistological variability in crocodylians. *Journal of Morphology* **2024**, *285*, e21687.
74. Hugi, J.; Sánchez-Villagra, M.R. Life history and skeletal adaptations in the Galapagos marine iguana (*Amblyrhynchus cristatus*) as reconstructed with bone histological data—a comparative study of iguanines. *Journal of Herpetology* **2012**, 312-324.
75. Kidov, A.; Ivanov, A.; Ivolga, R.; Kondratova, T. Age structure and growth features of the Tajikistan toadhead agama *Phrynocephalus sogdianus* (Reptilia, Agamidae). *Proceedings of the Zoological Institute RAS* **2023**, *327*, 226-233.
76. Nacarino-Meneses, C.; Köhler, M. Limb bone histology records birth in mammals. *PLoS One* **2018**, *13*, e0198511.
77. Kitchener, J.L.; Campione, N.E.; Smith, E.T.; Bell, P.R. High-latitude neonate and perinate ornithopods from the mid-Cretaceous of southeastern Australia. *Scientific Reports* **2019**, *9*, 19600.
78. Shelton, C.D.; Sander, P.M. Long bone histology of *Ophiacodon* reveals the geologically earliest occurrence of fibrolamellar bone in the mammalian stem lineage. *Comptes Rendus Palevol* **2017**, *16*, 397-424.
79. Burr, D.B. Remodeling and the repair of fatigue damage. *Calcified Tissue International* **1993**, *53*, S75-S81.
80. Cooper, D.M.; Thomas, C.D.L.; Clement, J.G.; Hallgrímsson, B. Three-dimensional microcomputed tomography imaging of basic multicellular unit-related resorption spaces in human cortical bone. *The Anatomical Record Part A* **2006**, *288*, 806-816.

81. Christen, P.; Ito, K.; Ellouz, R.; Boutroy, S.; Sornay-Rendu, E.; Chapurlat, R.D.; Van Rietbergen, B. Bone remodelling in humans is load-driven but not lazy. *Nature Communications* **2014**, *5*, 1-5.
82. Mayya, A.; Banerjee, A.; Rajesh, R. Mammalian cortical bone in tension is non-Haversian. *Scientific Reports* **2013**, *3*, 2533.
83. Oppenheim, R.W. Prehatching and hatching behaviour in birds: a comparative study of altricial and precocial species. *Animal Behaviour* **1972**, *20*, 644-655.
84. Fabbri, M.; Mongiardino Koch, N.; Pritchard, A.C.; Hanson, M.; Hoffman, E.; Bever, G.S.; Balanoff, A.M.; Morris, Z.S.; Field, D.J.; Camacho, J. The skull roof tracks the brain during the evolution and development of reptiles including birds. *Nature Ecology & Evolution* **2017**, *1*, 1543-1550.
85. Norell, M.A.; Wiemann, J.; Fabbri, M.; Yu, C.; Marsicano, C.A.; Moore-Nall, A.; Varricchio, D.J.; Pol, D.; Zelenitsky, D.K. The first dinosaur egg was soft. *Nature* **2020**, *583*, 406-410.
86. Lee, A.H.; Werning, S. Sexual maturity in growing dinosaurs does not fit reptilian growth models. *Proceedings of the National Academy of Sciences* **2008**, *105*, 582-587.
87. Platt, S.G.; Rainwater, T.R.; Thorbjarnarson, J.B.; McMurry, S.T. Reproductive dynamics of a tropical freshwater crocodilian: Morelet's crocodile in northern Belize. *Journal of Zoology* **2008**, *275*, 177-189.
88. Ford, N.B.; Seigel, R.A. Relationships among body size, clutch size, and egg size in three species of oviparous snakes. *Herpetologica* **1989**, 75-83.
89. Herman, A.; Bout, R.G. Relationships between maternal size, egg size, clutch size, and hatchling size in European lacertid lizards. *Journal of Herpetology* **1998**, 410-417.
90. Balshine, S. Patterns of parental care in vertebrates. In *The Evolution of Parental Care*, Nick J. Royle, P.T.S., Mathias Kölliker, Ed.; 2012; Volume 62-80.
91. Brazaitis, P.; Watanabe, M.E. Crocodilian behaviour: a window to dinosaur behaviour? *Historical Biology* **2011**, *23*, 73-90.
92. Witmer, L.M. The extant phylogenetic bracket and the importance of reconstructing soft tissues in fossils. In *Functional Morphology in Vertebrate Paleontology*, Thomason, J.J., Ed.; Cambridge University Press: New York, 1995; pp. 19-33.
93. Norell, M.A.; Clark, J.M.; Weintraub, R.; Chiappe, L.M.; Demberelyin, D. A nesting dinosaur. *Nature* **1995**, *378*, 774-776.
94. Dong, Z.-M.; Currie, P.J. On the discovery of an oviraptorid skeleton on a nest of eggs at Bayan Mandahu, Inner Mongolia, People's Republic of China. *Canadian Journal of Earth Sciences* **1996**, *33*, 631-636.
95. Varricchio, D.J.; Jackson, F.; Borkowski, J.J.; Horner, J.R. Nest and egg clutches of the dinosaur *Troodon formosus* and the evolution of avian reproductive traits. *Nature* **1997**, *385*, 247-250.
96. Meng, Q.; Liu, J.; Varricchio, D.J.; Huang, T.; Gao, C. Parental care in an ornithischian dinosaur. *Nature* **2004**, *431*, 145-146.
97. Tullberg, B.S.; Ah–King, M.; Temrin, H. Phylogenetic reconstruction of parental–care systems in the ancestors of birds. *Philosophical Transactions of the Royal Society of London. Series B: Biological Sciences* **2002**, *357*, 251-257.
98. Perrins, C. *The Firefly Encyclopedia of Birds*; Firefly Books: Buffalo, 2003.

99. Reisz, R.R.; Huang, T.D.; Chen, C.-M.; Tu, S.-J.; Tsai, T.-C.; Zhong, S.; Mooney, E.D.; Bevitt, J.J. Parental feeding in the dinosaur *Lufengosaurus* revealed through multidisciplinary comparisons with altricial and precocious birds. *Scientific Reports* **2024**, *14*, 20309.
100. MacArthur, R.H.; Wilson, E.O. *The Theory of Island Biogeography*; Princeton University Press: 1967.
101. Pianka, E.R. On r-and K-selection. *The American Naturalist* **1970**, *104*, 592-597.
102. Paul, G.S. Dinosaur reproduction in the fast lane: Implications for size, success, and extinction. In *Dinosaur Eggs and Babies*, Carpenter, K., Hirsch, K.F., Horner, J.R., Eds.; Cambridge University Press: New York, 1994; pp. 244–255.
103. Rogers, R.R.; Krause, D.W.; Curry Rogers, K. Cannibalism in the Madagascan dinosaur *Majungatholus atopus*. *Nature* **2003**, *422*, 515-518.
104. Rootes, W.L.; Chabreck, R.H. Cannibalism in the American alligator. *Herpetologica* **1993**, *49*, 99-107.
105. Amstrup, S.C.; Stirling, I.; Smith, T.S.; Perham, C.; Thiemann, G.W. Recent observations of intraspecific predation and cannibalism among polar bears in the southern Beaufort Sea. *Polar Biology* **2006**, *29*, 997-1002.
106. Eloff, F. Food ecology of the Kalahari lion *Panthera leo vernayi*. *Koedoe* **1984**, *27*, 249-258.
107. Navarro, J.; Vignolo, P.; Demaria, M.R.; Maceira, N.O.; Martella, M.B. Growth curves of farmed Greater Rheas (*Rhea americana albescens*) from central Argentina. *Archiv fur Geflugelkunde* **2005**, *69*, 90-93.
108. Ramos, S.; Caetano, S.; Savegnago, R.; Nunes, B.; Ramos, A.A.; Munari, D. Growth curves for ostriches (*Struthio camelus*) in a Brazilian population. *Poultry Science* **2013**, *92*, 277-282.
109. Wilkinson, P.M.; Rhodes, W.E. Growth rates of American alligators in coastal South Carolina. *The Journal of Wildlife Management* **1997**, 397-402.
110. Farlow, J.O.; Hurlburt, G.R.; Elsey, R.M.; Britton, A.R.; Langston Jr, W. Femoral dimensions and body size of *Alligator mississippiensis*: estimating the size of extinct mesoeucrocodylians. *Journal of Vertebrate Paleontology* **2005**, *25*, 354-369.
111. Bahashwan, S.; Alrawas, A.S.; Alfadli, S.; Johnson, E. Dhofari cattle growth curve prediction by different non-linear model functions. *Livestock Research for Rural Development* **2015**, *26*, 236.
112. Mumby, H.S.; Chapman, S.N.; Crawley, J.A.; Mar, K.U.; Htut, W.; Thura Soe, A.; Aung, H.H.; Lummaa, V. Distinguishing between determinate and indeterminate growth in a long-lived mammal. *BMC Evolutionary Biology* **2015**, *15*, 1-9.
113. Maxwell, E.E.; Caldwell, M.W. First record of live birth in Cretaceous ichthyosaurs: closing an 80 million year gap. *Proceedings of the Royal Society of London. Series B: Biological Sciences* **2003**, *270*, S104-S107.
114. Cheng, Y.-n.; Wu, X.-c.; Ji, Q. Triassic marine reptiles gave birth to live young. *Nature* **2004**, *432*, 383-386.
115. O'Keefe, F.R.; Chiappe, L.M. Viviparity and 10, 49-64.-selected life history in a Mesozoic marine plesiosaur (Reptilia, Sauropterygia). *Science* **2011**, *333*, 870-873.

116. Caldwell, M.W.; Lee, M.S. Live birth in Cretaceous marine lizards (mosasauroids). *Proceedings of the Royal Society of London. Series B: Biological Sciences* **2001**, *268*, 2397-2401.
117. Cappetta, H. Chondrichthyes II. Mesozoic and Cenozoic Elasmobranchii. *Handbook of Paleoichthyology* **1987**, *3*, 1-193.
118. Hoffman, E.A.; Rowe, T.B. Jurassic stem-mammal perinates and the origin of mammalian reproduction and growth. *Nature* **2018**, *561*, 104-108.
119. Kielan-Jaworowska, Z.; Cifelli, R.L.; Luo, Z.-X. *Mammals from the Age of Dinosaurs: Origins, Evolution and Structure*; Columbia University Press: New York, 2004; p. 648.
120. Luo, Z.-X.; Yuan, C.-X.; Meng, Q.-J.; Ji, Q. A Jurassic eutherian mammal and divergence of marsupials and placentals. *Nature* **2011**, *476*, 442-445.
121. Grimaldi, D.; Engel, M.S. *Evolution of the Insects*; Cambridge University Press: Cambridge, 2005; p. 755.
122. Eriksson, O. Evolution of seed size and biotic seed dispersal in angiosperms: paleoecological and neoecological evidence. *International Journal of Plant Sciences* **2008**, *169*, 863-870.
123. dos Reis, M.; Inoue, J.; Hasegawa, M.; Asher, R.J.; Donoghue, P.C.J.; Yang, Z. Phylogenomic datasets provide both precision and accuracy in estimating the timescale of placental mammal phylogeny. *Proceedings of the Royal Society B* **2012**, *279*, 3491-3500.
124. Prum, R.O.; Berv, J.S.; Dornburg, A.; Field, D.J.; Townsend, J.P.; Lemmon, E.M.; Lemmon, A.R. A comprehensive phylogeny of birds (Aves) using targeted next-generation DNA sequencing. *Nature* **2015**.
125. Swofford, D.L. *PAUP*. Phylogenetic Analysis Using Parsimony (*and other methods)*, 4.0b10; Sinauer Associates: Sunderland, Massachusetts, 2002.
126. Holtz, T.R. The arctometatarsalian pes, an unusual structure of the metatarsus of Cretaceous Theropoda (Dinosauria: Saurischia). *Journal of Vertebrate Paleontology* **1995**, *14*, 480-519.
127. Brusatte, S.L.; Lloyd, G.T.; Wang, S.C.; Norell, M.A. Gradual assembly of avian body plan culminated in rapid rates of evolution across the dinosaur-bird transition. *Current Biology* **2014**, *24*, 2386-2392.
128. Osborn, H.F. Skeletal adaptations of *Ornitholestes*, *Struthiomimus*, *Tyrannosaurus*. *Bulletin of the American Museum of Natural History* **1917**, *35*, 733-771.
129. Osmólska, H.; Roniewicz, E.; Barsbold, R. A new dinosaur, *Gallimimus bullatus* n. gen, n. sp. (Ornithomimidae) from the Upper Cretaceous of Mongolia. *Palaeontologica Polonica* **1972**, *27*, 103-143.
130. Funston, G.; Persons IV, W.; Bradley, G.; Currie, P. New material of the large-bodied caenagnathid *Caenagnathus collinsi* from the Dinosaur Park Formation of Alberta, Canada. *Cretaceous Research* **2015**, *54*, 179-187.
131. Norell, M.A.; Makovicky, P.J. Important features of the dromaeosaurid skeleton II: information from newly collected specimens of *Velociraptor mongoliensis*. *American Museum Novitates* **1999**, *3282*, 1-45.

132. McFeeters, B.; Ryan, M.J.; Schröder-Adams, C.; Cullen, T.M. A new ornithomimid theropod from the Dinosaur Park Formation of Alberta, Canada. *Journal of Vertebrate Paleontology* **2016**, *36*, e1221415.
133. Russell, D.A. A new specimen of *Stenonychosaurus* from the Oldman Formation (Cretaceous) of Alberta. *Canadian Journal of Earth Sciences* **1969**, *6*, 595-612.
134. Funston, G.F.; Currie, P.J.; Eberth, D.A.; Ryan, M.J.; Chinzorig, T.; Badamgarav, D.; Longrich, N.R. The first oviraptorosaur (Dinosauria: Theropoda) bonebed: evidence of gregarious behaviour in a maniraptoran theropod. *Scientific Reports* **2016**, *6*.
135. Currie, P.J. Bird like characteristics of the jaws and teeth of troodontid theropods (Dinosauria:Saurischia). *Journal of Vertebrate Paleontology* **1987**, *7*, 72-81.
136. Makovicky, P.J.; Sues, H.-D. Anatomy and phylogenetic relationships of the theropod dinosaur *Microvenator celer* from the Lower Cretaceous of Montana. *American Museum Novitates* **1998**, *3249*, 1-27.
137. Maleev, E.A. Giant carnosaurs of the family Tyrannosauridae. *Joint Soviet-Mongolian Paleontological Expedition* **1974**, *1*, 132-191.
138. Currie, P.J.; Evans, D.C. Cranial anatomy of new specimens of *Saurornitholestes langstoni* (Dinosauria, Theropoda, Dromaeosauridae) from the Dinosaur Park Formation (Campanian) of Alberta. *The Anatomical Record* **2020**, *303*, 691-715.
139. Colbert, E.; Russell, D.A. The small Cretaceous dinosaur *Dromaeosaurus*. *American Museum Novitates* **1969**, *2380*, 1-49.
140. Hanai, T.; Tsuihiji, T. Description of tooth ontogeny and replacement patterns in a juvenile *Tarbosaurus bataar* (Dinosauria: Theropoda) using CT-scan data. *The Anatomical Record* **2019**, *302*, 1210-1225.
141. Hendrickx, C.; Mateus, O.; Araujo, R.; Choiniere, J. The distribution of dental features in non-avian theropod dinosaurs: Taxonomic potential, degree of homoplasy, and major evolutionary trends. *Palaeontologia Electronica* **2019**, *22*, 1-110.
142. Currie, P.J.; Rigby, K.J.; Sloan, R.E. Theropod teeth from the Judith River Formation of southern Alberta, Canada. In *Dinosaur Systematics: Perspectives and Approaches*, Currie, P.J., Carpenter, K., Eds.; Cambridge University Press: Cambridge, 1990; pp. 107-125.
143. Longrich, N.R. A new, large ornithomimid from the Dinosaur Park Formation of Alberta, Canada: implications for the study of dissociated dinosaur remains. *Palaeontology* **2008**, *51*, 983-997.
144. Carr, T.D.; Varricchio, D.J.; Sedlmayr, J.C.; Roberts, E.M.; Moore, J.R. A new tyrannosaur with evidence for anagenesis and crocodile-like facial sensory system. *Scientific Reports* **2017**, *7*, srep44942.
145. Dunning, J.B. *Handbook of Avian Body Masses*, 2 ed.; CRC Press: Boca Raton, 2007; p. 672.
146. Paul, G.S. *The Princeton Field Guide to Dinosaurs*, 2nd ed.; Princeton University Press, : Princeton, NJ, USA, 2016.
147. Alexander, R.M. *Dynamics of Dinosaurs and Extinct Giants*; Columbia University: New York, 1989; p. 167.
148. Birchard, G.; Deeming, D. Avian eggshell thickness: scaling and maximum body mass in birds. *Journal of Zoology* **2009**, *279*, 95-101.
